# A microbial consortium‐based product promotes potato yield by recruiting rhizosphere bacteria involved in nitrogen and carbon metabolisms

**DOI:** 10.1111/1751-7915.13876

**Published:** 2021-07-07

**Authors:** Zhenshuo Wang, Yan Li, Yu Zhao, Lubo Zhuang, Yue Yu, Mengyao Wang, Jia Liu, Qi Wang

**Affiliations:** ^1^ Department of Plant Pathology MOA Key Lab of Pest Monitoring and Green Management College of Plant Protection China Agricultural University Beijing 100193 China; ^2^ Chongqing Key Laboratory of Economic Plant Biotechnology College of Landscape Architecture and Life Science/Institute of Special Plants Chongqing University of Arts and Sciences Yongchuan, Chongqing 402160 China

## Abstract

The effect of a microbial consortium‐based (MCB) biocontrol product, composed of *Bacillus subtilis*, *Trichoderma harzianum* strain and diatomaceous earth as a carrier, on potato yield, and potential modes of action for its effect were investigated. The MCB product (300 kg ha^−1^) was added to furrows in which the potato seed tubers each year for 3 years (2016, 2017 and 2018), while potato planting without the MCB product treatment served as the control. A metagenomic analysis indicated that bacterial phylotypes dominated the microbial community, with a relatively small contribution of archaea and fungal taxa. The relative abundance of beneficial bacterial taxa increased significantly in response to the MCB product treatment. Notably, a higher relative abundance of bacterial taxa with carbon fixation, carbon‐degrading and nitrogen metabolism properties were observed in the MCB product‐treated potato rhizosphere. This was also reflected in the identification of a greater abundance of genes encoding enzymes involved in nitrogen metabolism, carbon fixation and carbon degradation pathways in the conducted metagenomic analysis. The greater relative abundance of these beneficial bacterial taxa in the rhizosphere of MCB product‐treated plots, as well as the higher abundance of genes associated with the indicated cellular processes, were associated with an increase in tuber yield. The observed changes in microbial community structure at an early stage of tuber development appears to have a beneficial impact on tuber yield.

## Graphical abstract

A microbial‐consortium‐based product applied to soil increased potato tuber yield by fostering the recruitment and proliferation of beneficial bacteria, especially Proteobacteria with nitrogen‐transforming capacity. The functional potential of the potato rhizosphere bacterial community was enriched in taxa with genes involved with nitrogen and carbon metabolism, as well as carbon degradation.

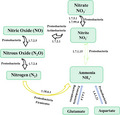

## Introduction

Potato (*Solanum tuberosum* L.) is a non‐cereal food crop whose worldwide production has been increasing, ranking as the fourth highest produced food crop after wheat, maize and rice, with global potato production estimated to be 368 million tonnes in 2018 (FAO, 2018). Potatoes are cultivated in over 100 countries, feeding more than a billion people, and serve as a primary food staple for hundreds of millions of people. Therefore, improving potato crop productivity and quality can address the nutritional needs of a rising population (Dahal *et␣al*., 2018; Muleta and Aga, 2019). Various methods, such as the use of plant residues, chemical fertilizers, soil conditioners and beneficial microbial products, have been utilized to improve potato yield (Paul *et␣al*., 2016; Zheng *et␣al*., 2019). Using biocontrol agents is of great interest as this approach is eco‐friendly and conforms with sustainable agricultural practices (Khan *et␣al*., 2019). Several species of bacteria and fungi have been reported to promote plant growth and improve crop yield, including *Bacillus* spp., *Streptomyces* spp., *Pseudomonas* spp. and *Trichoderma* spp., functioning as biofertilizers (Devi *et␣al*., 2016; Alori *et␣al*., 2017). Several mechanisms have been proposed or demonstrated to explain the beneficial impact of these microorganisms on growth and yield, including the direct promotion of plant growth by facilitating resource acquisition (nitrogen, phosphorus and essential minerals) or modulating plant hormone levels, or indirectly by decreasing the inhibitory impact of phytopathogens through the activity of biocontrol agents, including competition; the production of antibiotics, cell wall‐degrading enzymes, hydrogen cyanide and siderophores; the induction of systemic resistance and quorum quenching (Devi *et␣al*., 2016; Alori *et␣al*., 2017; Olanrewaju *et␣al*., 2017; Khan *et␣al*., 2019). Further details on the growth‐promoting mechanisms exhibited by biocontrol agents, especially when a consortium is used, still requires further study.

Plants obtain a portion of the nutrients needed for growth via the beneficial interactions that exist between soil microbes and roots (Edwards *et␣al*., 2015). Microbes can process nutrients so that they are in forms that are readily absorbed by plants (Alori *et␣al*., 2017; Zhong *et␣al*., 2018). Rhizosphere microorganisms play an important role in plant production. They promote plant growth through nutrient acquisition, carbon cycling, nitrogen fixation and cycling, and the biosynthesis of phytohormones (Aloo *et␣al*., 2019; Chen *et␣al*., 2019). Microbial‐based approaches are alternatives to improving crop yield (Oleńska *et␣al*., 2020). Studies have reported that the application of microbial strains of *Bacillus* spp. and *Trichoderma* spp. can increase crop yield and alter the composition of the rhizosphere microbial community in sorghum, corn and potato (Ding *et␣al*., 2012; Saravanakumar *et␣al*., 2017; Wu *et␣al*., 2019). Soil microbes can potentially play a role in improving crop yield by recruiting or fostering the increased abundance of taxa with beneficial nitrogen metabolism activity. Therefore, clarifying the role of soil microbes in improving yield could assist in the selection of microbial taxa or other methods that could further increase crop yield and quality.

Our previous two‐year study demonstrated that potato tubers planted in soils that were treated with 300 kg ha^−1^ of a microbial consortium‐based (MCB) product composed of *Bacillus subtilis* (strain znjdf1), *Trichoderma harzianum* strain (strain znlkhc1) and diatomaceous earth as a carrier and exhibited a higher relative abundance of beneficial rhizosphere bacteria, relative to untreated potato plants, which was positively correlated with tuber yield (Wang *et␣al*., 2019a). A comprehensive analysis of the mechanisms associated with the higher potato yield, however, remains to be conducted. Therefore, we initiated a field study in 2018 using the same MCB product, applied at 300 kg ha^−1^ and compared the structure of the microbial community with untreated control plots. A comprehensive characterization of the soil microbiome was performed using metagenomic sequencing to assess (i) the composition of the microbial community function in soils treated with the MCB product, relative to untreated soils, and (ii) gain insights into the properties of the microbial community that would potentially promote an increase in tuber yield. More specifically, we investigated the effect of the MCB product on the composition of the microbial community (bacteria, fungi and archaea) and the capacity of that microbial community to affect nitrogen metabolism, carbon fixation and carbon degradation in the rhizosphere microbiome. Results of the study provide an improved understanding of the mechanisms underlying the ability of the selected MCB product, containing a known beneficial bacterium and fungus, along with diatomaceous earth as a carrier, to improve the productivity of potato. These data can be used to further develop biological methods that enhance tuber yield.

## Results

### The MCB product increased potato tuber yield

In this study, bulk soil samples were collected from untreated plots and plots treated with 300 kg ha^−1^ of the MCB product 3 days prior to planting. These samples are referred to as T1CK and T1B300 respectively. Rhizosphere soil samples (collected at the time of early tuber formation) were also obtained from untreated plots and plots treated with 300 kg ha^−1^ of the MCB product. These are referred to as T2CK and T2B300 respectively. Potato yield in the B300 treatment plot in 2018 was 49 136.05 kg ha^−1^, which was significantly higher than the 30 037.19 kg ha^−1^ in the no treatment control (CK), which was not administered the MCB product treatment. These results indicate that the application of the MCB product increased potato yield by 63.58% (Fig. S1A). Similar differences in potato tuber yield were obtained in 2016 and 2017 (Wang *et␣al*., 2019a). Soil organic carbon (SOC) and total nitrogen (TN) were significantly higher in the T2B300 samples, relative to the untreated T2CK (Fig. S1B and C) samples.

### Soil microbiome community composition and structure

**Fig. 1:**
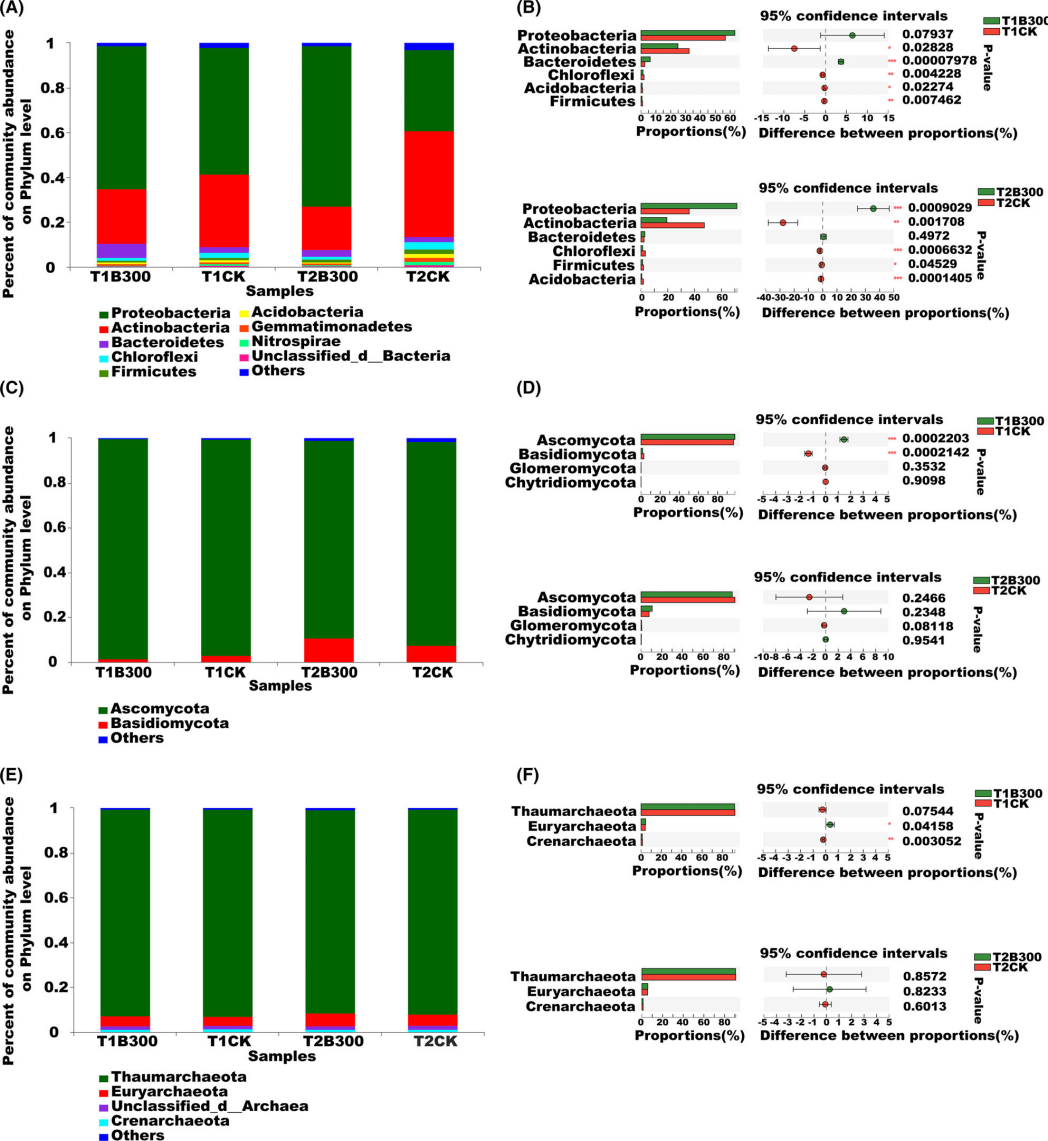
Comparative analysis of the dominant microbial taxa in bulk and potato rhizosphere soil samples collected from untreated (CK) and MCB product treatment (300 kg ha^−1^) plots. A. Relative abundance of the most abundant bacterial phyla. B. Bacterial phyla with a significantly different levels of relative abundance. Taxonomic profile of bacterial phyla whose relative abundance was different between the 300 kg ha^−1^ MCB product treatment and the CK plots prior to planting (T1, upper panel) and at early tuber formation (T2, lower panel). C. Relative abundance of the most abundant fungal phyla. D. Fungal phyla with significantly different levels of relative abundance. Taxonomic profile of fungal phyla whose relative abundance was different between the 300 kg ha^−1^ MCB product treatment and CK plots prior to planting (T1, upper panel) and at early tuber formation (T2, lower panel). E. Relative abundance of the most abundant archaeal phyla. F. Archaeal phyla with significantly different levels of relative abundance. Taxonomic profile of archaeal phyla whose relative abundance was different between the 300 kg ha^−1^ MCB product treatment and CK plots prior to planting (T1, upper panel) and at early tuber formation (T2, lower panel). *, ** and *** indicate a significant correlation at *P* < 0.05, *P* < 0.01 and *P* < 0.001 respectively. T1CK and T1B300 indicate bulk soil samples collected from CK and B300 treatment plots, respectively, 3 days prior to planting. T2CK and T2B300 indicate rhizosphere soil samples collected from CK and 300 kg ha^−1^ treatment plots, respectively, at the time of early tuber formation.

One of the objectives of the present study was to characterize the response of the microbial community, as well as its potential function, to the application of the MCB product. Microbial community structure and the presence of functional genes were evaluated using a metagenomic approach. The dominant bacterial phyla present in the bulk soil and their relative abundance in the CK and consortium‐treated plots, respectively, were Proteobacteria (56.7% and 63.8%), Actinobacteria (32.37% and 24.29%), Bacteroidetes (2.37% and 2.52%), Chloroflexi (2.07% and 1.34%), Acidobacteria (1.05% and 0.77%) and Firmicutes (1.04–0.72%) (Fig. 1A). The relative abundance of all of the bacterial phyla, except Proteobacteria, differed significantly between the treated and untreated plots. The most prevalent bacterial phyla in the rhizosphere of the CK and MCB product treatment plots and their relative abundance were Proteobacteria (36.15% and 71.54%), Actinobacteria (47.05% and 19.34%), Bacteroidetes (2.37% and 2.90%), Chloroflexi (3.28 and 1.29%), Firmicutes (1.89% and 1.23%) and Acidobacteria (1.85% and 0.66%) respectively. Only Bacteroidetes exhibited no significant difference between the T2CK and T2B300 plots (Fig. 1B). The relative abundance of Proteobacteria was significantly greater in the T2B300 samples than T2CK samples. These results indicate that the relative abundance of Proteobacteria significantly increased in response to the application of the MCB product. The dominant bacterial orders and genera in the rhizosphere and bulk soil are shown in Fig. S2. The relative abundance of the seven dominant bacterial orders (Burkholderiales, Pseudomonadales, Xanthomonadales, Rhizobiales, Enterobacteriales, Rhodospirillales and Cellvibrionales) and seven dominant bacterial genera (*Pseudomonas*, *Pseudoxanthomonas*, *Pseudoxanthomonas*, *Achromobacter*, *Delftia*, *Lysobacter* and *Azospirillum*) was higher in plots treated with the MCB product than in the untreated CK plots at the time of early tuber formation. Notably, all the orders and genera that exhibited significant differences between the treatment and CK belonged to the Proteobacteria. The unweighted paired group method with arithmetic mean (UPGMA) tree revealed that the treatments clustered into two groups based on bacterial community composition. The bacterial genera in the MCB product treatment plots clustered together and were clearly separated from the other treatment plots at the time of early tuber formation (Fig. S3). The number of bacterial genera in the bulk soil of treated and untreated plots was similar; however, the number of genera increased in rhizosphere samples and was higher than that in the bulk soil samples. This was especially true in rhizosphere soil samples of plots treated with the MCB product (Fig. S4A). The numbers of fungal and archaeal genera were similar in all soil samples (Fig. S4B and C). The relative abundance of the most highly abundant fungal and archaeal taxa differed slightly between the CK and MCB product treatment plots in bulk and rhizosphere soil samples (Fig. 1C–F, Fig. S5, Fig. S6).

**Fig. 2:**
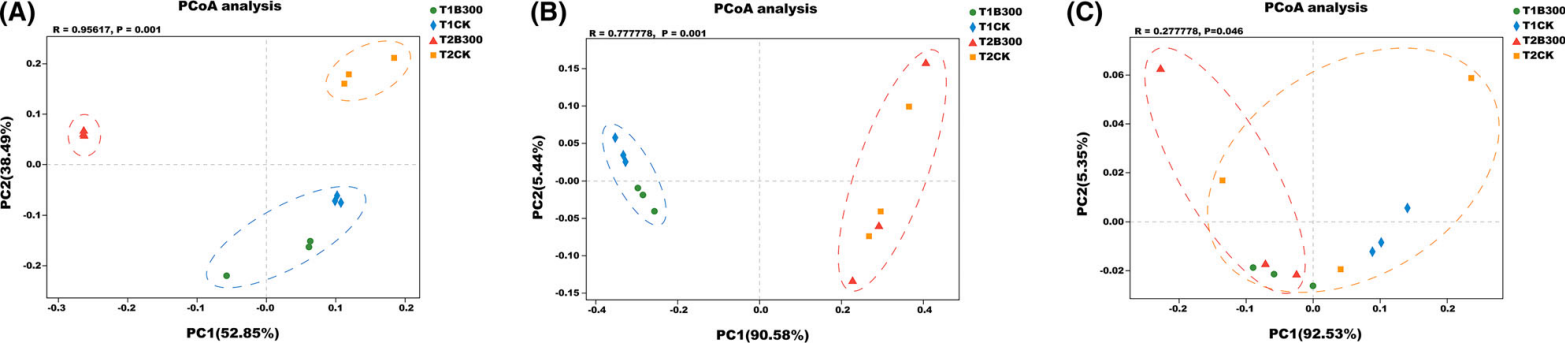
Principal coordinate analysis (PCoA) based on Bray–Curtis dissimilarities of bacterial, fungal and archaeal communities in bulk soil and potato rhizosphere soil samples. A. PCoA of bacterial communities in soil samples. B. PCoA of fungal communities in soil samples. C. PCoA of archaea communities in soil samples. Analysis of similarities (ANOSIM) was used for significance testing. T1CK and T1B300 indicate bulk soil samples collected from the CK and B300 treatment plots, respectively, 3 days prior to planting. T2CK and T2B300 indicate rhizosphere soil samples in the CK and 300 kg ha^−1^ MCB product treatment plots, respectively, collected at the time of early tuber formation.

PCoA was conducted to examine the influence of the MCB product on microbial community structure. Results indicated that bacterial, fungal and archaeal communities of bulk soil samples obtained from the CK and B300 treatment plots (T1CK and T1B300) collected 3 days prior to planting clustered together (Fig. 2A), providing evidence that the microbial community in bulk soil was similar among the different treatment plots. In contrast, the bacterial community in the CK and MCB product treatment plots collected at the time of early tuber formation (T2CK and T2B300) grouped into different clusters (Fig. 2A). The difference between T2CK and T2B300 treatment plots revealed that the soil bacterial community was significantly impacted by the application of the MCB product. A significant dissimilarity in the bacterial community was also observed between the T2CK and T2B300 treatment plots (Fig. 2A). The clustering of fungal and archaeal communities, however, was similar in the T2CK and T2B300 treatment plots (Fig. 2B and C). These results indicate that the bacterial community was more sensitive than other phyla to the application of the MCB product.

**Fig. 3:**
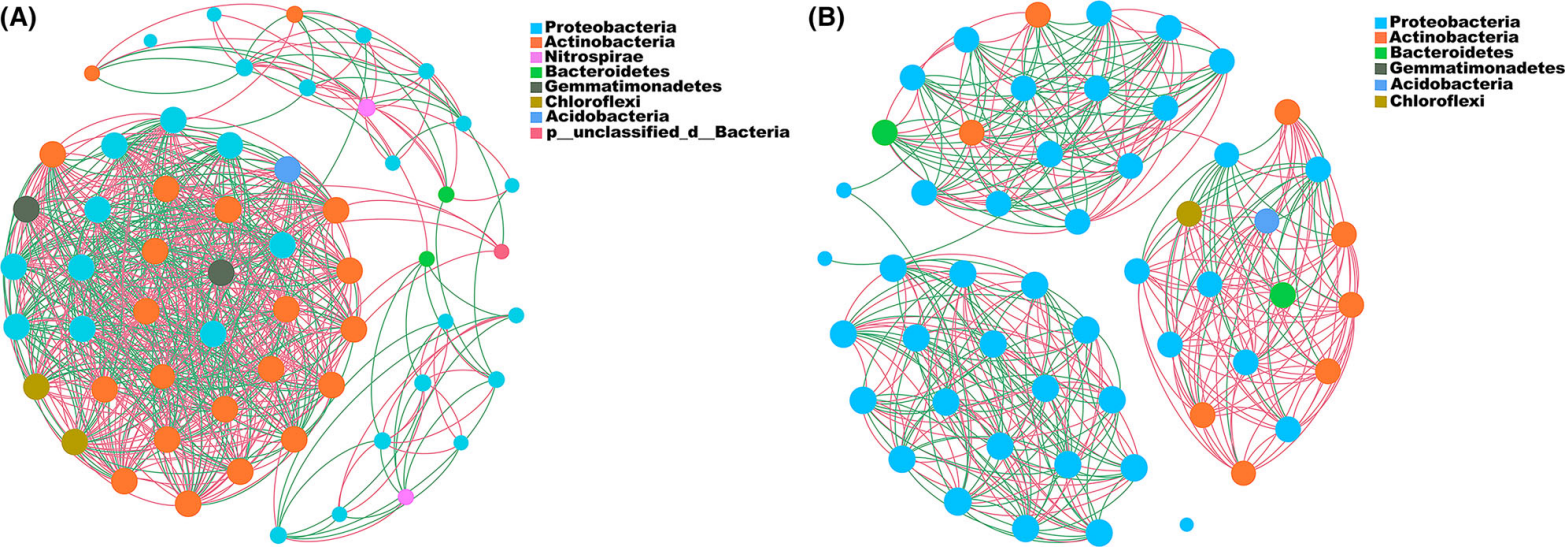
Relationships among taxa in bacterial community of potato rhizosphere soil samples. A. Co‐occurrence networks in datasets obtained from CK plots. B. Co‐occurrence networks in datasets of MCB product treatment plots. Each node represents a bacterial phylotype, whereas the edges represent significant correlations, with the magnitude >+0.85 (positive correlation – red edges) or <–0.85 (negative correlation – green edges) between the nodes. Each node is labelled at the phylum level. The size of each node is proportional to the number of connections.

The co‐occurrence patterns of all networks differed significantly among samples treated with the MCB product. Co‐occurrence network analysis revealed similar node numbers among the bacteria in the bulk soil communities obtained from the MCB product and the CK plots at T1 and T2 (Fig. 3, Fig. S7). The node numbers of Proteobacteria, however, were reduced in the T2CK plots, relative to the bulk soil samples, after potatoes had been planted. In contrast, the node numbers of Proteobacteria increased in the T2B300 treatment plots, relative to the T2CK plots. Collectively, the data provide further evidence that the bacterial community in potato rhizosphere soil was affected by the MCB product treatment, exhibiting a significant increase in the relative abundance of Proteobacteria (Fig. 3, Fig. S7).

### The MCB product induces enrichment in genes encoding nitrogen metabolism enzymes, carbon fixation enzymes and carbon‐degrading enzymes

**Fig. 4:**
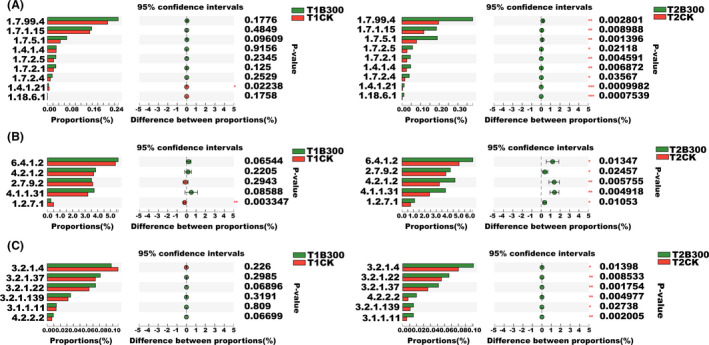
Relative abundance of genes encoding key enzymes involved in nitrogen metabolism, carbon fixation and carbon degradation pathways. A. Genes encoding key enzymes in nitrogen metabolism pathways. B. Genes encoding key enzymes in carbon fixation pathways. C. Genes encoding key enzymes in carbon fixation carbon degradation pathways. *, ** and *** indicate a significant correlation at *P* < 0.05, *P* < 0.01 and *P* < 0.001 respectively. T1CK and T1B300 indicate bulk soil samples obtained from CK and B300 treatment plots, respectively, 3 days prior to planting. T2CK and T2B300 indicate rhizosphere soil samples obtained from untreated (CK) plots and plots treated with 300 kg ha^−1^ of the MCB product, respectively, collected at the time of early tuber formation.

The metagenomic sequence data were used to identify genes encoding key enzymes involved in nitrogen metabolism, carbon fixation and carbon degradation in rhizosphere soils treated with the MCB product. Results indicated that the MCB product treatment had a significant effect on the abundance of genes encoding nitrogen metabolism enzymes, carbon fixation enzymes and carbon‐degrading enzymes in rhizosphere soils compared with the CK rhizosphere soil samples (Fig. 4).

The relative abundances of genes encoding key enzymes involved in nitrogen metabolism, including nitrate reductase (EC: 1.7.99.4), nitrite reductase (NADH) (EC: 1.7.1.15), nitrate reductase (quinone) (EC: 1.7.5.1), nitric oxide reductase (cytochrome c) (EC: 1.7.2.5), nitrite reductase (EC: 1.7.2.1), dehydrogenase (NADP+) (EC: 1.4.1.4), nitrous‐oxide reductase (EC: 1.7.2.4), aspartate dehydrogenase (EC: 1.4.1.21) and nitrogenase (EC: 1.18.6.1) significantly increased in T2B300 samples collected from plots treated with the MCB product compared with T2CK samples. The 6.12‐, 2.60‐, 1.46‐, 1.09‐ and 1.43‐fold increases in the abundance of nitrogenase (EC: 1.18.6.1), aspartate dehydrogenase (EC: 1.4.1.21), nitrous oxide reductase (EC: 1.7.2.4), nitric oxide reductase (cytochrome c) (EC: 1.7.2.5) and nitrate reductase (quinone) (EC: 1.7.5.1) were observed, respectively, in rhizosphere soil samples of plots treated with the MCB product, relative to CK plots. The abundance of nitrite reductase (EC: 1.7.2.1) and nitrate reductase (EC: 1.7.99.4) increased by 1.73‐fold and 1.93‐fold in rhizosphere soils collected from plots that had received the MCB product treatment, relative to CK plots (Fig. 4A). These results indicate that the processes of nitrogen fixation, ammonification, denitrification and assimilatory nitrite reduction pathways were potentially more active in microbial communities in the potato rhizosphere soils collected from plots treated with the MCB product, which may have had a significant impact on nitrogen availability.

Genes encoding key enzymes involved in carbon fixation identified in the dataset were acetyl‐CoA carboxylase (EC: 6.4.1.2), phosphoenolpyruvate synthase (EC: 2.7.9.2), fumarate hydratase (4.2.1.2), phosphoenolpyruvate carboxylase (EC: 4.1.1.31) and pyruvate synthase (EC: 1.2.7.1). The abundance of genes encoding these enzymes was 1.10‐ to 1.58‐fold higher in plots treated with the MCB product than in the rhizosphere soil of CK plots (Fig. 4B). The relative abundances of genes encoding key enzymes involved in carbon degradation that were identified in the dataset included pectin esterase (EC: 3.1.1.11), α‐glucuronidase (EC: 3.2.1.139), α‐galactosidase (EC: 3.2.1.22), xylan 1,4‐β‐xylosidase (EC: 3.2.1.37), cellulase (EC: 3.2.1.4) and pectate lyase (EC: 4.2.2.2). The relative abundance of these genes was 2.70‐, 1.44‐, 1.19‐, 1.43‐ and 1.27‐fold higher, respectively, in rhizosphere soils of plots treated with the MCB product than that in the rhizosphere soil samples of CK plots (Fig. 4C).

### Taxonomic classification of the metagenomic sequences encoding genes associated with nitrogen metabolism, carbon fixation and carbon degradation

**Fig. 5:**
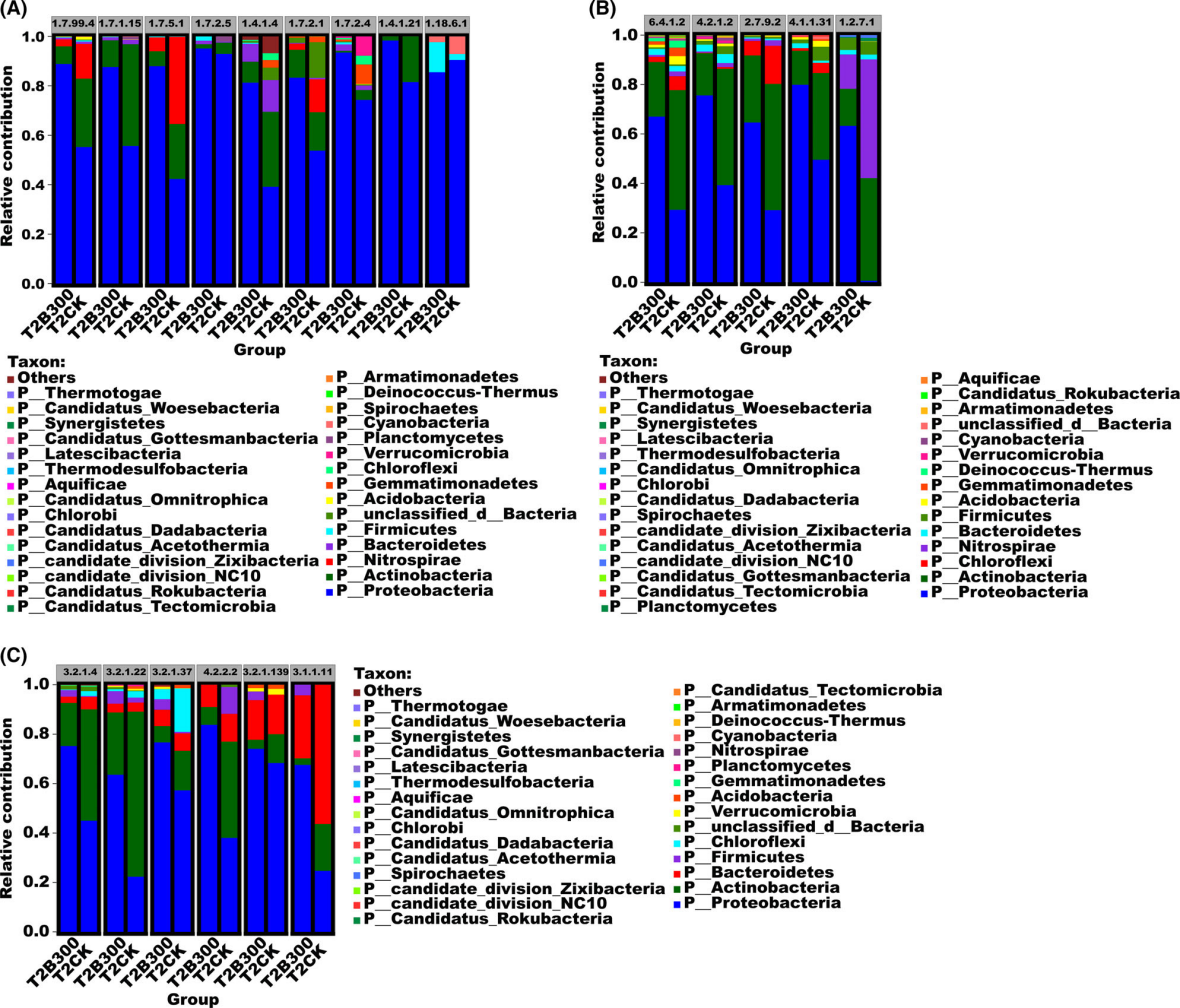
Distribution of bacterial taxa at the phylum level contributing to the relative abundance of genes encoding key enzymes responsible for (A) nitrogen metabolism, (B) carbon fixation and (C) carbon degradation. T2CK and T2B300 indicate rhizosphere soil samples obtained from plots that were untreated (CK) or treated with 300 kg ha^−1^ of the MCB product (T2B300), collected at the time (T2) of early tuber formation.

The involvement of bacterial taxa in nitrogen metabolism, carbon fixation and carbon degradation pathways indicates the diverse functional role of rhizosphere microbial communities (Fig. 5). Bacterial species belonging to the phylum Proteobacteria were the main contributors to the increase in the relative abundance of genes encoding key enzymes involved in nitrogen metabolism, carbon fixation and carbon degradation identified in the T2B300 samples but contributed less to the relative abundance of genes encoding key enzymes in the T2CK samples (Fig. 5). This finding suggests that Proteobacteria present in the T2B300 samples was the main component contributing to the potential metabolic activity of that microbial community.

At the phylum level, an average of 25 (90%) and 343 reads (86%) for nitrogenase genes were assigned to species in Proteobacteria in T2CK and T2B300 samples, respectively (Fig. 5A, Table␣S1). An average of 49 (12%) and 9 (2.3%) of the reads representing nitrogenase genes were assigned to Firmicutes and Cyanobacteria in the T2 samples and B300 samples respectively. In contrast, the abundance of detected nitrogenase genes in taxa belonging to these phyla was extremely low in T2CK samples. Proteobacteria was also the main contributor to the relative abundance of genes encoding aspartate dehydrogenase in T2CK (50 reads) and T2B300 (437 reads) samples (Fig. 5A). Proteobacteria was the principle contributor of nitrous oxide reductase genes, with an average of 222 (74.3%) and 1378 reads (93.5%) in T2CK and T2B300 samples respectively. Bacteroidetes, Actinobacteria, Chloroflexi and Gemmatimonadetes in samples from each of the treatment groups only contributed marginally to the observed relative abundance of nitrous oxide reductase genes. Firmicutes also contributed to the relative abundance of nitrous oxide reductase genes identified in T2B300 samples but were not present in T2CK samples (Fig. 5A). Proteobacteria also contributed a large proportion to the relative abundance of nitrite reductase, dehydrogenase (NADP+), nitrate reductase (quinone), nitrite reductase (NADH) and nitrate reductase soil samples from all treatment groups, especially T2B300 samples. The relative abundance of nitrogenase genes at the level of genus was especially informative in T2B300 samples, with *Azospirillum*, which belongs to order Rhodospirillales, providing a significant contribution overall (Fig. S8, Tables S2 and S3). *Pseudomonas* contributed mostly to the relative abundance of aspartate dehydrogenase genes in T2B300 samples, followed by *Achromobacter*, *Delftia* and *Acinetobacter*. *Pseudomonas*, *Achromobacter*, *Acidovorax*, *Azospirillum* and *Rhizobium* contributed a large proportion to the relative abundance of nitrous oxide reductase genes in T2B300 samples. *Pseudomonas*, *Nocardioides*, *Pseudoxanthomonas*, *Achromobacter* , *Azospirillum* and *Agrobacterium* were the main contributors to the higher relative abundance of nitrite reductase genes observed in T2B300 samples. *Pseudomonas*, *Lelliottia*, *Achromobacter*, *Acidovorax*, *Acidovorax* and *Azospirillum* contributed significantly to the relative abundance of dehydrogenase (NADP+) genes, and *Pseudomonas*, *Pseudoxanthomonas*, *Achromobacter*, *Azospirillum* and *Agrobacterium* contributed significantly to the relative abundance of nitric oxide reductase (cytochrome c) genes identified in T2B300 samples. *Pseudomonas*, *Lelliottia*, *Achromobacter*, *Delftia*, *Acidovorax*, *Lysobacter*, *Vogesella* and *Enterobacter* were the main contributors to the relative abundance of nitrate reductase (quinone) genes observed in T2B300 samples. *Pseudomonas*, *Lelliottia*, *Pseudoxanthomonas*, *Achromobacter*, *Delftia*, *Acidovorax*, *Azospirillum*, *Agrobacterium* and *Vogesella* contributed to the high level of relative abundance of nitrate reductase genes in T2B300 samples, and *Pseudomonas*, *Pseudoxanthomonas*, *Achromobacter*, *Delftia*, *Acidovorax*, *Azospirillum*, *Vogesella*, *Cellvibrio* and *Enterobacter* contributed a major portion of the high relative abundance of nitrite reductase (NADH) genes observed in T2B300 samples relative to T2CK samples (Fig. S8, Table␣S3). The main contributors to the relative abundance of genes encoding key enzymes involved in nitrogen metabolism at the order level are shown in Fig. S8 and Table␣S2.

Proteobacteria was the main contributor to the higher relative abundance of genes encoding enzymes involved in carbon fixation in T2B300 soil samples, including acetyl‐CoA carboxylase, fumarate hydratase, phosphoenolpyruvate synthase, phosphoenolpyruvate carboxylase and pyruvate synthase (Fig. 5B, Table␣S4). *Pseudomonas* and *Lelliottia* were the main contributors, at the genus level, contributed mostly to the higher relative abundance of genes encoding acetyl‐CoA carboxylase, fumarate hydratase, phosphoenolpyruvate synthase and phosphoenolpyruvate carboxylase. *Lelliottia* was only found in T2B300 samples (Fig. S8, Table␣S6) and was the principle contributor to the relative abundance of pyruvate synthase genes. The main contributors to the relative abundance of genes encoding enzymes involved in carbon fixation at the order level are shown in Fig. S8 and Table␣S5.

Several genes encoding enzymes involved in carbon degradation were also attributed to taxa in the phylum Proteobacteria in T2B300 samples, where the relative abundance was significantly higher than that in T2CK samples (Fig. 5C, Table␣S7). *Lelliottia* was also the principle contributor, at the genus level, to the higher relative abundance of genes encoding cellulase, α‐galactosidase, xylan 1,4‐β‐xylosidase, pectate lyase and pectin esterase, and *Pseudoxanthomonas* was the main contributor to the relative abundance of genes encoding α‐glucuronidase (Fig. S8, Table␣S9). The most dominant order of taxa encoding these key enzymes is shown in Fig. S8 and Table␣S8. Collectively, the results indicate that a higher number and relative abundance of genes encoding enzymes involved in nitrogen metabolism, carbon fixation and carbon degradation pathways were present in T2B300 samples. The MCB product treatment also significantly affected the taxonomic composition of rhizosphere bacteria associated with nitrogen metabolism, carbon fixation and carbon degradation. The abundance of genes encoding these key enzymes was directly linked to rhizosphere bacteria that were significantly affected by the MCB product treatment. The data suggest that the application of the MCB product may have significantly promoted nitrogen metabolism, carbon fixation and carbon degradation.

## Discussion

The monoculture production of potato routinely results in a significant decline in yield over time. Our results indicate, however, that the application of 300 kg ha^−1^ of an MCB product significantly increased potato tuber yield by 63.58%, relative to untreated plots, in fields that were continuously cropped with potatoes. Only a few bioagents have been demonstrated to improve potato tuber yield at a similar level under field conditions. This can perhaps be attributed to the impact of environmental stress on the bioactive agents. Seed tubers treated with *Pseudomonas fluorescens LBUM223*, *Burkholderia ambifaria* and *Azospirillum lipoferum* AL‐3 significantly increased total tuber weight by 46%, 11–15% and 16.1–22.7% respectively (Arseneault *et␣al*., 2015; Larkin, 2016; Mehmood *et␣al*., 2021). In our study, total nitrogen and soil organic carbon were higher in MCB product‐treated plots than those in untreated plots. Potatoes have a high nitrogen demand during tuber expansion, and tuber yield has been reported to increase with higher total available soil nitrogen (Ojala *et␣al*., 1990).

Importantly, the product that was utilized in the present study, in addition to the microbial agents (*Bacillus subtilis* and *Trichoderma harzianum*), also contained diatomaceous earth as a carrier, which can also have direct impacts on the soil that are beneficial to plant growth (Gokavi *et␣al*., 2020). The application of diatomaceous earth has been reported to decrease tuber damage caused by insects and significantly increase the plant height and stem diameter without affecting potato productivity (De Assis *et␣al*., 2012). Diatomaceous earth can be applied to the soil in combination with soil amendments to increase drought and insect resistance in plants and can also serve as a bioactive carrier for a microbial consortium. Notably, diatomaceous earth has been utilized in commercial formulations of *Bacillus* spp. and other biocontrol agents (Schisler *et␣al*., 2004). The focus of the present study was to examine the impact of the MCB product on the composition of the rhizosphere and soil micrbiome, as well as the impact that the potential shift might have had on various soil processes, including nitrogen and carbon metabolism. The objective was to provide insights into the potential mechanisms associated with the improved potato yield that occurs in response to the application of the MCB product.

Distinct differences in taxonomic composition at the level of phylum, order and genus were observed in the␣soil bacterial community between MCB product and the untreated plots. Proteobacteria was the most dominant phylum in all of the soil samples, which is consistent with previous studies on the microbiota of potato soils (Rosenzweig *et␣al*., 2012; Tomihama *et␣al*., 2016) as well as our previous study (Wang *et␣al*., 2019a). Notably, the MCB product applied at 300 kg ha^−1^ significantly increased the relative abundance of Proteobacteria in the rhizosphere of potato. Proteobacteria ranked high in the co‐occurrence network of rhizosphere soil in MCB product treatment plots, and the interaction between Proteobacteria and other bacteria in the rhizosphere bacterial network increased and became more and more complex, indicating that Proteobacteria taxa were important in the microbiota of plots treated with the MCB product. Proteobacteria has been reported to harbour a large group of metabolic enzymes, which have a strong influence on global nitrogen and carbon cycles, as well as soil metabolites due to their great metabolic diversity (Spain *et␣al*., 2009; Pan *et␣al*., 2020). Prevalent organic substrates, such as amino acids, fatty acids and urea, present in rhizosphere soils were reported to be mainly due to the activity of populations of Proteobacteria (Pan *et␣al*., 2020). The high level of relative abundance of Proteobacteria observed was directly associated with increased potato tuber yield. The increased interaction between Proteobacteria and other bacteria in the rhizosphere bacterial network potentially made available to plants increased levels of organic substrate nutrients that support plant growth. The results of microbial composition and co‐occurrence network analyses indicate that Proteobacteria may play an important role in potato growth. Gumiere *et␣al*. (2019) also reported that the functional activity and interactions of soil bacteria have potential relevance to potato yields.

**Fig. 6:**
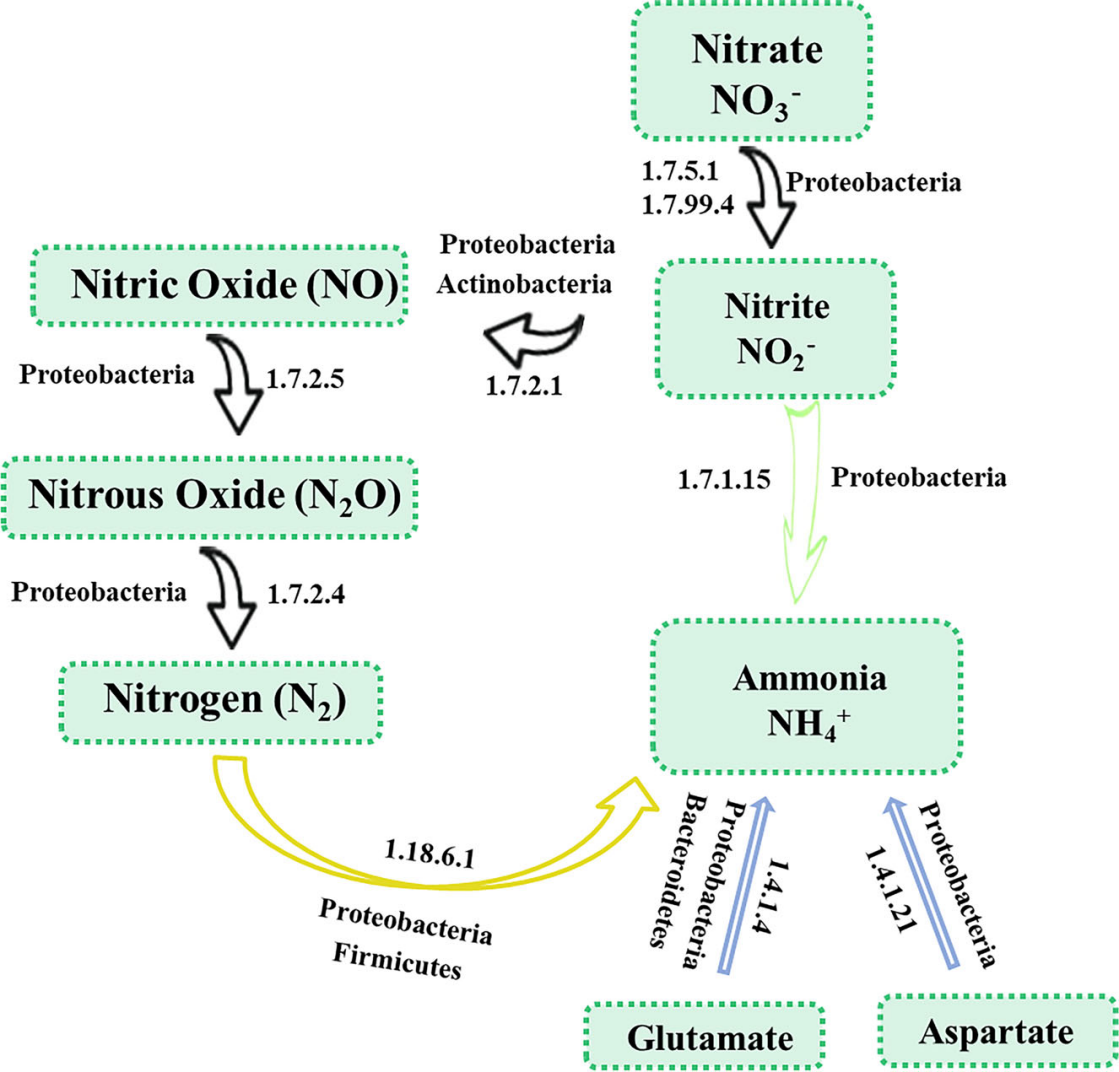
Pathways involved in nitrogen metabolism in rhizosphere bacterial taxa that were recruited and proliferated in samples obtained from plots treated with the MCB product. The impacted processes include denitrification (black), ammonification (blue), dissimilatory nitrite reduction (green) and nitrogen fixation (yellow). Phyla of the taxa that contribute to these processes are indicated.

Significant differences in the potential activity of the microbial community were observed in the MCB product‐treated and CK plots. A variety of genes encoding key enzymes associated with processes related to nitrogen metabolism, carbon fixation and carbon degradation were significantly enriched in the rhizosphere soils of MCB product‐treated plots, relative to CK plots. This suggests that there is strong potential for soil nutrient processing and transport to occur in potato rhizosphere soils treated with the MCB product. The highest abundance of genes encoding enzymes related to nitrogen metabolism was observed in the MCB product treatment plots, especially nitrogenase, aspartate dehydrogenase, nitrous oxide reductase, nitric oxide reductase (cytochrome c) and nitrate reductase (quinone). Proteobacteria was the principal contributor to the relative abundance of genes encoding key enzymes involved in nitrogen metabolism in the rhizosphere soils of MCB product‐treated plots. Our results revealed that the application of the MCB product resulted in the recruitment and proliferation of a high relative abundance of *Azospirillum* in rhizosphere soils, potentially increasing the level of available nitrogenase, which facilitates the reduction of nitrogen gas (N_2_) to ammonia (${\text{NH}}_{4}^{+}$) (Liu *et␣al*., 2019). The anion ${\text{NO}}_{3}^{‐}$ and cation ${\text{NH}}_{4}^{+}$ are absorbable forms of nitrogen for plants and are often limited in agricultural soils (Below, 2002; Zhong *et␣al*., 2014; Kuypers *et␣al*., 2018). Several genes encoding enzymes involved in nitrogen metabolic pathways were highly enriched in the rhizosphere bacterial taxa belonging to Proteobacteria in plots treated with the MCB product. The genes encoded key enzymes associated with nitrogen fixation, ammonification, denitrification and assimilatory nitrite reduction (Fig. 6). Proteobacteria and Bacteroidetes play an essential role in nitrogen metabolism (Kuypers *et␣al*., 2018). The phyla Proteobacteria and Firmicutes have been reported to contain taxa that are the principal contributors of all the genes involved in the nitrogen fixation pathway (Ren *et␣al*., 2018; Wang *et␣al*., 2019b), which is consistent with the results of the present study. Biological nitrogen fixation is a particularly important process in agricultural ecosystems as it decreases the need for the application of external N fertilizer, whose use can lead to environmental issues. Bacteria belonging to the Proteobacteria are central players in nitrogen fixation (Wang *et␣al*., 2019b). The addition of the MCB product significantly increased the abundance of genes encoding nitrogenase (EC: 1.18.6.1), an enzyme that accelerates the reduction of N_2_ to ${\text{NH}}_{4}^{+}$, by more than six‐fold. The results indicate that the bacterial communities in the MCB product treatment plots have a higher potential for nitrogen fixation. This is consistent with the higher abundance of Proteobacteria in T2B300 soils. Proteobacteria taxa are also associated with nitrate and nitrite ammonification (Rampadarath *et␣al*., 2018), and the relative abundance of Proteobacteria has been reported to be closely related to denitrification (Srinandan *et␣al*., 2011). Denitrification is responsible for the biological production of NO, N_2_O and N_2_ from ${\text{NO}}_{3}^{‐}$. The dissimilatory nitrite reduction process accelerated by dissimilatory nitrite reductase helps consume ${\text{NO}}_{2}^{‐}$, promoting the accumulation of ${\text{NH}}_{4}^{+}$. Collectively, the data suggest that the processes of nitrogen fixation, ammonification, denitrification and assimilatory nitrite reduction pathways were potentially more active in the microbial communities of MCB product treatment plots, which appears to have had a positive impact on nitrogen metabolism in the treated plots.

Results of our study indicate that genes encoding enzymes involved in pyruvate metabolism and the citric acid (TCA) cycle and associated with taxa in Proteobacteria were highly represented. This result is consistent with the study of Salam (2018), who reported that genes present in the soil metagenome that were annotated for pyruvate metabolism and the citric acid (TCA) cycle were largely contributed by taxa in Proteobacteria. In addition, many genes encoding enzymes involved in pectin degradation, hemicellulose degradation and cellulose degradation were more abundant in MCB product treatment samples. The degradation of pectin and hemicellulose is carried out by pectinolytic soil bacteria, including taxa in the Proteobacteria, *Arthrobacter* (Actinobacteria) and *Bacillus* (Firmicutes). Cellulose is decomposed by cellulases which can be produced by soil bacteria belong to Actinobacteria, Proteobacteria and Firmicutes (Horwath, 2017). Proteobacteria may play an important role in accelerating carbon degradation, resulting in an accumulation of soil organic carbon derived from the decomposition of plant residues. Priya *et␣al*. (2018) reported that the enrichment of carbohydrate metabolism in soils may be linked to a higher content of total organic carbon in soils. In the present study, carbon degradation and carbon fixation processes may have been enriched by the rhizosphere bacterial community, which is consistent with higher content of soil organic carbon present in the plots treated with the MCB product.

## Conclusions

The application of an MCB product containing a consortium of *Bacillus subtilis* and *Trichoderma harzianum*, and diatomaceous earth as a carrier, increased potato tuber yield and significantly altered the structure of the bacterial rhizosphere community by fostering the recruitment and proliferation of beneficial bacteria, especially taxa in Proteobacteria with nitrogen‐transforming capacity. The functional potential of the potato rhizosphere bacterial community was enriched in taxa with genes encoding enzymes associated with nitrogen and carbon metabolism, as well as carbon degradation. The degree to which the alterations in the microbial community were due to the microbial consortium component of the product, the diatomaceous earth, or a combination of both components acting additively or synergistically, will require further study. The present research provides data that can be used to further develop biological approaches for improving potato tuber yield.

## Experimental procedures

### Experimental design and soil sampling

The study was conducted in Guyang County (40^o^58′N, 109^o^53′E), Baotou city, Inner Mongolia, China, a major potato production region. The soil type was sandy soil. Potatoes had been continuously grown in this field for 8 years. A commercial MCB product, Micro‐Ecological Agents^®^ (Sino Green Agri‐Biotech Co. Ltd., Beijing, China) was used in this study. The MCB product is composed of a consortium of *Bacillus subtilis* (strain znjdf1), which is deposited in the China General Microbiological Culture Collection Center (CGMCC) (Accession NO. 7850), and *Trichoderma harzianum* (strain znlkhc1), which is also deposited in the CGMCC (Accession NO.7861), and diatomaceous earth as a carrier for the microbial components. The concentration of *B. subtilis* and *T. harzianum* in the final preparation of the MCB product is 9.5 × 10^8^ and 0.5 × 10^8^ CFU g^−1^ respectively (Wang *et␣al*., 2019a). A concentration of 300 (B300) kg ha^−1^ MCB product was used in this study, and untreated blocks served as a control. A randomized complete block design was used to designate treated and untreated plots. Three blocks (replicates) were designated for each treatment, with each block measuring 23 m × 24 m.

Potato (*Solanum tuberosum* L. cv. Kexin No.1) seed tubers were planted in furrows. There was about a 20 cm interval between each seed tuber and approximately a 70 cm distance between each furrow. The MCB product (300 kg ha^−1^) was added to the furrows around the potato seed tubers once a year for 3 years (2016, 2017 and 2018). All fields were managed using the local standard agricultural management system used to grow potatoes without the use of synthetic agrochemicals. Potato yield and soil samples from 2018 were analysed in the present study. Bulk soil samples were obtained from each treatment block 3 days prior to planting (T1). Rhizosphere samples were obtained at the time of early tuber formation (T2). The root system of a collected plant was vigorously shaken to detach loosely adhering soil, and the thin layer of rhizosphere soil was separated and collected using a small brush and placed in a sterile plastic bag (Wang *et␣al*., 2019a). Each soil sample comprised pooled rhizosphere soil obtained from 15 plants within a single block. There were three blocks (replicates) for either the control (CK) or treatment group. All soil samples were divided into two subsamples. One subsample was stored at −80°C for subsequent DNA extraction, and the other subsample was used for the analysis of the physicochemical soil properties.

### Determination of potato yield and physicochemical soil properties

The potatoes in each block were manually harvested and weighed to determine yield. Soil organic carbon (SOC) and total nitrogen (TN) in the bulk and rhizosphere soil samples were quantified via dichromate oxidation (Nelson and Sommers, 1982) and the Kjeldahl method (Bremner and Mulvaney, 1982). Each soil sample comprised pooled rhizosphere soil obtained from 15 plants within a single block. There were three blocks (replicates) for either the CK or treatment group.

### DNA extraction, library construction and metagenomic sequencing

Total genomic DNA was extracted from 0.5 g soil samples using an MP DNA Isolation Kit (MO BIO Laboratories, Carlsbad, CA, USA) according to the manufacturer’s instructions. The quality, concentration and purity of extracted DNA were determined using a 1% agarose gel, TBS‐380 and NanoDrop 2000 respectively. The DNA extracts were fragmented to obtain an average size of approximately 300 bp using a Covaris M220 (Gene Company Limited, Shanghai, China) for use in paired‐end library construction. Paired‐end libraries were constructed using NEXTFLEX^®^ Rapid DNA‐Seq (Bio Scientific, Austin, TX, USA). Adapters containing the full complement of sequencing primer hybridization sites were ligated to the blunt end of fragments. Paired‐end sequencing was performed on an Illumina NovaSeq (Illumina Inc., San Diego, CA, USA) platform at Majorbio Bio‐Pharm Technology Co., Ltd. (Shanghai, China) using NovaSeq Reagent Kits according to the manufacturer’s instructions (www.illumina.com). Sequence data associated with this project have been deposited in the NCBI Short Read Archive database (Accession Number: SRP288137).

### Sequence quality control and genome assembly

The obtained sequences were processed and analysed on the free, online Majorbio Cloud Platform (www.majorbio.com). The paired‐end Illumina reads were trimmed to remove adaptors, and low‐quality reads (length < 50 bp or with a quality value < 20 or having N bases) were also removed using fastp (Chen *et␣al*., 2018) (https://github.com/OpenGene/fastp, version 0.20.0).

Reads were aligned to the *Solanum tuberosum* genome sequence using BWA (Li and Durbin, 2009) (http://bio‐bwa.sourceforge.net, version 0.7.9), and any sequences exhibiting homology to the potato genome and their mated reads were removed.

Metagenomic data were assembled using MEGAHIT (Li *et␣al*., 2015) (https://github.com/voutcn/megahit, version 1.1.2), which makes use of succinct de Bruijn graphs. Contigs with a length ≥ 300 bp were selected as the final assembly. The resulting contigs were then used for gene prediction and annotation.

### Gene prediction, taxonomy and functional annotation

Open reading frames (ORFs) from each assembled contig were predicted using MetaGene (Noguchi *et␣al*., 2006) (http://metagene.cb.k.u‐tokyo.ac.jp/). The predicted ORFs ≥ 100 bp in length were retrieved and translated into amino acid sequences using the NCBI translation table (http://www.ncbi.nlm.nih.gov/Taxonomy/taxonomyhome.html/index.cgi?chapter=tgencodes#SG1).

A non‐redundant gene catalogue was constructed using CD‐HIT (Fu *et␣al*., 2012) (http://www.bioinformatics.org/cd‐hit/, version 4.6.1) with the parameters of 90% sequence identity and 90% coverage. Reads, after quality control, were mapped to the non‐redundant gene catalogue using the criterion of 95% identity with SOAPaligner (Li *et␣al*., 2008) (http://soap.genomics.org.cn/, version 2.21), and gene abundance in each sample was assessed.

Representative sequences of the non‐redundant gene catalogue were aligned to the NCBI NR database using an e‐value cut‐off of 1e^‐5^ with Diamond (Buchfink *et␣al*., 2015) (http://www.diamondsearch.org/index.php, version 0.8.35) for taxonomic annotations. KEGG annotation was also conducted using Diamond (Buchfink *et␣al*., 2015) (http://www.diamondsearch.org/index.php, version 0.8.35) against the Kyoto Encyclopedia of Genes and Genomes database (http://www.genome.jp/keeg/) with an e‐value cut‐off of 1e^‐5^.

### Statistical analysis

Potato yield, soil organic carbon and total nitrogen in the CK and B300 soil samples were compared by determining the least significant difference at a probability level of 0.05 using the one‐way ANOVA within the R program (version 3.1.2) (http://www.r‐project.org/). STAMP software was used for the analysis of the relative abundances of taxa and function in each sample metagenome (Parks *et␣al*., 2014). Principal coordinate analysis (PCoA) was performed to examine dissimilarities in soil microbiome composition in QIIME2 2019.4.0 based on unweighted UniFrac distance (Lozupone *et␣al*., 2011). Network analysis was used to characterize bacterial modules using Gephi (version 0.9.1) software (Bastian *et␣al*., 2009). The taxonomic information for each selected read (at phylum, order and genus levels) was extracted, and the relationships between them were calculated using the method described by Ofek‐Lalzar *et␣al*. (2014) to explore the relative contribution of taxa to the rhizosphere and to rhizoplane‐enriched functional genes. UPGMA cluster analysis using the Bray–Curtis distance was performed to compare community compositions in the different treatment groups based on the relative abundance of taxa. All statistical analyses and plots were conducted within the R program (version 3.1.2) (http://www.r‐project.org/).

## Funding Information

This work was supported by China Postdoctoral Science Foundation (2020M680770) and National Key R&D Program of China (2019YFD1002003) and National Key R&D Program of China (2017YFD0201100).

## Conflicts of interest

The authors declare no conflict of interest.

## Supporting information

**Fig.␣S1**. Tuber yield (A), soil organic carbon (B) and total nitrogen (C) in untreated (CK) plots and plots treated with 300 (B300) kg ha^−1^ of the MCB product containing a consortium of *Bacillus subtilis* and *Trichoderma harzianum*. T1CK and T1B300 indicate bulk soil samples collected from CK and B300 blocks, respectively, 3 days prior to planting. T2CK and T2B300 indicate rhizosphere soil samples collected from CK and B300 blocks, respectively, at the time of early tuber formation. Data represent the mean ± standard deviation (*n* = 3). Significant differences between treatments and the control were determined by ANOVA. Significantly different means (*P* < 0.05) are indicated by different letters above each bar.
**Fig.␣S2**. Comparative analysis of dominant bacterial taxa in bulk and potato rhizosphere soil samples collected from untreated (CK) and MCB product (300 kg ha^−1^) treatment plots. (A) Relative abundance of the most abundant bacterial orders. (B) Bacterial orders with different relative abundance. Taxonomic profile of bacterial orders whose abundance was significantly different between CK plots and plots treated with MCB product (300 kg ha^−1^) 3 days prior to planting (T1, upper panel) and early tuber formation (T2, lower panel). (C) Relative abundance of the most abundant bacterial genera. (D) Bacterial genera with different relative abundance. Taxonomic profile of bacterial genera whose abundance was significantly different between CK plots and plots treated with MCB product (300 kg ha^−1^) 3 days prior to planting (T1, upper panel) and early tuber formation (T2, lower panel). *, ** and *** indicate a significant correlation at *P* < 0.05, *P* < 0.01 and *P* < 0.001 respectively. T1CK and T1B300 indicate bulk soil samples collected from CK and B300 blocks, respectively, 3 days prior to planting. T2CK and T2B300 indicate rhizosphere soil samples collected from untreated and MCB product (300 kg ha^−1^) treatment plots, respectively, at the time of early tuber formation.
**Fig.␣S3**. An unweighted paired group method with arithmetic mean (UPGMA) tree analysis of bacterial community composition. The UPGMA tree was constructed based on “Bray–Curtis” distances between bacterial communities in each treatment. T1CK and T1B300 indicate bulk soil samples collected from untreated (CK) and MCB product (300 kg ha^−1^) treatment plots, respectively, 3 days prior to planting. T2CK and T2B300 indicate rhizosphere soil samples collected from CK and B300 (300 kg ha^−1^ MCB) plots, respectively, at the time of early tuber formation.
**Fig.␣S4**. Venn diagrams indicating the number of bacterial genera (A), fungal genera (B) and archaeal genera (C) in bulk and potato rhizosphere soil samples collected from untreated plots and plots treated with the MCB product. T1CK and T1B300 indicate bulk soil samples collected from CK and B300 plots, respectively, 3 days prior to planting. T2CK and T2B300 indicate rhizosphere soil samples collected from plots treated with 0 and 300 kg ha^−1^ MCB product, respectively, at the time of early tuber formation.
**Fig.␣S5**. Comparative analysis of the dominant fungal communities in bulk and potato rhizosphere soil samples collected from untreated (CK) and MCB product (300 kg ha^−1^) treatment plots respectively. (A) Relative abundance of the most abundant fungal orders. (B) Fungal orders with different relative abundance. Taxonomic profile of fungal orders with a significantly different relative abundance between 300 kg ha^−1^ MCB product treatment and CK plots 3 days prior to planting (T1, upper panel) and early tuber formation (T2, lower panel). (C) Relative abundance of the most abundant fungal genera. (D) Fungal genera with different relative abundance. Taxonomic profile of fungal genera with a significant difference between 300 kg ha^−1^ MCB product treatment and CK plots 3 days prior to planting (T1, upper panel) and early tuber formation (T2, lower panel). *, ** and *** indicate a significant correlation at *P* < 0.05, *P* < 0.01 and *P* < 0.001 respectively. T1CK and T1B300 indicate bulk soil samples collected from CK and B300 plots, respectively, 3 days prior to planting. T2CK and T2B300 indicate rhizosphere soil samples collected from untreated (CK) and MCB product (300 kg ha^−1^) treatment plots, respectively, at the time of early tuber formation.
**Fig.␣S6**. Comparative analysis of dominant archaeal communities in bulk and potato rhizosphere soil samples collected from untreated (CK) and MCB product (300 kg ha^−1^) treatment plots. (A) Relative abundance of the most abundant archaeal orders. (B) Archaeal orders with different relative abundance. Taxonomic profile of archaeal orders with significantly different relative abundance between the 300 kg ha^−1^ MCB product treatment and CK plots 3 days prior to planting (T1, upper panel) and early tuber formation (T2, lower panel). (C) Relative abundance of the most abundant archaeal genera. (D) Archaeal genera with different relative abundance. Taxonomic profile of archaeal genera with a significant difference between 300 kg ha^−1^ MCB product treatment and CK plots 3 days prior to planting (T1, upper panel) and early tuber formation (T2, lower panel). *, ** and *** indicate a significant correlation at *P* < 0.05, *P* < 0.01 and *P* < 0.001 respectively. T1CK and T1B300 indicate bulk soil samples collected from CK and B300 plots, respectively, 3 days prior to planting. T2CK and T2B300 indicate rhizosphere soil samples collected from untreated (CK) and MCB product (300 kg ha^−1^) treatment plots, respectively, at the time of early tuber formation.
**Fig.␣S7**. Relationships among reads in the taxa of the bacterial community in potato bulk soil samples. (A) Co‐occurrence networks in datasets obtained from CK plots. (B) Co‐occurrence networks in datasets obtained from MCB product (300 kg ha^−1^) treatment plots. Each node represents a bacterial phylotype, whereas the edges represent significant correlations, with a magnitude >+ 0.85 (positive correlation‐red edges) or <– 0.85 (negative correlation‐blue edges) between the nodes. Each node is labelled at the phylum level. The size of each node is proportional to the number of connections.
**Fig.␣S8**. Distribution of bacterial taxa contributing to the significantly increased abundance of genes encoding key enzymes in nitrogen metabolism pathways at the order level (A) and genera level (B); in carbon fixation pathways at the order level (C) and genera level (D); in carbon degradation pathways at order level (E) and genera level (F). T2CK and T2B300 indicate rhizosphere soil samples collected from untreated (CK) plots and MCB product (300 kg ha^−1^) treatment plots, respectively, at the time of early tuber formation.
**Table␣S1.** Distribution of bacteria communities at phyla level contributed to the significantly increased abundance of key enzymes in nitrogen metabolic pathways.
**Table␣S2.** Distribution of bacteria communities at order level contributed to the significantly increased abundance of key enzymes in nitrogen metabolic pathways.
**Table␣S3.** Distribution of bacteria communities at genera level contributed to the significantly increased abundance of key enzymes in nitrogen metabolic pathways.
**Table␣S4.** Distribution of bacteria communities at phyla level contributed to the significantly increased abundance of key enzymes in carbon fixation pathways.
**Table␣S5.** Distribution of bacteria communities at order level contributed to the significantly increased abundance of key enzymes in carbon fixation pathways.
**Table␣S6.** Distribution of bacteria communities at genera level contributed to the significantly increased abundance of key enzymes in carbon fixation pathways.
**Table␣S7.** Distribution of bacteria communities at phyla level contributed to the significantly increased abundance of key enzymes in carbon degradation pathways.
**Table␣S8.** Distribution of bacteria communities at order level contributed to the significantly increased abundance of key enzymes in carbon degradation pathways.
**Table␣S9.** Distribution of bacteria communities at genera level contributed to the significantly increased abundance of key enzymes in carbon degradation pathways.
