# Supplementary material for: A microbial consortium‐based product promotes potato yield by recruiting rhizosphere bacteria involved in nitrogen and carbon metabolisms
Source: Microb Biotechnol. 2021 Jul 7;14(5):1961–75. doi: 10.1111/1751-7915.13876 (PMC8449676; doi:10.1111/1751-7915.13876)
Supplement: Supplementary file 1 — Fig.␣S1. Tuber yield (A), soil organic carbon (B) and total nitrogen (C) in untreated (CK) plots and plots treated with 300 (B300) kg ha−1 of the MCB product containing a consortium of Bacillus subtilis and Trichoderma harzianum. T1CK and T1B300 indicate bulk soil samples collected from CK and B300 blocks, respectively, 3 days prior to planting. T2CK and T2B300 indicate rhizosphere soil samples collected from CK and B300 blocks, respectively, at the time of early tuber formation. Data represent the mean ± standard deviation (n = 3). Significant differences between treatments and the control were determined by ANOVA. Significantly different means (P < 0.05) are indicated by different letters above each bar. Fig.␣S2. Comparative analysis of dominant bacterial taxa in bulk and potato rhizosphere soil samples collected from untreated (CK) and MCB product (300 kg ha−1) treatment plots. (A) Relative abundance of the most abundant bacterial orders. (B) Bacterial orders with different relative abundance. Taxonomic profile of bacterial orders whose abundance was significantly different between CK plots and plots treated with MCB product (300 kg ha−1) 3 days prior to planting (T1, upper panel) and early tuber formation (T2, lower panel). (C) Relative abundance of the most abundant bacterial genera. (D) Bacterial genera with different relative abundance. Taxonomic profile of bacterial genera whose abundance was significantly different between CK plots and plots treated with MCB product (300 kg ha−1) 3 days prior to planting (T1, upper panel) and early tuber formation (T2, lower panel). *, ** and *** indicate a significant correlation at P < 0.05, P < 0.01 and P < 0.001 respectively. T1CK and T1B300 indicate bulk soil samples collected from CK and B300 blocks, respectively, 3 days prior to planting. T2CK and T2B300 indicate rhizosphere soil samples collected from untreated and MCB product (300 kg ha−1) treatment plots, respectively, at the time of early tuber formation. Fig.␣S3 [file MBT2-14-1961-s001.zip › mbt213876-sup-0001-supinfo/mbt213876-sup-0009-TableS1-S9.docx]

**Microbial Biotechnology**

| Table S1. Distribution of bacteria communities at phyla level contributed to the significantly increased abundance of key enzymes in nitrogen metabolic pathways. | | | | | | | |
| --- | --- | --- | --- | --- | --- | --- | --- |
| Function | Taxon | T2Ck_a | T2Ck_b | T2Ck_c | T2B300_a | T2B300_b | T2B300_c |
| 1.7.99.4 | p__Proteobacteria | 2634 | 2214 | 1204 | 12058 | 13360 | 12618 |
| 1.7.99.4 | p__Actinobacteria | 1006 | 886 | 1140 | 950 | 1098 | 1008 |
| 1.7.99.4 | p__Bacteroidetes | 34 | 40 | 24 | 110 | 132 | 62 |
| 1.7.99.4 | p__Chloroflexi | 0 | 0 | 0 | 0 | 0 | 0 |
| 1.7.99.4 | p__Firmicutes | 8 | 50 | 6 | 28 | 34 | 40 |
| 1.7.99.4 | p__Acidobacteria | 30 | 46 | 52 | 28 | 34 | 26 |
| 1.7.99.4 | p__Gemmatimonadetes | 0 | 0 | 0 | 0 | 0 | 0 |
| 1.7.99.4 | p__Nitrospirae | 502 | 546 | 494 | 436 | 416 | 360 |
| 1.7.99.4 | p__unclassified_d__Bacteria | 6 | 12 | 10 | 0 | 2 | 0 |
| 1.7.99.4 | p__Cyanobacteria | 0 | 0 | 0 | 0 | 0 | 0 |
| 1.7.99.4 | p__Planctomycetes | 2 | 0 | 0 | 0 | 2 | 0 |
| 1.7.99.4 | p__Deinococcus-Thermus | 0 | 0 | 0 | 0 | 0 | 0 |
| 1.7.99.4 | p__Verrucomicrobia | 6 | 4 | 0 | 4 | 2 | 6 |
| 1.7.99.4 | p__Armatimonadetes | 0 | 0 | 0 | 0 | 0 | 0 |
| 1.7.99.4 | p__Candidatus_Tectomicrobia | 0 | 0 | 0 | 0 | 0 | 0 |
| 1.7.99.4 | p__Candidatus_Rokubacteria | 0 | 0 | 0 | 0 | 0 | 0 |
| 1.7.99.4 | p__candidate_division_NC10 | 0 | 0 | 0 | 0 | 0 | 0 |
| 1.7.99.4 | p__candidate_division_Zixibacteria | 0 | 0 | 0 | 0 | 0 | 0 |
| 1.7.99.4 | p__Spirochaetes | 0 | 0 | 0 | 0 | 0 | 0 |
| 1.7.99.4 | p__Candidatus_Acetothermia | 0 | 0 | 0 | 0 | 0 | 0 |
| 1.7.99.4 | p__Candidatus_Dadabacteria | 0 | 0 | 0 | 0 | 0 | 0 |
| 1.7.99.4 | p__Chlorobi | 0 | 0 | 0 | 0 | 0 | 0 |
| 1.7.99.4 | p__Candidatus_Omnitrophica | 0 | 0 | 0 | 0 | 0 | 0 |
| 1.7.99.4 | p__Aquificae | 0 | 0 | 0 | 0 | 0 | 0 |
| 1.7.99.4 | p__Thermodesulfobacteria | 0 | 0 | 0 | 0 | 0 | 0 |
| 1.7.99.4 | p__Latescibacteria | 0 | 0 | 0 | 0 | 0 | 0 |
| 1.7.99.4 | p__Candidatus_Gottesmanbacteria | 0 | 0 | 0 | 0 | 0 | 0 |
| 1.7.99.4 | p__Synergistetes | 0 | 0 | 0 | 0 | 0 | 0 |
| 1.7.99.4 | p__Candidatus_Woesebacteria | 0 | 0 | 0 | 0 | 0 | 0 |
| 1.7.99.4 | p__Thermotogae | 0 | 0 | 0 | 0 | 0 | 0 |
| 1.7.99.4 | others | 0 | 0 | 0 | 0 | 0 | 0 |
| 1.7.1.15 | p__Proteobacteria | 1510 | 1316 | 812 | 5954 | 6414 | 5946 |
| 1.7.1.15 | p__Actinobacteria | 888 | 844 | 956 | 714 | 860 | 686 |
| 1.7.1.15 | p__Bacteroidetes | 40 | 50 | 28 | 156 | 72 | 30 |
| 1.7.1.15 | p__Chloroflexi | 0 | 0 | 0 | 0 | 0 | 0 |
| 1.7.1.15 | p__Firmicutes | 0 | 0 | 0 | 12 | 18 | 22 |
| 1.7.1.15 | p__Acidobacteria | 4 | 4 | 4 | 4 | 4 | 2 |
| 1.7.1.15 | p__Gemmatimonadetes | 0 | 0 | 0 | 0 | 0 | 0 |
| 1.7.1.15 | p__Nitrospirae | 0 | 0 | 0 | 0 | 0 | 0 |
| 1.7.1.15 | p__unclassified_d__Bacteria | 0 | 0 | 0 | 0 | 0 | 0 |
| 1.7.1.15 | p__Cyanobacteria | 6 | 4 | 2 | 6 | 2 | 0 |
| 1.7.1.15 | p__Planctomycetes | 2 | 54 | 8 | 0 | 0 | 0 |
| 1.7.1.15 | p__Deinococcus-Thermus | 0 | 0 | 0 | 0 | 0 | 0 |
| 1.7.1.15 | p__Verrucomicrobia | 0 | 0 | 0 | 0 | 0 | 0 |
| 1.7.1.15 | p__Armatimonadetes | 0 | 0 | 0 | 0 | 0 | 0 |
| 1.7.1.15 | p__Candidatus_Tectomicrobia | 0 | 0 | 0 | 0 | 0 | 0 |
| 1.7.1.15 | p__Candidatus_Rokubacteria | 0 | 0 | 0 | 0 | 0 | 0 |
| 1.7.1.15 | p__candidate_division_NC10 | 0 | 0 | 0 | 0 | 0 | 0 |
| 1.7.1.15 | p__candidate_division_Zixibacteria | 0 | 0 | 0 | 0 | 0 | 0 |
| 1.7.1.15 | p__Spirochaetes | 0 | 0 | 0 | 0 | 0 | 0 |
| 1.7.1.15 | p__Candidatus_Acetothermia | 0 | 0 | 0 | 0 | 0 | 0 |
| 1.7.1.15 | p__Candidatus_Dadabacteria | 0 | 0 | 0 | 0 | 0 | 0 |
| 1.7.1.15 | p__Chlorobi | 0 | 0 | 0 | 0 | 0 | 0 |
| 1.7.1.15 | p__Candidatus_Omnitrophica | 0 | 0 | 0 | 0 | 0 | 0 |
| 1.7.1.15 | p__Aquificae | 0 | 0 | 0 | 0 | 0 | 0 |
| 1.7.1.15 | p__Thermodesulfobacteria | 0 | 0 | 0 | 0 | 0 | 0 |
| 1.7.1.15 | p__Latescibacteria | 0 | 0 | 0 | 0 | 0 | 0 |
| 1.7.1.15 | p__Candidatus_Gottesmanbacteria | 0 | 0 | 0 | 0 | 0 | 0 |
| 1.7.1.15 | p__Synergistetes | 0 | 0 | 0 | 0 | 0 | 0 |
| 1.7.1.15 | p__Candidatus_Woesebacteria | 0 | 0 | 0 | 0 | 0 | 0 |
| 1.7.1.15 | p__Thermotogae | 0 | 0 | 0 | 0 | 0 | 0 |
| 1.7.1.15 | others | 0 | 0 | 0 | 0 | 0 | 0 |
| 1.7.5.1 | p__Proteobacteria | 906 | 738 | 204 | 5952 | 6504 | 6374 |
| 1.7.5.1 | p__Actinobacteria | 332 | 280 | 358 | 402 | 452 | 432 |
| 1.7.5.1 | p__Bacteroidetes | 0 | 0 | 0 | 0 | 0 | 0 |
| 1.7.5.1 | p__Chloroflexi | 0 | 0 | 0 | 0 | 0 | 0 |
| 1.7.5.1 | p__Firmicutes | 6 | 0 | 0 | 20 | 28 | 28 |
| 1.7.5.1 | p__Acidobacteria | 0 | 0 | 0 | 0 | 0 | 0 |
| 1.7.5.1 | p__Gemmatimonadetes | 0 | 0 | 0 | 0 | 0 | 0 |
| 1.7.5.1 | p__Nitrospirae | 502 | 546 | 494 | 436 | 416 | 360 |
| 1.7.5.1 | p__unclassified_d__Bacteria | 0 | 0 | 0 | 0 | 0 | 0 |
| 1.7.5.1 | p__Cyanobacteria | 0 | 0 | 0 | 0 | 0 | 0 |
| 1.7.5.1 | p__Planctomycetes | 0 | 0 | 0 | 0 | 0 | 0 |
| 1.7.5.1 | p__Deinococcus-Thermus | 0 | 0 | 0 | 0 | 0 | 0 |
| 1.7.5.1 | p__Verrucomicrobia | 0 | 0 | 0 | 0 | 0 | 0 |
| 1.7.5.1 | p__Armatimonadetes | 0 | 0 | 0 | 0 | 0 | 0 |
| 1.7.5.1 | p__Candidatus_Tectomicrobia | 0 | 0 | 0 | 0 | 0 | 0 |
| 1.7.5.1 | p__Candidatus_Rokubacteria | 0 | 0 | 0 | 0 | 0 | 0 |
| 1.7.5.1 | p__candidate_division_NC10 | 0 | 0 | 0 | 0 | 0 | 0 |
| 1.7.5.1 | p__candidate_division_Zixibacteria | 0 | 0 | 0 | 0 | 0 | 0 |
| 1.7.5.1 | p__Spirochaetes | 0 | 0 | 0 | 0 | 0 | 0 |
| 1.7.5.1 | p__Candidatus_Acetothermia | 0 | 0 | 0 | 0 | 0 | 0 |
| 1.7.5.1 | p__Candidatus_Dadabacteria | 0 | 0 | 0 | 0 | 0 | 0 |
| 1.7.5.1 | p__Chlorobi | 0 | 0 | 0 | 0 | 0 | 0 |
| 1.7.5.1 | p__Candidatus_Omnitrophica | 0 | 0 | 0 | 0 | 0 | 0 |
| 1.7.5.1 | p__Aquificae | 0 | 0 | 0 | 0 | 0 | 0 |
| 1.7.5.1 | p__Thermodesulfobacteria | 0 | 0 | 0 | 0 | 0 | 0 |
| 1.7.5.1 | p__Latescibacteria | 0 | 0 | 0 | 0 | 0 | 0 |
| 1.7.5.1 | p__Candidatus_Gottesmanbacteria | 0 | 0 | 0 | 0 | 0 | 0 |
| 1.7.5.1 | p__Synergistetes | 0 | 0 | 0 | 0 | 0 | 0 |
| 1.7.5.1 | p__Candidatus_Woesebacteria | 0 | 0 | 0 | 0 | 0 | 0 |
| 1.7.5.1 | p__Thermotogae | 0 | 0 | 0 | 0 | 0 | 0 |
| 1.7.5.1 | others | 0 | 0 | 0 | 0 | 0 | 0 |
| 1.7.2.5 | p__Proteobacteria | 600 | 538 | 278 | 1964 | 2238 | 1904 |
| 1.7.2.5 | p__Actinobacteria | 14 | 16 | 40 | 32 | 46 | 34 |
| 1.7.2.5 | p__Bacteroidetes | 4 | 2 | 0 | 40 | 28 | 24 |
| 1.7.2.5 | p__Chloroflexi | 0 | 0 | 0 | 0 | 0 | 0 |
| 1.7.2.5 | p__Firmicutes | 0 | 0 | 0 | 48 | 54 | 8 |
| 1.7.2.5 | p__Acidobacteria | 0 | 0 | 0 | 0 | 0 | 0 |
| 1.7.2.5 | p__Gemmatimonadetes | 0 | 0 | 0 | 0 | 0 | 0 |
| 1.7.2.5 | p__Nitrospirae | 0 | 0 | 0 | 0 | 0 | 0 |
| 1.7.2.5 | p__unclassified_d__Bacteria | 0 | 0 | 0 | 0 | 0 | 0 |
| 1.7.2.5 | p__Cyanobacteria | 0 | 0 | 0 | 0 | 0 | 0 |
| 1.7.2.5 | p__Planctomycetes | 0 | 32 | 0 | 0 | 0 | 0 |
| 1.7.2.5 | p__Deinococcus-Thermus | 0 | 0 | 0 | 0 | 0 | 0 |
| 1.7.2.5 | p__Verrucomicrobia | 0 | 0 | 0 | 0 | 0 | 0 |
| 1.7.2.5 | p__Armatimonadetes | 0 | 0 | 0 | 0 | 0 | 0 |
| 1.7.2.5 | p__Candidatus_Tectomicrobia | 0 | 0 | 0 | 0 | 0 | 0 |
| 1.7.2.5 | p__Candidatus_Rokubacteria | 0 | 0 | 0 | 0 | 0 | 0 |
| 1.7.2.5 | p__candidate_division_NC10 | 0 | 0 | 0 | 0 | 0 | 0 |
| 1.7.2.5 | p__candidate_division_Zixibacteria | 0 | 0 | 0 | 0 | 0 | 0 |
| 1.7.2.5 | p__Spirochaetes | 0 | 0 | 0 | 0 | 0 | 0 |
| 1.7.2.5 | p__Candidatus_Acetothermia | 0 | 0 | 0 | 0 | 0 | 0 |
| 1.7.2.5 | p__Candidatus_Dadabacteria | 0 | 0 | 0 | 0 | 0 | 0 |
| 1.7.2.5 | p__Chlorobi | 0 | 0 | 0 | 0 | 0 | 0 |
| 1.7.2.5 | p__Candidatus_Omnitrophica | 0 | 0 | 0 | 0 | 0 | 0 |
| 1.7.2.5 | p__Aquificae | 0 | 0 | 0 | 0 | 0 | 0 |
| 1.7.2.5 | p__Thermodesulfobacteria | 0 | 0 | 0 | 0 | 0 | 0 |
| 1.7.2.5 | p__Latescibacteria | 0 | 0 | 0 | 0 | 0 | 0 |
| 1.7.2.5 | p__Candidatus_Gottesmanbacteria | 0 | 0 | 0 | 0 | 0 | 0 |
| 1.7.2.5 | p__Synergistetes | 0 | 0 | 0 | 0 | 0 | 0 |
| 1.7.2.5 | p__Candidatus_Woesebacteria | 0 | 0 | 0 | 0 | 0 | 0 |
| 1.7.2.5 | p__Thermotogae | 0 | 0 | 0 | 0 | 0 | 0 |
| 1.7.2.5 | others | 0 | 0 | 0 | 0 | 0 | 0 |
| 1.4.1.4 | p__Proteobacteria | 250 | 162 | 120 | 1348 | 1552 | 1376 |
| 1.4.1.4 | p__Actinobacteria | 146 | 144 | 122 | 148 | 150 | 148 |
| 1.4.1.4 | p__Bacteroidetes | 46 | 76 | 52 | 124 | 160 | 96 |
| 1.4.1.4 | p__Chloroflexi | 16 | 10 | 12 | 2 | 12 | 10 |
| 1.4.1.4 | p__Firmicutes | 0 | 0 | 0 | 6 | 8 | 8 |
| 1.4.1.4 | p__Acidobacteria | 0 | 0 | 0 | 0 | 0 | 0 |
| 1.4.1.4 | p__Gemmatimonadetes | 0 | 34 | 8 | 0 | 0 | 0 |
| 1.4.1.4 | p__Nitrospirae | 0 | 0 | 0 | 0 | 0 | 0 |
| 1.4.1.4 | p__unclassified_d__Bacteria | 38 | 14 | 16 | 6 | 22 | 10 |
| 1.4.1.4 | p__Cyanobacteria | 0 | 0 | 0 | 0 | 0 | 0 |
| 1.4.1.4 | p__Planctomycetes | 0 | 0 | 0 | 0 | 0 | 0 |
| 1.4.1.4 | p__Deinococcus-Thermus | 0 | 0 | 0 | 0 | 0 | 0 |
| 1.4.1.4 | p__Verrucomicrobia | 0 | 0 | 0 | 2 | 0 | 0 |
| 1.4.1.4 | p__Armatimonadetes | 0 | 0 | 0 | 0 | 0 | 0 |
| 1.4.1.4 | p__Candidatus_Tectomicrobia | 0 | 0 | 0 | 0 | 0 | 0 |
| 1.4.1.4 | p__Candidatus_Rokubacteria | 0 | 0 | 0 | 0 | 0 | 0 |
| 1.4.1.4 | p__candidate_division_NC10 | 0 | 0 | 0 | 0 | 0 | 0 |
| 1.4.1.4 | p__candidate_division_Zixibacteria | 0 | 0 | 0 | 0 | 0 | 0 |
| 1.4.1.4 | p__Spirochaetes | 0 | 0 | 0 | 0 | 0 | 0 |
| 1.4.1.4 | p__Candidatus_Acetothermia | 0 | 0 | 0 | 0 | 0 | 0 |
| 1.4.1.4 | p__Candidatus_Dadabacteria | 0 | 0 | 0 | 0 | 0 | 0 |
| 1.4.1.4 | p__Chlorobi | 0 | 0 | 0 | 0 | 0 | 0 |
| 1.4.1.4 | p__Candidatus_Omnitrophica | 0 | 0 | 0 | 0 | 0 | 0 |
| 1.4.1.4 | p__Aquificae | 0 | 0 | 0 | 0 | 0 | 0 |
| 1.4.1.4 | p__Thermodesulfobacteria | 0 | 0 | 0 | 0 | 0 | 0 |
| 1.4.1.4 | p__Latescibacteria | 0 | 0 | 0 | 0 | 0 | 0 |
| 1.4.1.4 | p__Candidatus_Gottesmanbacteria | 0 | 0 | 0 | 0 | 0 | 0 |
| 1.4.1.4 | p__Synergistetes | 0 | 0 | 0 | 0 | 0 | 0 |
| 1.4.1.4 | p__Candidatus_Woesebacteria | 0 | 0 | 0 | 0 | 0 | 0 |
| 1.4.1.4 | p__Thermotogae | 0 | 0 | 0 | 0 | 0 | 0 |
| 1.4.1.4 | others | 32 | 32 | 28 | 16 | 42 | 14 |
| 1.7.2.1 | p__Proteobacteria | 360 | 284 | 138 | 1350 | 1586 | 1320 |
| 1.7.2.1 | p__Actinobacteria | 74 | 58 | 94 | 216 | 212 | 148 |
| 1.7.2.1 | p__Bacteroidetes | 2 | 6 | 0 | 6 | 4 | 4 |
| 1.7.2.1 | p__Chloroflexi | 0 | 0 | 0 | 0 | 0 | 0 |
| 1.7.2.1 | p__Firmicutes | 0 | 0 | 0 | 0 | 0 | 0 |
| 1.7.2.1 | p__Acidobacteria | 0 | 0 | 0 | 0 | 0 | 0 |
| 1.7.2.1 | p__Gemmatimonadetes | 6 | 18 | 8 | 12 | 14 | 2 |
| 1.7.2.1 | p__Nitrospirae | 60 | 48 | 86 | 40 | 38 | 48 |
| 1.7.2.1 | p__unclassified_d__Bacteria | 54 | 68 | 88 | 36 | 34 | 38 |
| 1.7.2.1 | p__Cyanobacteria | 0 | 0 | 0 | 0 | 0 | 0 |
| 1.7.2.1 | p__Planctomycetes | 0 | 0 | 0 | 0 | 0 | 0 |
| 1.7.2.1 | p__Deinococcus-Thermus | 0 | 0 | 0 | 0 | 0 | 0 |
| 1.7.2.1 | p__Verrucomicrobia | 0 | 0 | 0 | 0 | 0 | 0 |
| 1.7.2.1 | p__Armatimonadetes | 0 | 0 | 0 | 0 | 0 | 0 |
| 1.7.2.1 | p__Candidatus_Tectomicrobia | 0 | 0 | 0 | 0 | 0 | 0 |
| 1.7.2.1 | p__Candidatus_Rokubacteria | 0 | 0 | 0 | 0 | 0 | 0 |
| 1.7.2.1 | p__candidate_division_NC10 | 0 | 0 | 0 | 0 | 0 | 0 |
| 1.7.2.1 | p__candidate_division_Zixibacteria | 0 | 0 | 0 | 0 | 0 | 0 |
| 1.7.2.1 | p__Spirochaetes | 2 | 0 | 0 | 0 | 0 | 0 |
| 1.7.2.1 | p__Candidatus_Acetothermia | 0 | 0 | 0 | 0 | 0 | 0 |
| 1.7.2.1 | p__Candidatus_Dadabacteria | 0 | 0 | 0 | 0 | 0 | 0 |
| 1.7.2.1 | p__Chlorobi | 0 | 0 | 0 | 0 | 0 | 0 |
| 1.7.2.1 | p__Candidatus_Omnitrophica | 0 | 0 | 0 | 0 | 0 | 0 |
| 1.7.2.1 | p__Aquificae | 0 | 0 | 0 | 0 | 0 | 0 |
| 1.7.2.1 | p__Thermodesulfobacteria | 0 | 0 | 0 | 0 | 0 | 0 |
| 1.7.2.1 | p__Latescibacteria | 0 | 0 | 0 | 0 | 0 | 0 |
| 1.7.2.1 | p__Candidatus_Gottesmanbacteria | 0 | 0 | 0 | 0 | 0 | 0 |
| 1.7.2.1 | p__Synergistetes | 0 | 0 | 0 | 0 | 0 | 0 |
| 1.7.2.1 | p__Candidatus_Woesebacteria | 0 | 0 | 0 | 0 | 0 | 0 |
| 1.7.2.1 | p__Thermotogae | 0 | 0 | 0 | 0 | 0 | 0 |
| 1.7.2.1 | others | 0 | 0 | 0 | 0 | 0 | 0 |
| 1.7.2.4 | p__Proteobacteria | 342 | 264 | 60 | 1352 | 1470 | 1312 |
| 1.7.2.4 | p__Actinobacteria | 6 | 18 | 12 | 6 | 12 | 12 |
| 1.7.2.4 | p__Bacteroidetes | 4 | 10 | 2 | 46 | 42 | 26 |
| 1.7.2.4 | p__Chloroflexi | 8 | 18 | 6 | 6 | 16 | 6 |
| 1.7.2.4 | p__Firmicutes | 0 | 0 | 0 | 16 | 16 | 6 |
| 1.7.2.4 | p__Acidobacteria | 2 | 0 | 0 | 0 | 0 | 0 |
| 1.7.2.4 | p__Gemmatimonadetes | 24 | 22 | 24 | 4 | 14 | 18 |
| 1.7.2.4 | p__Nitrospirae | 0 | 0 | 0 | 0 | 0 | 0 |
| 1.7.2.4 | p__unclassified_d__Bacteria | 0 | 2 | 2 | 12 | 2 | 2 |
| 1.7.2.4 | p__Cyanobacteria | 0 | 0 | 0 | 0 | 0 | 0 |
| 1.7.2.4 | p__Planctomycetes | 0 | 0 | 0 | 0 | 0 | 0 |
| 1.7.2.4 | p__Deinococcus-Thermus | 0 | 0 | 0 | 0 | 0 | 0 |
| 1.7.2.4 | p__Verrucomicrobia | 34 | 16 | 20 | 6 | 6 | 14 |
| 1.7.2.4 | p__Armatimonadetes | 0 | 0 | 0 | 0 | 0 | 0 |
| 1.7.2.4 | p__Candidatus_Tectomicrobia | 0 | 0 | 0 | 0 | 0 | 0 |
| 1.7.2.4 | p__Candidatus_Rokubacteria | 0 | 0 | 0 | 0 | 0 | 0 |
| 1.7.2.4 | p__candidate_division_NC10 | 0 | 0 | 0 | 0 | 0 | 0 |
| 1.7.2.4 | p__candidate_division_Zixibacteria | 0 | 0 | 0 | 0 | 0 | 0 |
| 1.7.2.4 | p__Spirochaetes | 0 | 0 | 0 | 0 | 0 | 0 |
| 1.7.2.4 | p__Candidatus_Acetothermia | 0 | 0 | 0 | 0 | 0 | 0 |
| 1.7.2.4 | p__Candidatus_Dadabacteria | 0 | 0 | 0 | 0 | 0 | 0 |
| 1.7.2.4 | p__Chlorobi | 0 | 0 | 0 | 0 | 0 | 0 |
| 1.7.2.4 | p__Candidatus_Omnitrophica | 0 | 0 | 0 | 0 | 0 | 0 |
| 1.7.2.4 | p__Aquificae | 0 | 0 | 0 | 0 | 0 | 0 |
| 1.7.2.4 | p__Thermodesulfobacteria | 0 | 0 | 0 | 0 | 0 | 0 |
| 1.7.2.4 | p__Latescibacteria | 0 | 0 | 0 | 0 | 0 | 0 |
| 1.7.2.4 | p__Candidatus_Gottesmanbacteria | 0 | 0 | 0 | 0 | 0 | 0 |
| 1.7.2.4 | p__Synergistetes | 0 | 0 | 0 | 0 | 0 | 0 |
| 1.7.2.4 | p__Candidatus_Woesebacteria | 0 | 0 | 0 | 0 | 0 | 0 |
| 1.7.2.4 | p__Thermotogae | 0 | 0 | 0 | 0 | 0 | 0 |
| 1.7.2.4 | others | 0 | 0 | 0 | 0 | 0 | 0 |
| 1.4.1.21 | p__Proteobacteria | 54 | 66 | 30 | 410 | 450 | 450 |
| 1.4.1.21 | p__Actinobacteria | 10 | 18 | 6 | 4 | 16 | 2 |
| 1.4.1.21 | p__Bacteroidetes | 0 | 0 | 0 | 0 | 0 | 0 |
| 1.4.1.21 | p__Chloroflexi | 0 | 0 | 0 | 0 | 0 | 0 |
| 1.4.1.21 | p__Firmicutes | 0 | 0 | 0 | 0 | 0 | 0 |
| 1.4.1.21 | p__Acidobacteria | 0 | 0 | 0 | 0 | 0 | 0 |
| 1.4.1.21 | p__Gemmatimonadetes | 0 | 0 | 0 | 0 | 0 | 0 |
| 1.4.1.21 | p__Nitrospirae | 0 | 0 | 0 | 0 | 0 | 0 |
| 1.4.1.21 | p__unclassified_d__Bacteria | 0 | 0 | 0 | 0 | 0 | 0 |
| 1.4.1.21 | p__Cyanobacteria | 0 | 0 | 0 | 0 | 0 | 0 |
| 1.4.1.21 | p__Planctomycetes | 0 | 0 | 0 | 0 | 0 | 0 |
| 1.4.1.21 | p__Deinococcus-Thermus | 0 | 0 | 0 | 0 | 0 | 0 |
| 1.4.1.21 | p__Verrucomicrobia | 0 | 0 | 0 | 0 | 0 | 0 |
| 1.4.1.21 | p__Armatimonadetes | 0 | 0 | 0 | 0 | 0 | 0 |
| 1.4.1.21 | p__Candidatus_Tectomicrobia | 0 | 0 | 0 | 0 | 0 | 0 |
| 1.4.1.21 | p__Candidatus_Rokubacteria | 0 | 0 | 0 | 0 | 0 | 0 |
| 1.4.1.21 | p__candidate_division_NC10 | 0 | 0 | 0 | 0 | 0 | 0 |
| 1.4.1.21 | p__candidate_division_Zixibacteria | 0 | 0 | 0 | 0 | 0 | 0 |
| 1.4.1.21 | p__Spirochaetes | 0 | 0 | 0 | 0 | 0 | 0 |
| 1.4.1.21 | p__Candidatus_Acetothermia | 0 | 0 | 0 | 0 | 0 | 0 |
| 1.4.1.21 | p__Candidatus_Dadabacteria | 0 | 0 | 0 | 0 | 0 | 0 |
| 1.4.1.21 | p__Chlorobi | 0 | 0 | 0 | 0 | 0 | 0 |
| 1.4.1.21 | p__Candidatus_Omnitrophica | 0 | 0 | 0 | 0 | 0 | 0 |
| 1.4.1.21 | p__Aquificae | 0 | 0 | 0 | 0 | 0 | 0 |
| 1.4.1.21 | p__Thermodesulfobacteria | 0 | 0 | 0 | 0 | 0 | 0 |
| 1.4.1.21 | p__Latescibacteria | 0 | 0 | 0 | 0 | 0 | 0 |
| 1.4.1.21 | p__Candidatus_Gottesmanbacteria | 0 | 0 | 0 | 0 | 0 | 0 |
| 1.4.1.21 | p__Synergistetes | 0 | 0 | 0 | 0 | 0 | 0 |
| 1.4.1.21 | p__Candidatus_Woesebacteria | 0 | 0 | 0 | 0 | 0 | 0 |
| 1.4.1.21 | p__Thermotogae | 0 | 0 | 0 | 0 | 0 | 0 |
| 1.4.1.21 | others | 0 | 0 | 0 | 0 | 0 | 0 |
| 1.18.6.1 | p__Proteobacteria | 36 | 34 | 6 | 288 | 388 | 352 |
| 1.18.6.1 | p__Actinobacteria | 0 | 0 | 0 | 0 | 0 | 0 |
| 1.18.6.1 | p__Bacteroidetes | 0 | 0 | 0 | 0 | 0 | 0 |
| 1.18.6.1 | p__Chloroflexi | 0 | 0 | 0 | 0 | 0 | 0 |
| 1.18.6.1 | p__Firmicutes | 2 | 0 | 0 | 40 | 62 | 44 |
| 1.18.6.1 | p__Acidobacteria | 0 | 0 | 0 | 0 | 0 | 0 |
| 1.18.6.1 | p__Gemmatimonadetes | 0 | 0 | 0 | 0 | 0 | 0 |
| 1.18.6.1 | p__Nitrospirae | 0 | 0 | 0 | 0 | 0 | 0 |
| 1.18.6.1 | p__unclassified_d__Bacteria | 0 | 0 | 0 | 0 | 0 | 0 |
| 1.18.6.1 | p__Cyanobacteria | 0 | 0 | 6 | 12 | 2 | 14 |
| 1.18.6.1 | p__Planctomycetes | 0 | 0 | 0 | 0 | 0 | 0 |
| 1.18.6.1 | p__Deinococcus-Thermus | 0 | 0 | 0 | 0 | 0 | 0 |
| 1.18.6.1 | p__Verrucomicrobia | 0 | 0 | 0 | 0 | 0 | 0 |
| 1.18.6.1 | p__Armatimonadetes | 0 | 0 | 0 | 0 | 0 | 0 |
| 1.18.6.1 | p__Candidatus_Tectomicrobia | 0 | 0 | 0 | 0 | 0 | 0 |
| 1.18.6.1 | p__Candidatus_Rokubacteria | 0 | 0 | 0 | 0 | 0 | 0 |
| 1.18.6.1 | p__candidate_division_NC10 | 0 | 0 | 0 | 0 | 0 | 0 |
| 1.18.6.1 | p__candidate_division_Zixibacteria | 0 | 0 | 0 | 0 | 0 | 0 |
| 1.18.6.1 | p__Spirochaetes | 0 | 0 | 0 | 0 | 0 | 0 |
| 1.18.6.1 | p__Candidatus_Acetothermia | 0 | 0 | 0 | 0 | 0 | 0 |
| 1.18.6.1 | p__Candidatus_Dadabacteria | 0 | 0 | 0 | 0 | 0 | 0 |
| 1.18.6.1 | p__Chlorobi | 0 | 0 | 0 | 0 | 0 | 0 |
| 1.18.6.1 | p__Candidatus_Omnitrophica | 0 | 0 | 0 | 0 | 0 | 0 |
| 1.18.6.1 | p__Aquificae | 0 | 0 | 0 | 0 | 0 | 0 |
| 1.18.6.1 | p__Thermodesulfobacteria | 0 | 0 | 0 | 0 | 0 | 0 |
| 1.18.6.1 | p__Latescibacteria | 0 | 0 | 0 | 0 | 0 | 0 |
| 1.18.6.1 | p__Candidatus_Gottesmanbacteria | 0 | 0 | 0 | 0 | 0 | 0 |
| 1.18.6.1 | p__Synergistetes | 0 | 0 | 0 | 0 | 0 | 0 |
| 1.18.6.1 | p__Candidatus_Woesebacteria | 0 | 0 | 0 | 0 | 0 | 0 |
| 1.18.6.1 | p__Thermotogae | 0 | 0 | 0 | 0 | 0 | 0 |
| 1.18.6.1 | others | 0 | 0 | 0 | 0 | 0 | 0 |

| Table S2. Distribution of bacteria communities at order level contributed to the significantly increased abundance of key enzymes in nitrogen metabolic pathways. | | | | | | | |
| --- | --- | --- | --- | --- | --- | --- | --- |
| Function | Taxon | T2Ck_a | T2Ck_b | T2Ck_c | T2B300_a | T2B300_b | T2B300_c |
| 1.7.99.4 | o__Burkholderiales | 704 | 630 | 248 | 2998 | 3480 | 2854 |
| 1.7.99.4 | o__Pseudomonadales | 822 | 562 | 72 | 2586 | 2744 | 2462 |
| 1.7.99.4 | o__Xanthomonadales | 210 | 168 | 176 | 828 | 906 | 950 |
| 1.7.99.4 | o__Solirubrobacterales | 12 | 10 | 26 | 2 | 20 | 4 |
| 1.7.99.4 | o__Rhizobiales | 396 | 322 | 264 | 756 | 946 | 796 |
| 1.7.99.4 | o__Enterobacteriales | 6 | 8 | 8 | 2912 | 3272 | 3674 |
| 1.7.99.4 | o__Propionibacteriales | 536 | 486 | 636 | 528 | 610 | 558 |
| 1.7.99.4 | o__Sphingomonadales | 222 | 244 | 194 | 400 | 398 | 400 |
| 1.7.99.4 | o__Micrococcales | 210 | 172 | 212 | 296 | 294 | 288 |
| 1.7.99.4 | o__Streptomycetales | 138 | 100 | 140 | 82 | 94 | 94 |
| 1.7.99.4 | o__Rhodospirillales | 28 | 2 | 8 | 420 | 514 | 454 |
| 1.7.99.4 | o__Rubrobacterales | 14 | 34 | 16 | 4 | 12 | 8 |
| 1.7.99.4 | o__Flavobacteriales | 32 | 38 | 16 | 98 | 76 | 38 |
| 1.7.99.4 | o__Neisseriales | 38 | 28 | 0 | 728 | 596 | 568 |
| 1.7.99.4 | o__Cellvibrionales | 32 | 8 | 40 | 144 | 146 | 122 |
| 1.7.99.4 | o__Pseudonocardiales | 22 | 16 | 44 | 10 | 20 | 20 |
| 1.7.99.4 | o__Corynebacteriales | 0 | 2 | 0 | 0 | 0 | 4 |
| 1.7.99.4 | o__Streptosporangiales | 50 | 46 | 36 | 8 | 24 | 26 |
| 1.7.99.4 | o__Gemmatimonadales | 0 | 0 | 0 | 0 | 0 | 0 |
| 1.7.99.4 | o__unclassified_p__Chloroflexi | 0 | 0 | 0 | 0 | 0 | 0 |
| 1.7.99.4 | o__Nitrospirales | 502 | 546 | 494 | 436 | 416 | 360 |
| 1.7.99.4 | o__Micromonosporales | 0 | 0 | 0 | 0 | 0 | 0 |
| 1.7.99.4 | o__Myxococcales | 0 | 48 | 2 | 2 | 2 | 2 |
| 1.7.99.4 | o__Gaiellales | 0 | 0 | 0 | 0 | 0 | 0 |
| 1.7.99.4 | o__unclassified_d__Bacteria | 6 | 12 | 10 | 0 | 2 | 0 |
| 1.7.99.4 | o__Clostridiales | 0 | 42 | 0 | 0 | 0 | 0 |
| 1.7.99.4 | o__unclassified_c__Actinobacteria | 0 | 4 | 6 | 0 | 0 | 0 |
| 1.7.99.4 | o__Aeromonadales | 2 | 0 | 0 | 28 | 92 | 56 |
| 1.7.99.4 | o__Geodermatophilales | 4 | 4 | 16 | 12 | 10 | 0 |
| 1.7.99.4 | o__unclassified_p__Acidobacteria | 8 | 16 | 12 | 2 | 8 | 2 |
| 1.7.99.4 | others | 234 | 250 | 254 | 334 | 398 | 380 |
| 1.7.1.15 | o__Burkholderiales | 430 | 408 | 202 | 1578 | 1680 | 1466 |
| 1.7.1.15 | o__Pseudomonadales | 338 | 234 | 24 | 1094 | 1192 | 1080 |
| 1.7.1.15 | o__Xanthomonadales | 64 | 58 | 64 | 248 | 222 | 236 |
| 1.7.1.15 | o__Solirubrobacterales | 144 | 136 | 98 | 104 | 184 | 96 |
| 1.7.1.15 | o__Rhizobiales | 248 | 222 | 174 | 368 | 488 | 376 |
| 1.7.1.15 | o__Enterobacteriales | 12 | 0 | 0 | 1610 | 1662 | 1816 |
| 1.7.1.15 | o__Propionibacteriales | 318 | 280 | 526 | 216 | 204 | 234 |
| 1.7.1.15 | o__Sphingomonadales | 152 | 180 | 128 | 320 | 362 | 248 |
| 1.7.1.15 | o__Micrococcales | 234 | 220 | 194 | 250 | 296 | 208 |
| 1.7.1.15 | o__Streptomycetales | 74 | 38 | 36 | 30 | 26 | 18 |
| 1.7.1.15 | o__Rhodospirillales | 4 | 4 | 0 | 138 | 154 | 118 |
| 1.7.1.15 | o__Rubrobacterales | 0 | 0 | 0 | 0 | 0 | 0 |
| 1.7.1.15 | o__Flavobacteriales | 26 | 42 | 16 | 92 | 34 | 20 |
| 1.7.1.15 | o__Neisseriales | 32 | 24 | 10 | 106 | 116 | 152 |
| 1.7.1.15 | o__Cellvibrionales | 84 | 50 | 76 | 222 | 176 | 160 |
| 1.7.1.15 | o__Pseudonocardiales | 34 | 70 | 50 | 26 | 42 | 16 |
| 1.7.1.15 | o__Corynebacteriales | 10 | 42 | 18 | 18 | 44 | 26 |
| 1.7.1.15 | o__Streptosporangiales | 0 | 0 | 0 | 0 | 0 | 0 |
| 1.7.1.15 | o__Gemmatimonadales | 0 | 0 | 0 | 0 | 0 | 0 |
| 1.7.1.15 | o__unclassified_p__Chloroflexi | 0 | 0 | 0 | 0 | 0 | 0 |
| 1.7.1.15 | o__Nitrospirales | 0 | 0 | 0 | 0 | 0 | 0 |
| 1.7.1.15 | o__Micromonosporales | 2 | 10 | 2 | 10 | 12 | 16 |
| 1.7.1.15 | o__Myxococcales | 0 | 0 | 0 | 0 | 0 | 0 |
| 1.7.1.15 | o__Gaiellales | 0 | 0 | 0 | 0 | 0 | 0 |
| 1.7.1.15 | o__unclassified_d__Bacteria | 0 | 0 | 0 | 0 | 0 | 0 |
| 1.7.1.15 | o__Clostridiales | 0 | 0 | 0 | 0 | 0 | 0 |
| 1.7.1.15 | o__unclassified_c__Actinobacteria | 0 | 0 | 0 | 0 | 0 | 0 |
| 1.7.1.15 | o__Aeromonadales | 4 | 0 | 0 | 54 | 128 | 98 |
| 1.7.1.15 | o__Geodermatophilales | 56 | 42 | 28 | 40 | 38 | 50 |
| 1.7.1.15 | o__unclassified_p__Acidobacteria | 4 | 4 | 4 | 4 | 4 | 2 |
| 1.7.1.15 | others | 180 | 208 | 160 | 318 | 306 | 250 |
| 1.7.5.1 | o__Burkholderiales | 360 | 340 | 74 | 1504 | 1818 | 1486 |
| 1.7.5.1 | o__Pseudomonadales | 398 | 268 | 20 | 1386 | 1402 | 1236 |
| 1.7.5.1 | o__Xanthomonadales | 12 | 10 | 8 | 162 | 114 | 138 |
| 1.7.5.1 | o__Solirubrobacterales | 0 | 0 | 0 | 0 | 0 | 0 |
| 1.7.5.1 | o__Rhizobiales | 100 | 86 | 76 | 114 | 100 | 112 |
| 1.7.5.1 | o__Enterobacteriales | 2 | 4 | 6 | 2216 | 2556 | 2878 |
| 1.7.5.1 | o__Propionibacteriales | 224 | 162 | 182 | 282 | 344 | 334 |
| 1.7.5.1 | o__Sphingomonadales | 2 | 8 | 4 | 2 | 6 | 0 |
| 1.7.5.1 | o__Micrococcales | 52 | 36 | 54 | 96 | 74 | 66 |
| 1.7.5.1 | o__Streptomycetales | 20 | 32 | 62 | 12 | 16 | 12 |
| 1.7.5.1 | o__Rhodospirillales | 0 | 0 | 0 | 0 | 0 | 0 |
| 1.7.5.1 | o__Rubrobacterales | 14 | 34 | 16 | 4 | 12 | 8 |
| 1.7.5.1 | o__Flavobacteriales | 0 | 0 | 0 | 0 | 0 | 0 |
| 1.7.5.1 | o__Neisseriales | 28 | 22 | 0 | 538 | 494 | 494 |
| 1.7.5.1 | o__Cellvibrionales | 0 | 0 | 0 | 0 | 0 | 0 |
| 1.7.5.1 | o__Pseudonocardiales | 22 | 14 | 42 | 8 | 6 | 8 |
| 1.7.5.1 | o__Corynebacteriales | 0 | 2 | 0 | 0 | 0 | 4 |
| 1.7.5.1 | o__Streptosporangiales | 0 | 0 | 0 | 0 | 0 | 0 |
| 1.7.5.1 | o__Gemmatimonadales | 0 | 0 | 0 | 0 | 0 | 0 |
| 1.7.5.1 | o__unclassified_p__Chloroflexi | 0 | 0 | 0 | 0 | 0 | 0 |
| 1.7.5.1 | o__Nitrospirales | 502 | 546 | 494 | 436 | 416 | 360 |
| 1.7.5.1 | o__Micromonosporales | 0 | 0 | 0 | 0 | 0 | 0 |
| 1.7.5.1 | o__Myxococcales | 0 | 0 | 0 | 0 | 0 | 0 |
| 1.7.5.1 | o__Gaiellales | 0 | 0 | 0 | 0 | 0 | 0 |
| 1.7.5.1 | o__unclassified_d__Bacteria | 0 | 0 | 0 | 0 | 0 | 0 |
| 1.7.5.1 | o__Clostridiales | 0 | 0 | 0 | 0 | 0 | 0 |
| 1.7.5.1 | o__unclassified_c__Actinobacteria | 0 | 0 | 0 | 0 | 0 | 0 |
| 1.7.5.1 | o__Aeromonadales | 0 | 0 | 0 | 0 | 0 | 0 |
| 1.7.5.1 | o__Geodermatophilales | 0 | 0 | 0 | 0 | 0 | 0 |
| 1.7.5.1 | o__unclassified_p__Acidobacteria | 0 | 0 | 0 | 0 | 0 | 0 |
| 1.7.5.1 | others | 10 | 0 | 18 | 50 | 42 | 58 |
| 1.7.2.5 | o__Burkholderiales | 206 | 166 | 64 | 534 | 640 | 530 |
| 1.7.2.5 | o__Pseudomonadales | 116 | 88 | 20 | 414 | 480 | 408 |
| 1.7.2.5 | o__Xanthomonadales | 176 | 126 | 118 | 460 | 408 | 420 |
| 1.7.2.5 | o__Solirubrobacterales | 0 | 0 | 0 | 0 | 0 | 0 |
| 1.7.2.5 | o__Rhizobiales | 14 | 16 | 4 | 152 | 176 | 110 |
| 1.7.2.5 | o__Enterobacteriales | 0 | 0 | 0 | 0 | 0 | 0 |
| 1.7.2.5 | o__Propionibacteriales | 0 | 0 | 0 | 0 | 0 | 0 |
| 1.7.2.5 | o__Sphingomonadales | 12 | 14 | 2 | 6 | 20 | 6 |
| 1.7.2.5 | o__Micrococcales | 0 | 0 | 0 | 0 | 0 | 0 |
| 1.7.2.5 | o__Streptomycetales | 0 | 0 | 0 | 0 | 0 | 0 |
| 1.7.2.5 | o__Rhodospirillales | 4 | 6 | 0 | 136 | 188 | 200 |
| 1.7.2.5 | o__Rubrobacterales | 0 | 0 | 0 | 0 | 0 | 0 |
| 1.7.2.5 | o__Flavobacteriales | 4 | 2 | 0 | 40 | 28 | 24 |
| 1.7.2.5 | o__Neisseriales | 0 | 0 | 0 | 0 | 0 | 0 |
| 1.7.2.5 | o__Cellvibrionales | 10 | 16 | 20 | 24 | 34 | 30 |
| 1.7.2.5 | o__Pseudonocardiales | 14 | 14 | 40 | 26 | 40 | 14 |
| 1.7.2.5 | o__Corynebacteriales | 0 | 2 | 0 | 6 | 6 | 20 |
| 1.7.2.5 | o__Streptosporangiales | 0 | 0 | 0 | 0 | 0 | 0 |
| 1.7.2.5 | o__Gemmatimonadales | 0 | 0 | 0 | 0 | 0 | 0 |
| 1.7.2.5 | o__unclassified_p__Chloroflexi | 0 | 0 | 0 | 0 | 0 | 0 |
| 1.7.2.5 | o__Nitrospirales | 0 | 0 | 0 | 0 | 0 | 0 |
| 1.7.2.5 | o__Micromonosporales | 0 | 0 | 0 | 0 | 0 | 0 |
| 1.7.2.5 | o__Myxococcales | 32 | 64 | 40 | 20 | 38 | 26 |
| 1.7.2.5 | o__Gaiellales | 0 | 0 | 0 | 0 | 0 | 0 |
| 1.7.2.5 | o__unclassified_d__Bacteria | 0 | 0 | 0 | 0 | 0 | 0 |
| 1.7.2.5 | o__Clostridiales | 0 | 0 | 0 | 0 | 0 | 0 |
| 1.7.2.5 | o__unclassified_c__Actinobacteria | 0 | 0 | 0 | 0 | 0 | 0 |
| 1.7.2.5 | o__Aeromonadales | 0 | 0 | 0 | 0 | 0 | 0 |
| 1.7.2.5 | o__Geodermatophilales | 0 | 0 | 0 | 0 | 0 | 0 |
| 1.7.2.5 | o__unclassified_p__Acidobacteria | 0 | 0 | 0 | 0 | 0 | 0 |
| 1.7.2.5 | others | 30 | 74 | 10 | 266 | 308 | 182 |
| 1.4.1.4 | o__Burkholderiales | 144 | 86 | 34 | 486 | 596 | 496 |
| 1.4.1.4 | o__Pseudomonadales | 4 | 4 | 2 | 156 | 152 | 166 |
| 1.4.1.4 | o__Xanthomonadales | 0 | 0 | 0 | 0 | 0 | 2 |
| 1.4.1.4 | o__Solirubrobacterales | 0 | 0 | 0 | 0 | 0 | 0 |
| 1.4.1.4 | o__Rhizobiales | 4 | 10 | 6 | 44 | 30 | 18 |
| 1.4.1.4 | o__Enterobacteriales | 0 | 0 | 0 | 364 | 360 | 386 |
| 1.4.1.4 | o__Propionibacteriales | 64 | 78 | 76 | 90 | 90 | 76 |
| 1.4.1.4 | o__Sphingomonadales | 38 | 24 | 30 | 76 | 66 | 42 |
| 1.4.1.4 | o__Micrococcales | 72 | 60 | 42 | 42 | 48 | 64 |
| 1.4.1.4 | o__Streptomycetales | 0 | 0 | 0 | 0 | 0 | 0 |
| 1.4.1.4 | o__Rhodospirillales | 2 | 6 | 0 | 70 | 78 | 74 |
| 1.4.1.4 | o__Rubrobacterales | 0 | 0 | 0 | 0 | 0 | 0 |
| 1.4.1.4 | o__Flavobacteriales | 42 | 64 | 38 | 108 | 126 | 82 |
| 1.4.1.4 | o__Neisseriales | 0 | 0 | 0 | 0 | 0 | 0 |
| 1.4.1.4 | o__Cellvibrionales | 14 | 2 | 10 | 62 | 62 | 58 |
| 1.4.1.4 | o__Pseudonocardiales | 0 | 0 | 0 | 0 | 0 | 0 |
| 1.4.1.4 | o__Corynebacteriales | 2 | 2 | 0 | 0 | 2 | 0 |
| 1.4.1.4 | o__Streptosporangiales | 0 | 0 | 4 | 0 | 0 | 0 |
| 1.4.1.4 | o__Gemmatimonadales | 0 | 34 | 8 | 0 | 0 | 0 |
| 1.4.1.4 | o__unclassified_p__Chloroflexi | 16 | 10 | 12 | 2 | 12 | 10 |
| 1.4.1.4 | o__Nitrospirales | 0 | 0 | 0 | 0 | 0 | 0 |
| 1.4.1.4 | o__Micromonosporales | 0 | 0 | 0 | 0 | 0 | 0 |
| 1.4.1.4 | o__Myxococcales | 0 | 0 | 0 | 0 | 0 | 0 |
| 1.4.1.4 | o__Gaiellales | 0 | 0 | 0 | 0 | 0 | 0 |
| 1.4.1.4 | o__unclassified_d__Bacteria | 38 | 14 | 16 | 6 | 22 | 10 |
| 1.4.1.4 | o__Clostridiales | 0 | 0 | 0 | 6 | 8 | 8 |
| 1.4.1.4 | o__unclassified_c__Actinobacteria | 0 | 0 | 0 | 0 | 0 | 0 |
| 1.4.1.4 | o__Aeromonadales | 0 | 0 | 0 | 28 | 102 | 58 |
| 1.4.1.4 | o__Geodermatophilales | 0 | 0 | 0 | 0 | 0 | 0 |
| 1.4.1.4 | o__unclassified_p__Acidobacteria | 0 | 0 | 0 | 0 | 0 | 0 |
| 1.4.1.4 | others | 88 | 78 | 80 | 112 | 192 | 112 |
| 1.7.2.1 | o__Burkholderiales | 48 | 46 | 22 | 216 | 302 | 256 |
| 1.7.2.1 | o__Pseudomonadales | 136 | 98 | 4 | 428 | 464 | 352 |
| 1.7.2.1 | o__Xanthomonadales | 108 | 78 | 84 | 340 | 408 | 288 |
| 1.7.2.1 | o__Solirubrobacterales | 0 | 0 | 0 | 0 | 0 | 0 |
| 1.7.2.1 | o__Rhizobiales | 32 | 14 | 12 | 156 | 190 | 176 |
| 1.7.2.1 | o__Enterobacteriales | 0 | 0 | 0 | 0 | 0 | 0 |
| 1.7.2.1 | o__Propionibacteriales | 16 | 6 | 14 | 106 | 58 | 58 |
| 1.7.2.1 | o__Sphingomonadales | 0 | 0 | 0 | 0 | 0 | 0 |
| 1.7.2.1 | o__Micrococcales | 0 | 0 | 0 | 10 | 6 | 24 |
| 1.7.2.1 | o__Streptomycetales | 0 | 0 | 0 | 0 | 0 | 0 |
| 1.7.2.1 | o__Rhodospirillales | 4 | 0 | 0 | 148 | 176 | 188 |
| 1.7.2.1 | o__Rubrobacterales | 0 | 0 | 0 | 0 | 0 | 0 |
| 1.7.2.1 | o__Flavobacteriales | 0 | 2 | 0 | 6 | 4 | 4 |
| 1.7.2.1 | o__Neisseriales | 0 | 0 | 0 | 0 | 0 | 0 |
| 1.7.2.1 | o__Cellvibrionales | 0 | 0 | 0 | 0 | 0 | 0 |
| 1.7.2.1 | o__Pseudonocardiales | 20 | 14 | 22 | 44 | 60 | 22 |
| 1.7.2.1 | o__Corynebacteriales | 2 | 12 | 20 | 22 | 12 | 4 |
| 1.7.2.1 | o__Streptosporangiales | 0 | 0 | 0 | 0 | 0 | 0 |
| 1.7.2.1 | o__Gemmatimonadales | 6 | 18 | 8 | 12 | 14 | 2 |
| 1.7.2.1 | o__unclassified_p__Chloroflexi | 0 | 0 | 0 | 0 | 0 | 0 |
| 1.7.2.1 | o__Nitrospirales | 60 | 48 | 86 | 40 | 38 | 48 |
| 1.7.2.1 | o__Micromonosporales | 6 | 2 | 6 | 2 | 0 | 4 |
| 1.7.2.1 | o__Myxococcales | 0 | 0 | 0 | 0 | 0 | 0 |
| 1.7.2.1 | o__Gaiellales | 0 | 0 | 0 | 0 | 0 | 0 |
| 1.7.2.1 | o__unclassified_d__Bacteria | 54 | 68 | 88 | 36 | 34 | 38 |
| 1.7.2.1 | o__Clostridiales | 0 | 0 | 0 | 0 | 0 | 0 |
| 1.7.2.1 | o__unclassified_c__Actinobacteria | 0 | 0 | 0 | 0 | 0 | 0 |
| 1.7.2.1 | o__Aeromonadales | 0 | 0 | 0 | 0 | 0 | 0 |
| 1.7.2.1 | o__Geodermatophilales | 24 | 8 | 16 | 24 | 20 | 22 |
| 1.7.2.1 | o__unclassified_p__Acidobacteria | 0 | 0 | 0 | 0 | 0 | 0 |
| 1.7.2.1 | others | 42 | 68 | 32 | 70 | 102 | 74 |
| 1.7.2.4 | o__Burkholderiales | 124 | 94 | 24 | 530 | 518 | 482 |
| 1.7.2.4 | o__Pseudomonadales | 122 | 84 | 2 | 508 | 494 | 470 |
| 1.7.2.4 | o__Xanthomonadales | 52 | 28 | 22 | 122 | 206 | 152 |
| 1.7.2.4 | o__Solirubrobacterales | 0 | 0 | 0 | 0 | 0 | 0 |
| 1.7.2.4 | o__Rhizobiales | 36 | 18 | 8 | 88 | 148 | 110 |
| 1.7.2.4 | o__Enterobacteriales | 0 | 0 | 0 | 0 | 0 | 0 |
| 1.7.2.4 | o__Propionibacteriales | 0 | 0 | 0 | 0 | 0 | 0 |
| 1.7.2.4 | o__Sphingomonadales | 0 | 0 | 0 | 0 | 0 | 0 |
| 1.7.2.4 | o__Micrococcales | 0 | 0 | 0 | 0 | 0 | 0 |
| 1.7.2.4 | o__Streptomycetales | 0 | 0 | 0 | 0 | 0 | 0 |
| 1.7.2.4 | o__Rhodospirillales | 0 | 4 | 4 | 78 | 94 | 92 |
| 1.7.2.4 | o__Rubrobacterales | 0 | 0 | 0 | 0 | 0 | 0 |
| 1.7.2.4 | o__Flavobacteriales | 2 | 6 | 0 | 40 | 28 | 22 |
| 1.7.2.4 | o__Neisseriales | 0 | 0 | 0 | 0 | 0 | 0 |
| 1.7.2.4 | o__Cellvibrionales | 0 | 0 | 0 | 0 | 0 | 0 |
| 1.7.2.4 | o__Pseudonocardiales | 0 | 0 | 0 | 0 | 0 | 0 |
| 1.7.2.4 | o__Corynebacteriales | 0 | 0 | 0 | 0 | 0 | 0 |
| 1.7.2.4 | o__Streptosporangiales | 0 | 0 | 0 | 0 | 0 | 0 |
| 1.7.2.4 | o__Gemmatimonadales | 24 | 22 | 24 | 4 | 14 | 18 |
| 1.7.2.4 | o__unclassified_p__Chloroflexi | 8 | 18 | 6 | 6 | 16 | 6 |
| 1.7.2.4 | o__Nitrospirales | 0 | 0 | 0 | 0 | 0 | 0 |
| 1.7.2.4 | o__Micromonosporales | 0 | 0 | 0 | 0 | 0 | 0 |
| 1.7.2.4 | o__Myxococcales | 4 | 32 | 0 | 0 | 0 | 0 |
| 1.7.2.4 | o__Gaiellales | 0 | 0 | 0 | 0 | 0 | 0 |
| 1.7.2.4 | o__unclassified_d__Bacteria | 0 | 2 | 2 | 12 | 2 | 2 |
| 1.7.2.4 | o__Clostridiales | 0 | 0 | 0 | 0 | 0 | 0 |
| 1.7.2.4 | o__unclassified_c__Actinobacteria | 0 | 0 | 0 | 0 | 0 | 0 |
| 1.7.2.4 | o__Aeromonadales | 0 | 0 | 0 | 0 | 0 | 0 |
| 1.7.2.4 | o__Geodermatophilales | 0 | 0 | 0 | 0 | 0 | 0 |
| 1.7.2.4 | o__unclassified_p__Acidobacteria | 2 | 0 | 0 | 0 | 0 | 0 |
| 1.7.2.4 | others | 46 | 42 | 34 | 60 | 58 | 42 |
| 1.4.1.21 | o__Burkholderiales | 20 | 28 | 4 | 226 | 264 | 234 |
| 1.4.1.21 | o__Pseudomonadales | 2 | 0 | 0 | 126 | 144 | 122 |
| 1.4.1.21 | o__Xanthomonadales | 0 | 0 | 0 | 0 | 0 | 0 |
| 1.4.1.21 | o__Solirubrobacterales | 0 | 0 | 0 | 0 | 0 | 0 |
| 1.4.1.21 | o__Rhizobiales | 0 | 0 | 0 | 0 | 0 | 0 |
| 1.4.1.21 | o__Enterobacteriales | 0 | 0 | 0 | 38 | 18 | 70 |
| 1.4.1.21 | o__Propionibacteriales | 6 | 2 | 2 | 0 | 6 | 0 |
| 1.4.1.21 | o__Sphingomonadales | 0 | 0 | 0 | 0 | 0 | 0 |
| 1.4.1.21 | o__Micrococcales | 4 | 16 | 4 | 4 | 10 | 2 |
| 1.4.1.21 | o__Streptomycetales | 0 | 0 | 0 | 0 | 0 | 0 |
| 1.4.1.21 | o__Rhodospirillales | 8 | 16 | 14 | 8 | 16 | 10 |
| 1.4.1.21 | o__Rubrobacterales | 0 | 0 | 0 | 0 | 0 | 0 |
| 1.4.1.21 | o__Flavobacteriales | 0 | 0 | 0 | 0 | 0 | 0 |
| 1.4.1.21 | o__Neisseriales | 0 | 0 | 0 | 0 | 0 | 0 |
| 1.4.1.21 | o__Cellvibrionales | 0 | 0 | 0 | 0 | 0 | 0 |
| 1.4.1.21 | o__Pseudonocardiales | 0 | 0 | 0 | 0 | 0 | 0 |
| 1.4.1.21 | o__Corynebacteriales | 0 | 0 | 0 | 0 | 0 | 0 |
| 1.4.1.21 | o__Streptosporangiales | 0 | 0 | 0 | 0 | 0 | 0 |
| 1.4.1.21 | o__Gemmatimonadales | 0 | 0 | 0 | 0 | 0 | 0 |
| 1.4.1.21 | o__unclassified_p__Chloroflexi | 0 | 0 | 0 | 0 | 0 | 0 |
| 1.4.1.21 | o__Nitrospirales | 0 | 0 | 0 | 0 | 0 | 0 |
| 1.4.1.21 | o__Micromonosporales | 0 | 0 | 0 | 0 | 0 | 0 |
| 1.4.1.21 | o__Myxococcales | 0 | 0 | 0 | 0 | 0 | 0 |
| 1.4.1.21 | o__Gaiellales | 0 | 0 | 0 | 0 | 0 | 0 |
| 1.4.1.21 | o__unclassified_d__Bacteria | 0 | 0 | 0 | 0 | 0 | 0 |
| 1.4.1.21 | o__Clostridiales | 0 | 0 | 0 | 0 | 0 | 0 |
| 1.4.1.21 | o__unclassified_c__Actinobacteria | 0 | 0 | 0 | 0 | 0 | 0 |
| 1.4.1.21 | o__Aeromonadales | 0 | 0 | 0 | 0 | 0 | 0 |
| 1.4.1.21 | o__Geodermatophilales | 0 | 0 | 0 | 0 | 0 | 0 |
| 1.4.1.21 | o__unclassified_p__Acidobacteria | 0 | 0 | 0 | 0 | 0 | 0 |
| 1.4.1.21 | others | 24 | 22 | 12 | 12 | 8 | 14 |
| 1.18.6.1 | o__Burkholderiales | 0 | 0 | 0 | 0 | 0 | 0 |
| 1.18.6.1 | o__Pseudomonadales | 22 | 20 | 0 | 60 | 76 | 52 |
| 1.18.6.1 | o__Xanthomonadales | 0 | 0 | 0 | 0 | 0 | 0 |
| 1.18.6.1 | o__Solirubrobacterales | 0 | 0 | 0 | 0 | 0 | 0 |
| 1.18.6.1 | o__Rhizobiales | 0 | 0 | 0 | 0 | 0 | 0 |
| 1.18.6.1 | o__Enterobacteriales | 0 | 0 | 0 | 0 | 0 | 0 |
| 1.18.6.1 | o__Propionibacteriales | 0 | 0 | 0 | 0 | 0 | 0 |
| 1.18.6.1 | o__Sphingomonadales | 0 | 0 | 0 | 0 | 0 | 0 |
| 1.18.6.1 | o__Micrococcales | 0 | 0 | 0 | 0 | 0 | 0 |
| 1.18.6.1 | o__Streptomycetales | 0 | 0 | 0 | 0 | 0 | 0 |
| 1.18.6.1 | o__Rhodospirillales | 12 | 12 | 4 | 214 | 308 | 284 |
| 1.18.6.1 | o__Rubrobacterales | 0 | 0 | 0 | 0 | 0 | 0 |
| 1.18.6.1 | o__Flavobacteriales | 0 | 0 | 0 | 0 | 0 | 0 |
| 1.18.6.1 | o__Neisseriales | 0 | 0 | 0 | 0 | 0 | 0 |
| 1.18.6.1 | o__Cellvibrionales | 0 | 0 | 0 | 0 | 0 | 0 |
| 1.18.6.1 | o__Pseudonocardiales | 0 | 0 | 0 | 0 | 0 | 0 |
| 1.18.6.1 | o__Corynebacteriales | 0 | 0 | 0 | 0 | 0 | 0 |
| 1.18.6.1 | o__Streptosporangiales | 0 | 0 | 0 | 0 | 0 | 0 |
| 1.18.6.1 | o__Gemmatimonadales | 0 | 0 | 0 | 0 | 0 | 0 |
| 1.18.6.1 | o__unclassified_p__Chloroflexi | 0 | 0 | 0 | 0 | 0 | 0 |
| 1.18.6.1 | o__Nitrospirales | 0 | 0 | 0 | 0 | 0 | 0 |
| 1.18.6.1 | o__Micromonosporales | 0 | 0 | 0 | 0 | 0 | 0 |
| 1.18.6.1 | o__Myxococcales | 0 | 0 | 0 | 0 | 0 | 0 |
| 1.18.6.1 | o__Gaiellales | 0 | 0 | 0 | 0 | 0 | 0 |
| 1.18.6.1 | o__unclassified_d__Bacteria | 0 | 0 | 0 | 0 | 0 | 0 |
| 1.18.6.1 | o__Clostridiales | 2 | 0 | 0 | 40 | 62 | 44 |
| 1.18.6.1 | o__unclassified_c__Actinobacteria | 0 | 0 | 0 | 0 | 0 | 0 |
| 1.18.6.1 | o__Aeromonadales | 0 | 0 | 0 | 0 | 0 | 0 |
| 1.18.6.1 | o__Geodermatophilales | 0 | 0 | 0 | 0 | 0 | 0 |
| 1.18.6.1 | o__unclassified_p__Acidobacteria | 0 | 0 | 0 | 0 | 0 | 0 |
| 1.18.6.1 | others | 2 | 2 | 8 | 26 | 6 | 30 |

| Table S3. Distribution of bacteria communities at genera level contributed to the significantly increased abundance of key enzymes in nitrogen metabolic pathways. | | | | | | | |
| --- | --- | --- | --- | --- | --- | --- | --- |
| Function | Taxon | T2Ck_a | T2Ck_b | T2Ck_c | T2B300_a | T2B300_b | T2B300_c |
| 1.7.99.4 | g__Pseudomonas | 822 | 562 | 72 | 2448 | 2654 | 2390 |
| 1.7.99.4 | g__Lelliottia | 0 | 4 | 8 | 2516 | 2834 | 3140 |
| 1.7.99.4 | g__Nocardioides | 476 | 412 | 548 | 404 | 506 | 408 |
| 1.7.99.4 | g__Pseudoxanthomonas | 156 | 134 | 124 | 612 | 650 | 722 |
| 1.7.99.4 | g__Achromobacter | 126 | 208 | 24 | 954 | 1126 | 998 |
| 1.7.99.4 | g__Solirubrobacter | 0 | 0 | 0 | 0 | 0 | 0 |
| 1.7.99.4 | g__Delftia | 0 | 2 | 0 | 766 | 934 | 756 |
| 1.7.99.4 | g__Acidovorax | 300 | 156 | 56 | 570 | 688 | 526 |
| 1.7.99.4 | g__Streptomyces | 134 | 84 | 138 | 72 | 92 | 84 |
| 1.7.99.4 | g__Conexibacter | 0 | 0 | 0 | 0 | 0 | 0 |
| 1.7.99.4 | g__Azospirillum | 10 | 0 | 6 | 414 | 510 | 444 |
| 1.7.99.4 | g__Rubrobacter | 14 | 34 | 16 | 4 | 12 | 8 |
| 1.7.99.4 | g__Lysobacter | 12 | 2 | 10 | 96 | 82 | 104 |
| 1.7.99.4 | g__Flavobacterium | 32 | 38 | 16 | 98 | 72 | 38 |
| 1.7.99.4 | g__Rhizobium | 36 | 32 | 44 | 60 | 106 | 56 |
| 1.7.99.4 | g__Agrobacterium | 58 | 34 | 10 | 238 | 288 | 260 |
| 1.7.99.4 | g__Vogesella | 38 | 28 | 0 | 728 | 596 | 568 |
| 1.7.99.4 | g__Sphingomonas | 106 | 114 | 124 | 154 | 174 | 164 |
| 1.7.99.4 | g__Cellvibrio | 30 | 8 | 28 | 144 | 146 | 122 |
| 1.7.99.4 | g__unclassified_o__Solirubrobacterales | 4 | 8 | 8 | 2 | 18 | 4 |
| 1.7.99.4 | g__Arthrobacter | 92 | 82 | 78 | 112 | 128 | 144 |
| 1.7.99.4 | g__Sphingobium | 34 | 14 | 18 | 68 | 44 | 42 |
| 1.7.99.4 | g__Sphingopyxis | 82 | 116 | 52 | 178 | 180 | 194 |
| 1.7.99.4 | g__Variovorax | 54 | 50 | 44 | 46 | 80 | 44 |
| 1.7.99.4 | g__Nitrospira | 502 | 546 | 494 | 436 | 416 | 360 |
| 1.7.99.4 | g__unclassified_p__Chloroflexi | 0 | 0 | 0 | 0 | 0 | 0 |
| 1.7.99.4 | g__Gaiella | 0 | 0 | 0 | 0 | 0 | 0 |
| 1.7.99.4 | g__Enterobacter | 0 | 2 | 0 | 50 | 136 | 414 |
| 1.7.99.4 | g__Phyllobacterium | 2 | 4 | 4 | 12 | 22 | 18 |
| 1.7.99.4 | g__Acinetobacter | 0 | 0 | 0 | 138 | 90 | 72 |
| 1.7.99.4 | others | 1108 | 1124 | 1008 | 2294 | 2496 | 2040 |
| 1.7.1.15 | g__Pseudomonas | 336 | 232 | 24 | 982 | 1106 | 1008 |
| 1.7.1.15 | g__Lelliottia | 0 | 0 | 0 | 0 | 0 | 0 |
| 1.7.1.15 | g__Nocardioides | 296 | 248 | 468 | 208 | 182 | 204 |
| 1.7.1.15 | g__Pseudoxanthomonas | 48 | 44 | 44 | 214 | 202 | 202 |
| 1.7.1.15 | g__Achromobacter | 86 | 80 | 24 | 400 | 474 | 390 |
| 1.7.1.15 | g__Solirubrobacter | 80 | 84 | 48 | 66 | 122 | 60 |
| 1.7.1.15 | g__Delftia | 4 | 0 | 0 | 358 | 332 | 294 |
| 1.7.1.15 | g__Acidovorax | 174 | 170 | 62 | 416 | 380 | 384 |
| 1.7.1.15 | g__Streptomyces | 74 | 38 | 36 | 30 | 26 | 18 |
| 1.7.1.15 | g__Conexibacter | 0 | 0 | 0 | 0 | 0 | 0 |
| 1.7.1.15 | g__Azospirillum | 4 | 4 | 0 | 138 | 154 | 118 |
| 1.7.1.15 | g__Rubrobacter | 0 | 0 | 0 | 0 | 0 | 0 |
| 1.7.1.15 | g__Lysobacter | 4 | 2 | 2 | 10 | 2 | 18 |
| 1.7.1.15 | g__Flavobacterium | 26 | 42 | 16 | 92 | 34 | 20 |
| 1.7.1.15 | g__Rhizobium | 48 | 24 | 26 | 82 | 78 | 90 |
| 1.7.1.15 | g__Agrobacterium | 0 | 0 | 0 | 24 | 10 | 14 |
| 1.7.1.15 | g__Vogesella | 14 | 6 | 0 | 78 | 84 | 132 |
| 1.7.1.15 | g__Sphingomonas | 14 | 8 | 12 | 18 | 30 | 28 |
| 1.7.1.15 | g__Cellvibrio | 46 | 46 | 44 | 206 | 156 | 154 |
| 1.7.1.15 | g__unclassified_o__Solirubrobacterales | 58 | 32 | 40 | 32 | 54 | 32 |
| 1.7.1.15 | g__Arthrobacter | 148 | 136 | 116 | 116 | 166 | 102 |
| 1.7.1.15 | g__Sphingobium | 70 | 74 | 84 | 142 | 162 | 118 |
| 1.7.1.15 | g__Sphingopyxis | 54 | 86 | 26 | 118 | 124 | 74 |
| 1.7.1.15 | g__Variovorax | 42 | 42 | 46 | 94 | 120 | 50 |
| 1.7.1.15 | g__Nitrospira | 0 | 0 | 0 | 0 | 0 | 0 |
| 1.7.1.15 | g__unclassified_p__Chloroflexi | 0 | 0 | 0 | 0 | 0 | 0 |
| 1.7.1.15 | g__Gaiella | 0 | 0 | 0 | 0 | 0 | 0 |
| 1.7.1.15 | g__Enterobacter | 10 | 0 | 0 | 850 | 1024 | 1146 |
| 1.7.1.15 | g__Phyllobacterium | 12 | 8 | 24 | 36 | 86 | 48 |
| 1.7.1.15 | g__Acinetobacter | 2 | 0 | 0 | 92 | 52 | 50 |
| 1.7.1.15 | others | 800 | 866 | 668 | 2044 | 2210 | 1932 |
| 1.7.5.1 | g__Pseudomonas | 398 | 268 | 20 | 1386 | 1402 | 1236 |
| 1.7.5.1 | g__Lelliottia | 0 | 2 | 6 | 1998 | 2252 | 2498 |
| 1.7.5.1 | g__Nocardioides | 194 | 148 | 156 | 194 | 254 | 218 |
| 1.7.5.1 | g__Pseudoxanthomonas | 0 | 0 | 0 | 0 | 0 | 0 |
| 1.7.5.1 | g__Achromobacter | 50 | 112 | 10 | 358 | 464 | 416 |
| 1.7.5.1 | g__Solirubrobacter | 0 | 0 | 0 | 0 | 0 | 0 |
| 1.7.5.1 | g__Delftia | 0 | 2 | 0 | 520 | 674 | 518 |
| 1.7.5.1 | g__Acidovorax | 218 | 124 | 32 | 422 | 502 | 392 |
| 1.7.5.1 | g__Streptomyces | 20 | 32 | 62 | 12 | 16 | 12 |
| 1.7.5.1 | g__Conexibacter | 0 | 0 | 0 | 0 | 0 | 0 |
| 1.7.5.1 | g__Azospirillum | 0 | 0 | 0 | 0 | 0 | 0 |
| 1.7.5.1 | g__Rubrobacter | 14 | 34 | 16 | 4 | 12 | 8 |
| 1.7.5.1 | g__Lysobacter | 4 | 0 | 0 | 84 | 66 | 86 |
| 1.7.5.1 | g__Flavobacterium | 0 | 0 | 0 | 0 | 0 | 0 |
| 1.7.5.1 | g__Rhizobium | 0 | 0 | 0 | 0 | 0 | 0 |
| 1.7.5.1 | g__Agrobacterium | 0 | 0 | 0 | 0 | 0 | 0 |
| 1.7.5.1 | g__Vogesella | 28 | 22 | 0 | 538 | 494 | 494 |
| 1.7.5.1 | g__Sphingomonas | 2 | 8 | 4 | 2 | 6 | 0 |
| 1.7.5.1 | g__Cellvibrio | 0 | 0 | 0 | 0 | 0 | 0 |
| 1.7.5.1 | g__unclassified_o__Solirubrobacterales | 0 | 0 | 0 | 0 | 0 | 0 |
| 1.7.5.1 | g__Arthrobacter | 0 | 0 | 0 | 0 | 0 | 0 |
| 1.7.5.1 | g__Sphingobium | 0 | 0 | 0 | 0 | 0 | 0 |
| 1.7.5.1 | g__Sphingopyxis | 0 | 0 | 0 | 0 | 0 | 0 |
| 1.7.5.1 | g__Variovorax | 20 | 26 | 8 | 22 | 38 | 16 |
| 1.7.5.1 | g__Nitrospira | 502 | 546 | 494 | 436 | 416 | 360 |
| 1.7.5.1 | g__unclassified_p__Chloroflexi | 0 | 0 | 0 | 0 | 0 | 0 |
| 1.7.5.1 | g__Gaiella | 0 | 0 | 0 | 0 | 0 | 0 |
| 1.7.5.1 | g__Enterobacter | 0 | 2 | 0 | 42 | 110 | 298 |
| 1.7.5.1 | g__Phyllobacterium | 0 | 0 | 0 | 0 | 0 | 0 |
| 1.7.5.1 | g__Acinetobacter | 0 | 0 | 0 | 0 | 0 | 0 |
| 1.7.5.1 | others | 296 | 238 | 248 | 792 | 694 | 642 |
| 1.7.2.5 | g__Pseudomonas | 116 | 88 | 20 | 414 | 480 | 408 |
| 1.7.2.5 | g__Lelliottia | 0 | 0 | 0 | 0 | 0 | 0 |
| 1.7.2.5 | g__Nocardioides | 0 | 0 | 0 | 0 | 0 | 0 |
| 1.7.2.5 | g__Pseudoxanthomonas | 106 | 66 | 42 | 354 | 328 | 320 |
| 1.7.2.5 | g__Achromobacter | 16 | 34 | 6 | 146 | 214 | 154 |
| 1.7.2.5 | g__Solirubrobacter | 0 | 0 | 0 | 0 | 0 | 0 |
| 1.7.2.5 | g__Delftia | 0 | 0 | 0 | 0 | 0 | 0 |
| 1.7.2.5 | g__Acidovorax | 112 | 84 | 22 | 220 | 250 | 222 |
| 1.7.2.5 | g__Streptomyces | 0 | 0 | 0 | 0 | 0 | 0 |
| 1.7.2.5 | g__Conexibacter | 0 | 0 | 0 | 0 | 0 | 0 |
| 1.7.2.5 | g__Azospirillum | 4 | 6 | 0 | 136 | 188 | 200 |
| 1.7.2.5 | g__Rubrobacter | 0 | 0 | 0 | 0 | 0 | 0 |
| 1.7.2.5 | g__Lysobacter | 22 | 38 | 32 | 64 | 56 | 74 |
| 1.7.2.5 | g__Flavobacterium | 2 | 2 | 0 | 40 | 28 | 24 |
| 1.7.2.5 | g__Rhizobium | 0 | 0 | 2 | 6 | 6 | 0 |
| 1.7.2.5 | g__Agrobacterium | 2 | 4 | 2 | 68 | 56 | 34 |
| 1.7.2.5 | g__Vogesella | 0 | 0 | 0 | 0 | 0 | 0 |
| 1.7.2.5 | g__Sphingomonas | 0 | 0 | 0 | 0 | 0 | 0 |
| 1.7.2.5 | g__Cellvibrio | 10 | 16 | 20 | 24 | 34 | 30 |
| 1.7.2.5 | g__unclassified_o__Solirubrobacterales | 0 | 0 | 0 | 0 | 0 | 0 |
| 1.7.2.5 | g__Arthrobacter | 0 | 0 | 0 | 0 | 0 | 0 |
| 1.7.2.5 | g__Sphingobium | 0 | 0 | 0 | 0 | 0 | 0 |
| 1.7.2.5 | g__Sphingopyxis | 0 | 0 | 0 | 0 | 0 | 0 |
| 1.7.2.5 | g__Variovorax | 24 | 12 | 16 | 12 | 28 | 18 |
| 1.7.2.5 | g__Nitrospira | 0 | 0 | 0 | 0 | 0 | 0 |
| 1.7.2.5 | g__unclassified_p__Chloroflexi | 0 | 0 | 0 | 0 | 0 | 0 |
| 1.7.2.5 | g__Gaiella | 0 | 0 | 0 | 0 | 0 | 0 |
| 1.7.2.5 | g__Enterobacter | 0 | 0 | 0 | 0 | 0 | 0 |
| 1.7.2.5 | g__Phyllobacterium | 0 | 0 | 0 | 0 | 0 | 0 |
| 1.7.2.5 | g__Acinetobacter | 0 | 0 | 0 | 0 | 0 | 0 |
| 1.7.2.5 | others | 204 | 238 | 156 | 600 | 698 | 486 |
| 1.4.1.4 | g__Pseudomonas | 4 | 4 | 2 | 110 | 136 | 156 |
| 1.4.1.4 | g__Lelliottia | 0 | 0 | 0 | 306 | 334 | 312 |
| 1.4.1.4 | g__Nocardioides | 48 | 72 | 68 | 74 | 52 | 56 |
| 1.4.1.4 | g__Pseudoxanthomonas | 0 | 0 | 0 | 0 | 0 | 0 |
| 1.4.1.4 | g__Achromobacter | 42 | 10 | 12 | 132 | 198 | 178 |
| 1.4.1.4 | g__Solirubrobacter | 0 | 0 | 0 | 0 | 0 | 0 |
| 1.4.1.4 | g__Delftia | 0 | 0 | 0 | 116 | 152 | 116 |
| 1.4.1.4 | g__Acidovorax | 68 | 60 | 6 | 136 | 180 | 138 |
| 1.4.1.4 | g__Streptomyces | 0 | 0 | 0 | 0 | 0 | 0 |
| 1.4.1.4 | g__Conexibacter | 0 | 0 | 0 | 0 | 0 | 0 |
| 1.4.1.4 | g__Azospirillum | 2 | 6 | 0 | 70 | 78 | 74 |
| 1.4.1.4 | g__Rubrobacter | 0 | 0 | 0 | 0 | 0 | 0 |
| 1.4.1.4 | g__Lysobacter | 0 | 0 | 0 | 0 | 0 | 0 |
| 1.4.1.4 | g__Flavobacterium | 42 | 64 | 38 | 100 | 118 | 78 |
| 1.4.1.4 | g__Rhizobium | 0 | 0 | 0 | 0 | 0 | 0 |
| 1.4.1.4 | g__Agrobacterium | 0 | 0 | 0 | 0 | 0 | 0 |
| 1.4.1.4 | g__Vogesella | 0 | 0 | 0 | 0 | 0 | 0 |
| 1.4.1.4 | g__Sphingomonas | 6 | 10 | 10 | 42 | 36 | 26 |
| 1.4.1.4 | g__Cellvibrio | 14 | 2 | 10 | 62 | 62 | 58 |
| 1.4.1.4 | g__unclassified_o__Solirubrobacterales | 0 | 0 | 0 | 0 | 0 | 0 |
| 1.4.1.4 | g__Arthrobacter | 58 | 46 | 36 | 32 | 46 | 40 |
| 1.4.1.4 | g__Sphingobium | 0 | 0 | 0 | 0 | 0 | 0 |
| 1.4.1.4 | g__Sphingopyxis | 32 | 10 | 14 | 30 | 24 | 16 |
| 1.4.1.4 | g__Variovorax | 0 | 0 | 2 | 10 | 0 | 4 |
| 1.4.1.4 | g__Nitrospira | 0 | 0 | 0 | 0 | 0 | 0 |
| 1.4.1.4 | g__unclassified_p__Chloroflexi | 16 | 10 | 12 | 2 | 12 | 10 |
| 1.4.1.4 | g__Gaiella | 0 | 0 | 0 | 0 | 0 | 0 |
| 1.4.1.4 | g__Enterobacter | 0 | 0 | 0 | 0 | 2 | 54 |
| 1.4.1.4 | g__Phyllobacterium | 0 | 0 | 0 | 0 | 0 | 0 |
| 1.4.1.4 | g__Acinetobacter | 0 | 0 | 0 | 46 | 16 | 10 |
| 1.4.1.4 | others | 196 | 178 | 148 | 384 | 500 | 336 |
| 1.7.2.1 | g__Pseudomonas | 136 | 98 | 4 | 428 | 464 | 352 |
| 1.7.2.1 | g__Lelliottia | 0 | 0 | 0 | 0 | 0 | 0 |
| 1.7.2.1 | g__Nocardioides | 16 | 6 | 14 | 106 | 58 | 58 |
| 1.7.2.1 | g__Pseudoxanthomonas | 36 | 26 | 34 | 168 | 142 | 112 |
| 1.7.2.1 | g__Achromobacter | 22 | 30 | 4 | 70 | 140 | 104 |
| 1.7.2.1 | g__Solirubrobacter | 0 | 0 | 0 | 0 | 0 | 0 |
| 1.7.2.1 | g__Delftia | 0 | 0 | 0 | 0 | 0 | 0 |
| 1.7.2.1 | g__Acidovorax | 0 | 0 | 0 | 0 | 0 | 0 |
| 1.7.2.1 | g__Streptomyces | 0 | 0 | 0 | 0 | 0 | 0 |
| 1.7.2.1 | g__Conexibacter | 0 | 0 | 0 | 0 | 0 | 0 |
| 1.7.2.1 | g__Azospirillum | 4 | 0 | 0 | 132 | 168 | 152 |
| 1.7.2.1 | g__Rubrobacter | 0 | 0 | 0 | 0 | 0 | 0 |
| 1.7.2.1 | g__Lysobacter | 28 | 18 | 36 | 62 | 88 | 52 |
| 1.7.2.1 | g__Flavobacterium | 0 | 2 | 0 | 6 | 4 | 4 |
| 1.7.2.1 | g__Rhizobium | 4 | 6 | 2 | 14 | 32 | 4 |
| 1.7.2.1 | g__Agrobacterium | 4 | 4 | 6 | 86 | 70 | 86 |
| 1.7.2.1 | g__Vogesella | 0 | 0 | 0 | 0 | 0 | 0 |
| 1.7.2.1 | g__Sphingomonas | 0 | 0 | 0 | 0 | 0 | 0 |
| 1.7.2.1 | g__Cellvibrio | 0 | 0 | 0 | 0 | 0 | 0 |
| 1.7.2.1 | g__unclassified_o__Solirubrobacterales | 0 | 0 | 0 | 0 | 0 | 0 |
| 1.7.2.1 | g__Arthrobacter | 0 | 0 | 0 | 0 | 0 | 0 |
| 1.7.2.1 | g__Sphingobium | 0 | 0 | 0 | 0 | 0 | 0 |
| 1.7.2.1 | g__Sphingopyxis | 0 | 0 | 0 | 0 | 0 | 0 |
| 1.7.2.1 | g__Variovorax | 0 | 0 | 0 | 0 | 0 | 0 |
| 1.7.2.1 | g__Nitrospira | 60 | 48 | 86 | 40 | 38 | 48 |
| 1.7.2.1 | g__unclassified_p__Chloroflexi | 0 | 0 | 0 | 0 | 0 | 0 |
| 1.7.2.1 | g__Gaiella | 0 | 0 | 0 | 0 | 0 | 0 |
| 1.7.2.1 | g__Enterobacter | 0 | 0 | 0 | 0 | 0 | 0 |
| 1.7.2.1 | g__Phyllobacterium | 0 | 0 | 0 | 0 | 0 | 0 |
| 1.7.2.1 | g__Acinetobacter | 0 | 0 | 0 | 0 | 0 | 0 |
| 1.7.2.1 | others | 248 | 244 | 228 | 548 | 684 | 588 |
| 1.7.2.4 | g__Pseudomonas | 122 | 84 | 2 | 508 | 494 | 470 |
| 1.7.2.4 | g__Lelliottia | 0 | 0 | 0 | 0 | 0 | 0 |
| 1.7.2.4 | g__Nocardioides | 0 | 0 | 0 | 0 | 0 | 0 |
| 1.7.2.4 | g__Pseudoxanthomonas | 0 | 0 | 0 | 0 | 0 | 0 |
| 1.7.2.4 | g__Achromobacter | 26 | 54 | 4 | 160 | 188 | 188 |
| 1.7.2.4 | g__Solirubrobacter | 0 | 0 | 0 | 0 | 0 | 0 |
| 1.7.2.4 | g__Delftia | 0 | 0 | 0 | 0 | 0 | 0 |
| 1.7.2.4 | g__Acidovorax | 80 | 40 | 16 | 214 | 206 | 170 |
| 1.7.2.4 | g__Streptomyces | 0 | 0 | 0 | 0 | 0 | 0 |
| 1.7.2.4 | g__Conexibacter | 0 | 0 | 0 | 0 | 0 | 0 |
| 1.7.2.4 | g__Azospirillum | 0 | 4 | 4 | 78 | 94 | 92 |
| 1.7.2.4 | g__Rubrobacter | 0 | 0 | 0 | 0 | 0 | 0 |
| 1.7.2.4 | g__Lysobacter | 0 | 0 | 0 | 0 | 0 | 0 |
| 1.7.2.4 | g__Flavobacterium | 2 | 6 | 0 | 40 | 28 | 22 |
| 1.7.2.4 | g__Rhizobium | 18 | 10 | 0 | 66 | 86 | 68 |
| 1.7.2.4 | g__Agrobacterium | 0 | 0 | 0 | 0 | 0 | 0 |
| 1.7.2.4 | g__Vogesella | 0 | 0 | 0 | 0 | 0 | 0 |
| 1.7.2.4 | g__Sphingomonas | 0 | 0 | 0 | 0 | 0 | 0 |
| 1.7.2.4 | g__Cellvibrio | 0 | 0 | 0 | 0 | 0 | 0 |
| 1.7.2.4 | g__unclassified_o__Solirubrobacterales | 0 | 0 | 0 | 0 | 0 | 0 |
| 1.7.2.4 | g__Arthrobacter | 0 | 0 | 0 | 0 | 0 | 0 |
| 1.7.2.4 | g__Sphingobium | 0 | 0 | 0 | 0 | 0 | 0 |
| 1.7.2.4 | g__Sphingopyxis | 0 | 0 | 0 | 0 | 0 | 0 |
| 1.7.2.4 | g__Variovorax | 0 | 0 | 0 | 0 | 0 | 0 |
| 1.7.2.4 | g__Nitrospira | 0 | 0 | 0 | 0 | 0 | 0 |
| 1.7.2.4 | g__unclassified_p__Chloroflexi | 8 | 18 | 6 | 6 | 16 | 6 |
| 1.7.2.4 | g__Gaiella | 0 | 0 | 0 | 0 | 0 | 0 |
| 1.7.2.4 | g__Enterobacter | 0 | 0 | 0 | 0 | 0 | 0 |
| 1.7.2.4 | g__Phyllobacterium | 0 | 0 | 0 | 0 | 0 | 0 |
| 1.7.2.4 | g__Acinetobacter | 0 | 0 | 0 | 0 | 0 | 0 |
| 1.7.2.4 | others | 164 | 134 | 94 | 376 | 466 | 380 |
| 1.4.1.21 | g__Pseudomonas | 2 | 0 | 0 | 72 | 92 | 58 |
| 1.4.1.21 | g__Lelliottia | 0 | 0 | 0 | 0 | 0 | 0 |
| 1.4.1.21 | g__Nocardioides | 6 | 2 | 2 | 0 | 6 | 0 |
| 1.4.1.21 | g__Pseudoxanthomonas | 0 | 0 | 0 | 0 | 0 | 0 |
| 1.4.1.21 | g__Achromobacter | 8 | 20 | 2 | 110 | 148 | 94 |
| 1.4.1.21 | g__Solirubrobacter | 0 | 0 | 0 | 0 | 0 | 0 |
| 1.4.1.21 | g__Delftia | 0 | 0 | 2 | 98 | 98 | 126 |
| 1.4.1.21 | g__Acidovorax | 0 | 0 | 0 | 0 | 0 | 0 |
| 1.4.1.21 | g__Streptomyces | 0 | 0 | 0 | 0 | 0 | 0 |
| 1.4.1.21 | g__Conexibacter | 0 | 0 | 0 | 0 | 0 | 0 |
| 1.4.1.21 | g__Azospirillum | 0 | 0 | 0 | 0 | 0 | 0 |
| 1.4.1.21 | g__Rubrobacter | 0 | 0 | 0 | 0 | 0 | 0 |
| 1.4.1.21 | g__Lysobacter | 0 | 0 | 0 | 0 | 0 | 0 |
| 1.4.1.21 | g__Flavobacterium | 0 | 0 | 0 | 0 | 0 | 0 |
| 1.4.1.21 | g__Rhizobium | 0 | 0 | 0 | 0 | 0 | 0 |
| 1.4.1.21 | g__Agrobacterium | 0 | 0 | 0 | 0 | 0 | 0 |
| 1.4.1.21 | g__Vogesella | 0 | 0 | 0 | 0 | 0 | 0 |
| 1.4.1.21 | g__Sphingomonas | 0 | 0 | 0 | 0 | 0 | 0 |
| 1.4.1.21 | g__Cellvibrio | 0 | 0 | 0 | 0 | 0 | 0 |
| 1.4.1.21 | g__unclassified_o__Solirubrobacterales | 0 | 0 | 0 | 0 | 0 | 0 |
| 1.4.1.21 | g__Arthrobacter | 0 | 0 | 0 | 0 | 0 | 0 |
| 1.4.1.21 | g__Sphingobium | 0 | 0 | 0 | 0 | 0 | 0 |
| 1.4.1.21 | g__Sphingopyxis | 0 | 0 | 0 | 0 | 0 | 0 |
| 1.4.1.21 | g__Variovorax | 4 | 0 | 0 | 6 | 0 | 2 |
| 1.4.1.21 | g__Nitrospira | 0 | 0 | 0 | 0 | 0 | 0 |
| 1.4.1.21 | g__unclassified_p__Chloroflexi | 0 | 0 | 0 | 0 | 0 | 0 |
| 1.4.1.21 | g__Gaiella | 0 | 0 | 0 | 0 | 0 | 0 |
| 1.4.1.21 | g__Enterobacter | 0 | 0 | 0 | 16 | 10 | 60 |
| 1.4.1.21 | g__Phyllobacterium | 0 | 0 | 0 | 0 | 0 | 0 |
| 1.4.1.21 | g__Acinetobacter | 0 | 0 | 0 | 54 | 52 | 64 |
| 1.4.1.21 | others | 44 | 62 | 30 | 58 | 60 | 48 |
| 1.18.6.1 | g__Pseudomonas | 0 | 0 | 0 | 0 | 0 | 0 |
| 1.18.6.1 | g__Lelliottia | 0 | 0 | 0 | 0 | 0 | 0 |
| 1.18.6.1 | g__Nocardioides | 0 | 0 | 0 | 0 | 0 | 0 |
| 1.18.6.1 | g__Pseudoxanthomonas | 0 | 0 | 0 | 0 | 0 | 0 |
| 1.18.6.1 | g__Achromobacter | 0 | 0 | 0 | 0 | 0 | 0 |
| 1.18.6.1 | g__Solirubrobacter | 0 | 0 | 0 | 0 | 0 | 0 |
| 1.18.6.1 | g__Delftia | 0 | 0 | 0 | 0 | 0 | 0 |
| 1.18.6.1 | g__Acidovorax | 0 | 0 | 0 | 0 | 0 | 0 |
| 1.18.6.1 | g__Streptomyces | 0 | 0 | 0 | 0 | 0 | 0 |
| 1.18.6.1 | g__Conexibacter | 0 | 0 | 0 | 0 | 0 | 0 |
| 1.18.6.1 | g__Azospirillum | 12 | 12 | 4 | 214 | 308 | 284 |
| 1.18.6.1 | g__Rubrobacter | 0 | 0 | 0 | 0 | 0 | 0 |
| 1.18.6.1 | g__Lysobacter | 0 | 0 | 0 | 0 | 0 | 0 |
| 1.18.6.1 | g__Flavobacterium | 0 | 0 | 0 | 0 | 0 | 0 |
| 1.18.6.1 | g__Rhizobium | 0 | 0 | 0 | 0 | 0 | 0 |
| 1.18.6.1 | g__Agrobacterium | 0 | 0 | 0 | 0 | 0 | 0 |
| 1.18.6.1 | g__Vogesella | 0 | 0 | 0 | 0 | 0 | 0 |
| 1.18.6.1 | g__Sphingomonas | 0 | 0 | 0 | 0 | 0 | 0 |
| 1.18.6.1 | g__Cellvibrio | 0 | 0 | 0 | 0 | 0 | 0 |
| 1.18.6.1 | g__unclassified_o__Solirubrobacterales | 0 | 0 | 0 | 0 | 0 | 0 |
| 1.18.6.1 | g__Arthrobacter | 0 | 0 | 0 | 0 | 0 | 0 |
| 1.18.6.1 | g__Sphingobium | 0 | 0 | 0 | 0 | 0 | 0 |
| 1.18.6.1 | g__Sphingopyxis | 0 | 0 | 0 | 0 | 0 | 0 |
| 1.18.6.1 | g__Variovorax | 0 | 0 | 0 | 0 | 0 | 0 |
| 1.18.6.1 | g__Nitrospira | 0 | 0 | 0 | 0 | 0 | 0 |
| 1.18.6.1 | g__unclassified_p__Chloroflexi | 0 | 0 | 0 | 0 | 0 | 0 |
| 1.18.6.1 | g__Gaiella | 0 | 0 | 0 | 0 | 0 | 0 |
| 1.18.6.1 | g__Enterobacter | 0 | 0 | 0 | 0 | 0 | 0 |
| 1.18.6.1 | g__Phyllobacterium | 0 | 0 | 0 | 0 | 0 | 0 |
| 1.18.6.1 | g__Acinetobacter | 0 | 0 | 0 | 0 | 0 | 0 |
| 1.18.6.1 | others | 26 | 22 | 8 | 126 | 144 | 126 |

| Table S4. Distribution of bacteria communities at phyla level contributed to the significantly increased abundance of key enzymes in carbon fixation pathways. | | | | | | | |
| --- | --- | --- | --- | --- | --- | --- | --- |
| Function | Taxon | T2Ck_a | T2Ck_b | T2Ck_c | T2B300_a | T2B300_b | T2B300_c |
| 6.4.1.2 | p__Proteobacteria | 2378 | 2346 | 1454 | 8454 | 9448 | 8148 |
| 6.4.1.2 | p__Actinobacteria | 3142 | 3200 | 3880 | 2720 | 3338 | 2522 |
| 6.4.1.2 | p__Bacteroidetes | 158 | 218 | 94 | 368 | 402 | 188 |
| 6.4.1.2 | p__Chloroflexi | 378 | 374 | 428 | 258 | 274 | 280 |
| 6.4.1.2 | p__Firmicutes | 22 | 92 | 18 | 68 | 100 | 62 |
| 6.4.1.2 | p__Acidobacteria | 228 | 242 | 246 | 148 | 138 | 146 |
| 6.4.1.2 | p__Gemmatimonadetes | 204 | 272 | 240 | 142 | 180 | 192 |
| 6.4.1.2 | p__Nitrospirae | 88 | 162 | 154 | 76 | 128 | 92 |
| 6.4.1.2 | p__unclassified_d__Bacteria | 32 | 30 | 30 | 22 | 52 | 42 |
| 6.4.1.2 | p__Cyanobacteria | 20 | 30 | 22 | 36 | 38 | 26 |
| 6.4.1.2 | p__Planctomycetes | 6 | 22 | 6 | 2 | 8 | 2 |
| 6.4.1.2 | p__Deinococcus-Thermus | 166 | 222 | 224 | 176 | 188 | 160 |
| 6.4.1.2 | p__Verrucomicrobia | 12 | 14 | 22 | 24 | 12 | 12 |
| 6.4.1.2 | p__Armatimonadetes | 10 | 16 | 20 | 2 | 6 | 4 |
| 6.4.1.2 | p__Candidatus_Tectomicrobia | 0 | 0 | 0 | 0 | 0 | 0 |
| 6.4.1.2 | p__Candidatus_Rokubacteria | 12 | 24 | 24 | 24 | 16 | 24 |
| 6.4.1.2 | p__candidate_division_NC10 | 0 | 0 | 0 | 0 | 0 | 0 |
| 6.4.1.2 | p__candidate_division_Zixibacteria | 0 | 0 | 0 | 0 | 0 | 0 |
| 6.4.1.2 | p__Spirochaetes | 0 | 0 | 0 | 0 | 0 | 0 |
| 6.4.1.2 | p__Candidatus_Acetothermia | 0 | 0 | 0 | 4 | 2 | 0 |
| 6.4.1.2 | p__Candidatus_Dadabacteria | 0 | 0 | 0 | 0 | 0 | 0 |
| 6.4.1.2 | p__Chlorobi | 0 | 0 | 0 | 0 | 0 | 0 |
| 6.4.1.2 | p__Candidatus_Omnitrophica | 0 | 0 | 0 | 0 | 0 | 0 |
| 6.4.1.2 | p__Aquificae | 22 | 16 | 28 | 22 | 30 | 26 |
| 6.4.1.2 | p__Thermodesulfobacteria | 0 | 0 | 0 | 0 | 0 | 0 |
| 6.4.1.2 | p__Latescibacteria | 0 | 0 | 0 | 0 | 0 | 0 |
| 6.4.1.2 | p__Candidatus_Gottesmanbacteria | 10 | 10 | 22 | 4 | 6 | 14 |
| 6.4.1.2 | p__Synergistetes | 0 | 0 | 0 | 0 | 0 | 0 |
| 6.4.1.2 | p__Candidatus_Woesebacteria | 0 | 0 | 0 | 0 | 0 | 0 |
| 6.4.1.2 | p__Thermotogae | 0 | 0 | 0 | 0 | 0 | 0 |
| 6.4.1.2 | others | 0 | 0 | 0 | 0 | 0 | 0 |
| 4.2.1.2 | p__Proteobacteria | 1572 | 1700 | 962 | 5620 | 6494 | 5894 |
| 4.2.1.2 | p__Actinobacteria | 1594 | 1694 | 1786 | 1282 | 1532 | 1258 |
| 4.2.1.2 | p__Bacteroidetes | 118 | 210 | 68 | 262 | 236 | 158 |
| 4.2.1.2 | p__Chloroflexi | 20 | 26 | 26 | 16 | 12 | 22 |
| 4.2.1.2 | p__Firmicutes | 98 | 116 | 126 | 96 | 156 | 108 |
| 4.2.1.2 | p__Acidobacteria | 46 | 30 | 36 | 70 | 60 | 32 |
| 4.2.1.2 | p__Gemmatimonadetes | 2 | 0 | 8 | 2 | 0 | 4 |
| 4.2.1.2 | p__Nitrospirae | 46 | 70 | 58 | 40 | 32 | 40 |
| 4.2.1.2 | p__unclassified_d__Bacteria | 4 | 4 | 0 | 0 | 0 | 8 |
| 4.2.1.2 | p__Cyanobacteria | 32 | 46 | 40 | 34 | 34 | 28 |
| 4.2.1.2 | p__Planctomycetes | 0 | 0 | 0 | 0 | 0 | 0 |
| 4.2.1.2 | p__Deinococcus-Thermus | 2 | 2 | 4 | 2 | 6 | 2 |
| 4.2.1.2 | p__Verrucomicrobia | 34 | 46 | 40 | 76 | 100 | 56 |
| 4.2.1.2 | p__Armatimonadetes | 22 | 30 | 18 | 8 | 10 | 8 |
| 4.2.1.2 | p__Candidatus_Tectomicrobia | 0 | 0 | 0 | 0 | 0 | 0 |
| 4.2.1.2 | p__Candidatus_Rokubacteria | 2 | 12 | 4 | 6 | 0 | 4 |
| 4.2.1.2 | p__candidate_division_NC10 | 0 | 0 | 0 | 0 | 0 | 0 |
| 4.2.1.2 | p__candidate_division_Zixibacteria | 0 | 0 | 0 | 0 | 0 | 0 |
| 4.2.1.2 | p__Spirochaetes | 0 | 0 | 0 | 0 | 0 | 0 |
| 4.2.1.2 | p__Candidatus_Acetothermia | 0 | 0 | 0 | 0 | 0 | 0 |
| 4.2.1.2 | p__Candidatus_Dadabacteria | 0 | 0 | 0 | 0 | 0 | 0 |
| 4.2.1.2 | p__Chlorobi | 0 | 0 | 0 | 0 | 0 | 0 |
| 4.2.1.2 | p__Candidatus_Omnitrophica | 0 | 0 | 0 | 0 | 0 | 0 |
| 4.2.1.2 | p__Aquificae | 0 | 0 | 0 | 0 | 0 | 0 |
| 4.2.1.2 | p__Thermodesulfobacteria | 0 | 0 | 0 | 0 | 0 | 0 |
| 4.2.1.2 | p__Latescibacteria | 0 | 0 | 0 | 0 | 0 | 0 |
| 4.2.1.2 | p__Candidatus_Gottesmanbacteria | 0 | 0 | 0 | 0 | 0 | 0 |
| 4.2.1.2 | p__Synergistetes | 0 | 0 | 0 | 0 | 0 | 0 |
| 4.2.1.2 | p__Candidatus_Woesebacteria | 0 | 0 | 0 | 0 | 0 | 0 |
| 4.2.1.2 | p__Thermotogae | 0 | 0 | 0 | 0 | 0 | 0 |
| 4.2.1.2 | others | 2 | 4 | 14 | 2 | 2 | 6 |
| 2.7.9.2 | p__Proteobacteria | 1280 | 1354 | 1026 | 4602 | 5096 | 4300 |
| 2.7.9.2 | p__Actinobacteria | 1994 | 2110 | 2306 | 1932 | 2184 | 1762 |
| 2.7.9.2 | p__Bacteroidetes | 34 | 16 | 34 | 44 | 38 | 26 |
| 2.7.9.2 | p__Chloroflexi | 688 | 622 | 624 | 360 | 466 | 440 |
| 2.7.9.2 | p__Firmicutes | 14 | 42 | 26 | 48 | 36 | 42 |
| 2.7.9.2 | p__Acidobacteria | 10 | 12 | 16 | 6 | 0 | 14 |
| 2.7.9.2 | p__Gemmatimonadetes | 0 | 4 | 0 | 2 | 0 | 0 |
| 2.7.9.2 | p__Nitrospirae | 78 | 92 | 110 | 60 | 82 | 64 |
| 2.7.9.2 | p__unclassified_d__Bacteria | 0 | 0 | 0 | 0 | 0 | 0 |
| 2.7.9.2 | p__Cyanobacteria | 0 | 0 | 0 | 2 | 0 | 2 |
| 2.7.9.2 | p__Planctomycetes | 2 | 12 | 2 | 10 | 0 | 6 |
| 2.7.9.2 | p__Deinococcus-Thermus | 0 | 0 | 0 | 2 | 0 | 0 |
| 2.7.9.2 | p__Verrucomicrobia | 0 | 0 | 0 | 0 | 0 | 0 |
| 2.7.9.2 | p__Armatimonadetes | 20 | 8 | 8 | 4 | 20 | 4 |
| 2.7.9.2 | p__Candidatus_Tectomicrobia | 0 | 0 | 2 | 2 | 0 | 0 |
| 2.7.9.2 | p__Candidatus_Rokubacteria | 0 | 0 | 0 | 0 | 0 | 0 |
| 2.7.9.2 | p__candidate_division_NC10 | 0 | 0 | 0 | 0 | 0 | 0 |
| 2.7.9.2 | p__candidate_division_Zixibacteria | 0 | 0 | 0 | 0 | 0 | 0 |
| 2.7.9.2 | p__Spirochaetes | 0 | 0 | 0 | 0 | 0 | 0 |
| 2.7.9.2 | p__Candidatus_Acetothermia | 0 | 0 | 0 | 0 | 0 | 0 |
| 2.7.9.2 | p__Candidatus_Dadabacteria | 0 | 0 | 0 | 0 | 0 | 0 |
| 2.7.9.2 | p__Chlorobi | 0 | 0 | 0 | 0 | 0 | 0 |
| 2.7.9.2 | p__Candidatus_Omnitrophica | 0 | 0 | 0 | 0 | 0 | 0 |
| 2.7.9.2 | p__Aquificae | 0 | 0 | 0 | 0 | 0 | 0 |
| 2.7.9.2 | p__Thermodesulfobacteria | 0 | 0 | 0 | 0 | 0 | 0 |
| 2.7.9.2 | p__Latescibacteria | 0 | 0 | 0 | 0 | 0 | 0 |
| 2.7.9.2 | p__Candidatus_Gottesmanbacteria | 0 | 0 | 0 | 0 | 0 | 0 |
| 2.7.9.2 | p__Synergistetes | 0 | 0 | 0 | 0 | 0 | 0 |
| 2.7.9.2 | p__Candidatus_Woesebacteria | 0 | 0 | 0 | 0 | 0 | 0 |
| 2.7.9.2 | p__Thermotogae | 0 | 0 | 0 | 0 | 0 | 0 |
| 2.7.9.2 | others | 0 | 0 | 0 | 0 | 0 | 0 |
| 4.1.1.31 | p__Proteobacteria | 1516 | 1446 | 946 | 5170 | 5582 | 4818 |
| 4.1.1.31 | p__Actinobacteria | 842 | 856 | 1068 | 810 | 976 | 892 |
| 4.1.1.31 | p__Bacteroidetes | 20 | 32 | 18 | 150 | 160 | 76 |
| 4.1.1.31 | p__Chloroflexi | 102 | 106 | 118 | 46 | 88 | 68 |
| 4.1.1.31 | p__Firmicutes | 154 | 132 | 156 | 88 | 112 | 94 |
| 4.1.1.31 | p__Acidobacteria | 76 | 68 | 46 | 52 | 80 | 60 |
| 4.1.1.31 | p__Gemmatimonadetes | 16 | 20 | 18 | 12 | 28 | 14 |
| 4.1.1.31 | p__Nitrospirae | 0 | 0 | 0 | 0 | 0 | 0 |
| 4.1.1.31 | p__unclassified_d__Bacteria | 34 | 30 | 18 | 14 | 0 | 20 |
| 4.1.1.31 | p__Cyanobacteria | 0 | 0 | 0 | 0 | 0 | 0 |
| 4.1.1.31 | p__Planctomycetes | 0 | 0 | 0 | 0 | 0 | 0 |
| 4.1.1.31 | p__Deinococcus-Thermus | 0 | 0 | 0 | 0 | 0 | 0 |
| 4.1.1.31 | p__Verrucomicrobia | 0 | 0 | 0 | 24 | 16 | 6 |
| 4.1.1.31 | p__Armatimonadetes | 0 | 0 | 0 | 0 | 0 | 0 |
| 4.1.1.31 | p__Candidatus_Tectomicrobia | 12 | 16 | 18 | 4 | 8 | 12 |
| 4.1.1.31 | p__Candidatus_Rokubacteria | 0 | 0 | 0 | 0 | 0 | 0 |
| 4.1.1.31 | p__candidate_division_NC10 | 0 | 0 | 0 | 0 | 0 | 0 |
| 4.1.1.31 | p__candidate_division_Zixibacteria | 0 | 0 | 0 | 0 | 0 | 0 |
| 4.1.1.31 | p__Spirochaetes | 0 | 0 | 0 | 0 | 0 | 0 |
| 4.1.1.31 | p__Candidatus_Acetothermia | 0 | 0 | 0 | 0 | 0 | 0 |
| 4.1.1.31 | p__Candidatus_Dadabacteria | 0 | 0 | 0 | 0 | 0 | 0 |
| 4.1.1.31 | p__Chlorobi | 0 | 0 | 0 | 0 | 0 | 0 |
| 4.1.1.31 | p__Candidatus_Omnitrophica | 0 | 0 | 0 | 0 | 0 | 0 |
| 4.1.1.31 | p__Aquificae | 0 | 0 | 0 | 0 | 0 | 0 |
| 4.1.1.31 | p__Thermodesulfobacteria | 0 | 0 | 0 | 0 | 0 | 0 |
| 4.1.1.31 | p__Latescibacteria | 0 | 0 | 0 | 0 | 0 | 0 |
| 4.1.1.31 | p__Candidatus_Gottesmanbacteria | 0 | 0 | 0 | 0 | 0 | 0 |
| 4.1.1.31 | p__Synergistetes | 0 | 0 | 0 | 0 | 0 | 0 |
| 4.1.1.31 | p__Candidatus_Woesebacteria | 0 | 0 | 0 | 0 | 0 | 0 |
| 4.1.1.31 | p__Thermotogae | 0 | 0 | 0 | 0 | 0 | 0 |
| 4.1.1.31 | others | 0 | 0 | 0 | 0 | 0 | 0 |
| 1.2.7.1 | p__Proteobacteria | 4 | 8 | 2 | 1036 | 1318 | 1182 |
| 1.2.7.1 | p__Actinobacteria | 298 | 352 | 342 | 266 | 274 | 298 |
| 1.2.7.1 | p__Bacteroidetes | 10 | 24 | 12 | 44 | 26 | 34 |
| 1.2.7.1 | p__Chloroflexi | 0 | 0 | 0 | 0 | 0 | 0 |
| 1.2.7.1 | p__Firmicutes | 8 | 116 | 0 | 132 | 74 | 62 |
| 1.2.7.1 | p__Acidobacteria | 2 | 0 | 0 | 0 | 0 | 0 |
| 1.2.7.1 | p__Gemmatimonadetes | 0 | 0 | 0 | 0 | 0 | 0 |
| 1.2.7.1 | p__Nitrospirae | 342 | 420 | 384 | 270 | 294 | 208 |
| 1.2.7.1 | p__unclassified_d__Bacteria | 0 | 0 | 0 | 2 | 0 | 0 |
| 1.2.7.1 | p__Cyanobacteria | 0 | 0 | 0 | 0 | 0 | 0 |
| 1.2.7.1 | p__Planctomycetes | 14 | 6 | 14 | 4 | 8 | 14 |
| 1.2.7.1 | p__Deinococcus-Thermus | 0 | 0 | 0 | 0 | 0 | 0 |
| 1.2.7.1 | p__Verrucomicrobia | 0 | 0 | 0 | 0 | 0 | 0 |
| 1.2.7.1 | p__Armatimonadetes | 0 | 2 | 0 | 0 | 0 | 0 |
| 1.2.7.1 | p__Candidatus_Tectomicrobia | 0 | 0 | 0 | 0 | 0 | 0 |
| 1.2.7.1 | p__Candidatus_Rokubacteria | 0 | 0 | 0 | 0 | 0 | 0 |
| 1.2.7.1 | p__candidate_division_NC10 | 10 | 8 | 8 | 10 | 24 | 6 |
| 1.2.7.1 | p__candidate_division_Zixibacteria | 0 | 0 | 0 | 0 | 0 | 0 |
| 1.2.7.1 | p__Spirochaetes | 0 | 0 | 0 | 0 | 0 | 0 |
| 1.2.7.1 | p__Candidatus_Acetothermia | 0 | 0 | 0 | 0 | 0 | 0 |
| 1.2.7.1 | p__Candidatus_Dadabacteria | 0 | 0 | 0 | 0 | 0 | 0 |
| 1.2.7.1 | p__Chlorobi | 0 | 0 | 0 | 0 | 0 | 0 |
| 1.2.7.1 | p__Candidatus_Omnitrophica | 0 | 0 | 0 | 0 | 0 | 0 |
| 1.2.7.1 | p__Aquificae | 0 | 0 | 0 | 0 | 0 | 0 |
| 1.2.7.1 | p__Thermodesulfobacteria | 0 | 0 | 0 | 0 | 0 | 0 |
| 1.2.7.1 | p__Latescibacteria | 0 | 0 | 0 | 0 | 0 | 0 |
| 1.2.7.1 | p__Candidatus_Gottesmanbacteria | 0 | 0 | 0 | 0 | 0 | 0 |
| 1.2.7.1 | p__Synergistetes | 0 | 0 | 0 | 0 | 0 | 0 |
| 1.2.7.1 | p__Candidatus_Woesebacteria | 0 | 0 | 0 | 0 | 0 | 0 |
| 1.2.7.1 | p__Thermotogae | 0 | 0 | 0 | 0 | 0 | 0 |
| 1.2.7.1 | others | 0 | 0 | 0 | 0 | 0 | 0 |

| Table S5. Distribution of bacteria communities at order level contributed to the significantly increased abundance of key enzymes in carbon fixation pathways. | | | | | | | |
| --- | --- | --- | --- | --- | --- | --- | --- |
| Function | Taxon | T2Ck_a | T2Ck_b | T2Ck_c | T2B300_a | T2B300_b | T2B300_c |
| 6.4.1.2 | o__Burkholderiales | 344 | 310 | 144 | 1626 | 1646 | 1516 |
| 6.4.1.2 | o__Pseudomonadales | 442 | 362 | 30 | 1574 | 1870 | 1474 |
| 6.4.1.2 | o__Xanthomonadales | 336 | 326 | 296 | 882 | 1086 | 890 |
| 6.4.1.2 | o__Solirubrobacterales | 628 | 622 | 828 | 528 | 668 | 490 |
| 6.4.1.2 | o__Rhizobiales | 372 | 388 | 272 | 1210 | 1424 | 1136 |
| 6.4.1.2 | o__Enterobacteriales | 0 | 2 | 0 | 848 | 1030 | 990 |
| 6.4.1.2 | o__Propionibacteriales | 622 | 630 | 830 | 526 | 654 | 562 |
| 6.4.1.2 | o__Sphingomonadales | 362 | 358 | 302 | 444 | 486 | 412 |
| 6.4.1.2 | o__Micrococcales | 264 | 258 | 264 | 292 | 356 | 240 |
| 6.4.1.2 | o__Streptomycetales | 190 | 198 | 272 | 208 | 236 | 160 |
| 6.4.1.2 | o__Rhodospirillales | 36 | 56 | 32 | 212 | 222 | 264 |
| 6.4.1.2 | o__Rubrobacterales | 614 | 720 | 764 | 404 | 562 | 340 |
| 6.4.1.2 | o__Flavobacteriales | 140 | 194 | 72 | 306 | 288 | 150 |
| 6.4.1.2 | o__Neisseriales | 82 | 26 | 2 | 788 | 774 | 702 |
| 6.4.1.2 | o__Cellvibrionales | 76 | 126 | 90 | 322 | 282 | 210 |
| 6.4.1.2 | o__Pseudonocardiales | 44 | 74 | 40 | 38 | 42 | 38 |
| 6.4.1.2 | o__Corynebacteriales | 62 | 62 | 48 | 44 | 54 | 52 |
| 6.4.1.2 | o__Streptosporangiales | 2 | 20 | 22 | 20 | 16 | 22 |
| 6.4.1.2 | o__Gemmatimonadales | 204 | 272 | 240 | 142 | 180 | 192 |
| 6.4.1.2 | o__unclassified_p__Chloroflexi | 258 | 254 | 290 | 194 | 210 | 204 |
| 6.4.1.2 | o__Nitrospirales | 88 | 162 | 154 | 76 | 128 | 92 |
| 6.4.1.2 | o__Micromonosporales | 8 | 12 | 8 | 46 | 20 | 26 |
| 6.4.1.2 | o__Myxococcales | 24 | 72 | 4 | 4 | 6 | 2 |
| 6.4.1.2 | o__Gaiellales | 0 | 0 | 0 | 0 | 0 | 0 |
| 6.4.1.2 | o__unclassified_d__Bacteria | 32 | 30 | 30 | 22 | 52 | 42 |
| 6.4.1.2 | o__Clostridiales | 22 | 16 | 16 | 68 | 100 | 62 |
| 6.4.1.2 | o__unclassified_c__Actinobacteria | 106 | 106 | 124 | 100 | 92 | 98 |
| 6.4.1.2 | o__Aeromonadales | 0 | 12 | 0 | 72 | 184 | 94 |
| 6.4.1.2 | o__Geodermatophilales | 92 | 62 | 80 | 46 | 104 | 50 |
| 6.4.1.2 | o__unclassified_p__Acidobacteria | 202 | 222 | 216 | 124 | 120 | 134 |
| 6.4.1.2 | others | 1236 | 1338 | 1442 | 1384 | 1474 | 1296 |
| 4.2.1.2 | o__Burkholderiales | 292 | 314 | 130 | 1100 | 1260 | 1020 |
| 4.2.1.2 | o__Pseudomonadales | 270 | 230 | 12 | 1148 | 1278 | 1136 |
| 4.2.1.2 | o__Xanthomonadales | 234 | 198 | 186 | 842 | 942 | 794 |
| 4.2.1.2 | o__Solirubrobacterales | 684 | 772 | 830 | 676 | 756 | 638 |
| 4.2.1.2 | o__Rhizobiales | 174 | 164 | 124 | 238 | 278 | 220 |
| 4.2.1.2 | o__Enterobacteriales | 2 | 6 | 6 | 1036 | 1232 | 1470 |
| 4.2.1.2 | o__Propionibacteriales | 386 | 386 | 476 | 250 | 306 | 244 |
| 4.2.1.2 | o__Sphingomonadales | 186 | 250 | 164 | 300 | 426 | 334 |
| 4.2.1.2 | o__Micrococcales | 86 | 76 | 96 | 110 | 106 | 96 |
| 4.2.1.2 | o__Streptomycetales | 76 | 62 | 62 | 52 | 64 | 38 |
| 4.2.1.2 | o__Rhodospirillales | 26 | 14 | 10 | 204 | 262 | 228 |
| 4.2.1.2 | o__Rubrobacterales | 186 | 202 | 182 | 78 | 92 | 116 |
| 4.2.1.2 | o__Flavobacteriales | 110 | 206 | 62 | 230 | 204 | 140 |
| 4.2.1.2 | o__Neisseriales | 42 | 8 | 0 | 244 | 216 | 176 |
| 4.2.1.2 | o__Cellvibrionales | 94 | 90 | 50 | 204 | 154 | 146 |
| 4.2.1.2 | o__Pseudonocardiales | 4 | 16 | 4 | 2 | 10 | 8 |
| 4.2.1.2 | o__Corynebacteriales | 26 | 16 | 32 | 18 | 24 | 16 |
| 4.2.1.2 | o__Streptosporangiales | 8 | 10 | 20 | 4 | 4 | 12 |
| 4.2.1.2 | o__Gemmatimonadales | 2 | 0 | 8 | 2 | 0 | 4 |
| 4.2.1.2 | o__unclassified_p__Chloroflexi | 0 | 0 | 0 | 0 | 0 | 0 |
| 4.2.1.2 | o__Nitrospirales | 46 | 70 | 58 | 40 | 32 | 40 |
| 4.2.1.2 | o__Micromonosporales | 10 | 6 | 8 | 20 | 32 | 14 |
| 4.2.1.2 | o__Myxococcales | 10 | 128 | 22 | 26 | 16 | 16 |
| 4.2.1.2 | o__Gaiellales | 0 | 0 | 0 | 0 | 0 | 0 |
| 4.2.1.2 | o__unclassified_d__Bacteria | 4 | 4 | 0 | 0 | 0 | 8 |
| 4.2.1.2 | o__Clostridiales | 46 | 56 | 54 | 62 | 82 | 66 |
| 4.2.1.2 | o__unclassified_c__Actinobacteria | 108 | 118 | 64 | 64 | 118 | 56 |
| 4.2.1.2 | o__Aeromonadales | 0 | 4 | 0 | 44 | 126 | 98 |
| 4.2.1.2 | o__Geodermatophilales | 12 | 18 | 12 | 2 | 10 | 12 |
| 4.2.1.2 | o__unclassified_p__Acidobacteria | 0 | 0 | 0 | 0 | 0 | 0 |
| 4.2.1.2 | others | 470 | 566 | 518 | 520 | 644 | 482 |
| 2.7.9.2 | o__Burkholderiales | 536 | 584 | 526 | 1382 | 1554 | 1260 |
| 2.7.9.2 | o__Pseudomonadales | 220 | 216 | 44 | 838 | 1078 | 776 |
| 2.7.9.2 | o__Xanthomonadales | 232 | 234 | 222 | 708 | 756 | 678 |
| 2.7.9.2 | o__Solirubrobacterales | 476 | 556 | 608 | 442 | 450 | 358 |
| 2.7.9.2 | o__Rhizobiales | 0 | 0 | 0 | 0 | 0 | 0 |
| 2.7.9.2 | o__Enterobacteriales | 0 | 0 | 0 | 568 | 624 | 618 |
| 2.7.9.2 | o__Propionibacteriales | 54 | 40 | 46 | 68 | 62 | 62 |
| 2.7.9.2 | o__Sphingomonadales | 2 | 0 | 0 | 12 | 0 | 0 |
| 2.7.9.2 | o__Micrococcales | 612 | 566 | 578 | 552 | 598 | 464 |
| 2.7.9.2 | o__Streptomycetales | 82 | 74 | 88 | 66 | 86 | 76 |
| 2.7.9.2 | o__Rhodospirillales | 2 | 0 | 2 | 158 | 154 | 174 |
| 2.7.9.2 | o__Rubrobacterales | 6 | 22 | 16 | 22 | 6 | 16 |
| 2.7.9.2 | o__Flavobacteriales | 8 | 2 | 0 | 26 | 34 | 8 |
| 2.7.9.2 | o__Neisseriales | 68 | 34 | 34 | 530 | 546 | 456 |
| 2.7.9.2 | o__Cellvibrionales | 22 | 22 | 28 | 8 | 12 | 14 |
| 2.7.9.2 | o__Pseudonocardiales | 344 | 358 | 448 | 308 | 426 | 324 |
| 2.7.9.2 | o__Corynebacteriales | 16 | 38 | 20 | 24 | 42 | 22 |
| 2.7.9.2 | o__Streptosporangiales | 144 | 178 | 148 | 122 | 150 | 136 |
| 2.7.9.2 | o__Gemmatimonadales | 0 | 4 | 0 | 2 | 0 | 0 |
| 2.7.9.2 | o__unclassified_p__Chloroflexi | 12 | 10 | 20 | 10 | 10 | 14 |
| 2.7.9.2 | o__Nitrospirales | 78 | 92 | 110 | 60 | 82 | 64 |
| 2.7.9.2 | o__Micromonosporales | 0 | 0 | 4 | 2 | 2 | 0 |
| 2.7.9.2 | o__Myxococcales | 8 | 40 | 16 | 4 | 2 | 4 |
| 2.7.9.2 | o__Gaiellales | 0 | 0 | 0 | 0 | 0 | 0 |
| 2.7.9.2 | o__unclassified_d__Bacteria | 0 | 0 | 0 | 0 | 0 | 0 |
| 2.7.9.2 | o__Clostridiales | 10 | 24 | 22 | 24 | 24 | 22 |
| 2.7.9.2 | o__unclassified_c__Actinobacteria | 16 | 16 | 10 | 10 | 14 | 4 |
| 2.7.9.2 | o__Aeromonadales | 0 | 0 | 0 | 48 | 106 | 50 |
| 2.7.9.2 | o__Geodermatophilales | 146 | 142 | 214 | 184 | 212 | 184 |
| 2.7.9.2 | o__unclassified_p__Acidobacteria | 10 | 12 | 16 | 6 | 0 | 14 |
| 2.7.9.2 | others | 1016 | 1008 | 934 | 890 | 892 | 862 |
| 4.1.1.31 | o__Burkholderiales | 338 | 284 | 198 | 1198 | 1328 | 1086 |
| 4.1.1.31 | o__Pseudomonadales | 248 | 204 | 4 | 942 | 974 | 862 |
| 4.1.1.31 | o__Xanthomonadales | 228 | 230 | 184 | 842 | 978 | 868 |
| 4.1.1.31 | o__Solirubrobacterales | 126 | 154 | 172 | 128 | 158 | 146 |
| 4.1.1.31 | o__Rhizobiales | 140 | 154 | 92 | 84 | 168 | 84 |
| 4.1.1.31 | o__Enterobacteriales | 2 | 0 | 0 | 666 | 638 | 742 |
| 4.1.1.31 | o__Propionibacteriales | 104 | 110 | 140 | 44 | 46 | 56 |
| 4.1.1.31 | o__Sphingomonadales | 154 | 186 | 216 | 338 | 360 | 298 |
| 4.1.1.31 | o__Micrococcales | 120 | 118 | 112 | 124 | 144 | 180 |
| 4.1.1.31 | o__Streptomycetales | 296 | 276 | 340 | 272 | 330 | 264 |
| 4.1.1.31 | o__Rhodospirillales | 30 | 22 | 14 | 150 | 230 | 160 |
| 4.1.1.31 | o__Rubrobacterales | 0 | 0 | 0 | 0 | 0 | 0 |
| 4.1.1.31 | o__Flavobacteriales | 18 | 20 | 10 | 124 | 102 | 54 |
| 4.1.1.31 | o__Neisseriales | 26 | 8 | 0 | 362 | 268 | 258 |
| 4.1.1.31 | o__Cellvibrionales | 38 | 56 | 42 | 224 | 200 | 164 |
| 4.1.1.31 | o__Pseudonocardiales | 0 | 0 | 0 | 0 | 0 | 0 |
| 4.1.1.31 | o__Corynebacteriales | 26 | 14 | 10 | 4 | 14 | 14 |
| 4.1.1.31 | o__Streptosporangiales | 100 | 110 | 188 | 182 | 220 | 156 |
| 4.1.1.31 | o__Gemmatimonadales | 16 | 20 | 18 | 12 | 28 | 14 |
| 4.1.1.31 | o__unclassified_p__Chloroflexi | 102 | 106 | 118 | 46 | 88 | 68 |
| 4.1.1.31 | o__Nitrospirales | 0 | 0 | 0 | 0 | 0 | 0 |
| 4.1.1.31 | o__Micromonosporales | 8 | 4 | 20 | 14 | 16 | 20 |
| 4.1.1.31 | o__Myxococcales | 14 | 80 | 10 | 8 | 6 | 2 |
| 4.1.1.31 | o__Gaiellales | 0 | 0 | 0 | 0 | 0 | 0 |
| 4.1.1.31 | o__unclassified_d__Bacteria | 34 | 30 | 18 | 14 | 0 | 20 |
| 4.1.1.31 | o__Clostridiales | 0 | 0 | 0 | 0 | 0 | 0 |
| 4.1.1.31 | o__unclassified_c__Actinobacteria | 10 | 6 | 6 | 0 | 2 | 4 |
| 4.1.1.31 | o__Aeromonadales | 10 | 2 | 0 | 64 | 134 | 52 |
| 4.1.1.31 | o__Geodermatophilales | 28 | 44 | 54 | 26 | 24 | 36 |
| 4.1.1.31 | o__unclassified_p__Acidobacteria | 76 | 68 | 46 | 52 | 80 | 60 |
| 4.1.1.31 | others | 480 | 400 | 394 | 450 | 514 | 392 |
| 1.2.7.1 | o__Burkholderiales | 0 | 0 | 0 | 0 | 0 | 0 |
| 1.2.7.1 | o__Pseudomonadales | 0 | 0 | 0 | 0 | 0 | 0 |
| 1.2.7.1 | o__Xanthomonadales | 0 | 0 | 0 | 0 | 0 | 0 |
| 1.2.7.1 | o__Solirubrobacterales | 0 | 0 | 0 | 0 | 0 | 0 |
| 1.2.7.1 | o__Rhizobiales | 0 | 0 | 0 | 0 | 0 | 0 |
| 1.2.7.1 | o__Enterobacteriales | 0 | 2 | 0 | 778 | 826 | 842 |
| 1.2.7.1 | o__Propionibacteriales | 20 | 14 | 14 | 74 | 66 | 60 |
| 1.2.7.1 | o__Sphingomonadales | 0 | 0 | 0 | 0 | 0 | 0 |
| 1.2.7.1 | o__Micrococcales | 14 | 6 | 6 | 26 | 24 | 8 |
| 1.2.7.1 | o__Streptomycetales | 0 | 0 | 0 | 0 | 0 | 0 |
| 1.2.7.1 | o__Rhodospirillales | 2 | 4 | 2 | 126 | 152 | 146 |
| 1.2.7.1 | o__Rubrobacterales | 264 | 330 | 310 | 164 | 182 | 230 |
| 1.2.7.1 | o__Flavobacteriales | 0 | 8 | 0 | 22 | 8 | 6 |
| 1.2.7.1 | o__Neisseriales | 0 | 0 | 0 | 0 | 0 | 0 |
| 1.2.7.1 | o__Cellvibrionales | 0 | 0 | 0 | 0 | 0 | 0 |
| 1.2.7.1 | o__Pseudonocardiales | 0 | 0 | 0 | 0 | 0 | 0 |
| 1.2.7.1 | o__Corynebacteriales | 0 | 0 | 0 | 0 | 0 | 0 |
| 1.2.7.1 | o__Streptosporangiales | 0 | 0 | 0 | 0 | 0 | 0 |
| 1.2.7.1 | o__Gemmatimonadales | 0 | 0 | 0 | 0 | 0 | 0 |
| 1.2.7.1 | o__unclassified_p__Chloroflexi | 0 | 0 | 0 | 0 | 0 | 0 |
| 1.2.7.1 | o__Nitrospirales | 342 | 420 | 384 | 270 | 294 | 208 |
| 1.2.7.1 | o__Micromonosporales | 0 | 0 | 0 | 0 | 0 | 0 |
| 1.2.7.1 | o__Myxococcales | 0 | 0 | 0 | 0 | 0 | 0 |
| 1.2.7.1 | o__Gaiellales | 0 | 0 | 0 | 0 | 0 | 0 |
| 1.2.7.1 | o__unclassified_d__Bacteria | 0 | 0 | 0 | 2 | 0 | 0 |
| 1.2.7.1 | o__Clostridiales | 8 | 8 | 0 | 106 | 64 | 52 |
| 1.2.7.1 | o__unclassified_c__Actinobacteria | 0 | 0 | 0 | 0 | 0 | 0 |
| 1.2.7.1 | o__Aeromonadales | 2 | 2 | 0 | 132 | 340 | 194 |
| 1.2.7.1 | o__Geodermatophilales | 0 | 0 | 0 | 0 | 0 | 0 |
| 1.2.7.1 | o__unclassified_p__Acidobacteria | 0 | 0 | 0 | 0 | 0 | 0 |
| 1.2.7.1 | others | 36 | 142 | 46 | 64 | 62 | 58 |

| Table S6. Distribution of bacteria communities at genera level contributed to the significantly increased abundance of key enzymes in carbon fixation pathways. | | | | | | | |
| --- | --- | --- | --- | --- | --- | --- | --- |
| Function | Taxon | T2Ck_a | T2Ck_b | T2Ck_c | T2B300_a | T2B300_b | T2B300_c |
| 6.4.1.2 | g__Pseudomonas | 434 | 352 | 30 | 1326 | 1540 | 1292 |
| 6.4.1.2 | g__Lelliottia | 0 | 0 | 0 | 490 | 662 | 566 |
| 6.4.1.2 | g__Nocardioides | 554 | 490 | 680 | 406 | 538 | 460 |
| 6.4.1.2 | g__Pseudoxanthomonas | 138 | 104 | 92 | 384 | 480 | 416 |
| 6.4.1.2 | g__Achromobacter | 72 | 96 | 34 | 402 | 458 | 334 |
| 6.4.1.2 | g__Solirubrobacter | 344 | 398 | 466 | 324 | 398 | 304 |
| 6.4.1.2 | g__Delftia | 0 | 0 | 0 | 602 | 552 | 594 |
| 6.4.1.2 | g__Acidovorax | 154 | 110 | 38 | 328 | 314 | 334 |
| 6.4.1.2 | g__Streptomyces | 190 | 194 | 256 | 204 | 230 | 158 |
| 6.4.1.2 | g__Conexibacter | 276 | 220 | 344 | 202 | 246 | 176 |
| 6.4.1.2 | g__Azospirillum | 14 | 10 | 2 | 194 | 194 | 240 |
| 6.4.1.2 | g__Rubrobacter | 614 | 720 | 764 | 404 | 562 | 340 |
| 6.4.1.2 | g__Lysobacter | 136 | 120 | 138 | 222 | 332 | 244 |
| 6.4.1.2 | g__Flavobacterium | 134 | 178 | 68 | 292 | 262 | 134 |
| 6.4.1.2 | g__Rhizobium | 68 | 54 | 48 | 274 | 348 | 254 |
| 6.4.1.2 | g__Agrobacterium | 18 | 26 | 10 | 464 | 404 | 416 |
| 6.4.1.2 | g__Vogesella | 82 | 26 | 2 | 788 | 774 | 702 |
| 6.4.1.2 | g__Sphingomonas | 214 | 216 | 188 | 190 | 178 | 188 |
| 6.4.1.2 | g__Cellvibrio | 8 | 38 | 26 | 288 | 246 | 188 |
| 6.4.1.2 | g__unclassified_o__Solirubrobacterales | 8 | 4 | 18 | 2 | 24 | 6 |
| 6.4.1.2 | g__Arthrobacter | 198 | 136 | 184 | 156 | 178 | 148 |
| 6.4.1.2 | g__Sphingobium | 94 | 92 | 76 | 146 | 170 | 114 |
| 6.4.1.2 | g__Sphingopyxis | 52 | 48 | 38 | 86 | 90 | 92 |
| 6.4.1.2 | g__Variovorax | 20 | 4 | 14 | 48 | 48 | 52 |
| 6.4.1.2 | g__Nitrospira | 78 | 156 | 132 | 68 | 120 | 78 |
| 6.4.1.2 | g__unclassified_p__Chloroflexi | 258 | 254 | 290 | 194 | 210 | 204 |
| 6.4.1.2 | g__Gaiella | 0 | 0 | 0 | 0 | 0 | 0 |
| 6.4.1.2 | g__Enterobacter | 0 | 2 | 0 | 248 | 290 | 328 |
| 6.4.1.2 | g__Phyllobacterium | 36 | 42 | 46 | 78 | 122 | 88 |
| 6.4.1.2 | g__Acinetobacter | 0 | 4 | 0 | 228 | 298 | 148 |
| 6.4.1.2 | others | 2694 | 3196 | 2928 | 3512 | 4098 | 3342 |
| 4.2.1.2 | g__Pseudomonas | 266 | 202 | 12 | 982 | 1034 | 992 |
| 4.2.1.2 | g__Lelliottia | 0 | 4 | 0 | 896 | 1150 | 1252 |
| 4.2.1.2 | g__Nocardioides | 350 | 344 | 454 | 224 | 290 | 232 |
| 4.2.1.2 | g__Pseudoxanthomonas | 120 | 100 | 84 | 528 | 596 | 598 |
| 4.2.1.2 | g__Achromobacter | 84 | 124 | 16 | 376 | 420 | 372 |
| 4.2.1.2 | g__Solirubrobacter | 364 | 430 | 504 | 360 | 406 | 342 |
| 4.2.1.2 | g__Delftia | 0 | 0 | 0 | 294 | 254 | 256 |
| 4.2.1.2 | g__Acidovorax | 72 | 56 | 28 | 132 | 158 | 144 |
| 4.2.1.2 | g__Streptomyces | 70 | 54 | 54 | 40 | 54 | 30 |
| 4.2.1.2 | g__Conexibacter | 320 | 336 | 322 | 316 | 346 | 296 |
| 4.2.1.2 | g__Azospirillum | 10 | 4 | 4 | 194 | 246 | 210 |
| 4.2.1.2 | g__Rubrobacter | 186 | 202 | 182 | 78 | 92 | 116 |
| 4.2.1.2 | g__Lysobacter | 66 | 52 | 82 | 160 | 176 | 96 |
| 4.2.1.2 | g__Flavobacterium | 110 | 204 | 62 | 226 | 202 | 140 |
| 4.2.1.2 | g__Rhizobium | 20 | 10 | 14 | 20 | 48 | 40 |
| 4.2.1.2 | g__Agrobacterium | 0 | 2 | 0 | 16 | 20 | 14 |
| 4.2.1.2 | g__Vogesella | 22 | 4 | 0 | 194 | 198 | 150 |
| 4.2.1.2 | g__Sphingomonas | 60 | 88 | 58 | 92 | 120 | 94 |
| 4.2.1.2 | g__Cellvibrio | 44 | 46 | 14 | 196 | 144 | 126 |
| 4.2.1.2 | g__unclassified_o__Solirubrobacterales | 0 | 6 | 4 | 0 | 4 | 0 |
| 4.2.1.2 | g__Arthrobacter | 22 | 20 | 12 | 28 | 18 | 32 |
| 4.2.1.2 | g__Sphingobium | 68 | 106 | 58 | 128 | 172 | 140 |
| 4.2.1.2 | g__Sphingopyxis | 50 | 46 | 40 | 56 | 102 | 80 |
| 4.2.1.2 | g__Variovorax | 16 | 44 | 26 | 58 | 46 | 36 |
| 4.2.1.2 | g__Nitrospira | 46 | 70 | 58 | 40 | 32 | 40 |
| 4.2.1.2 | g__unclassified_p__Chloroflexi | 0 | 0 | 0 | 0 | 0 | 0 |
| 4.2.1.2 | g__Gaiella | 0 | 0 | 0 | 0 | 0 | 0 |
| 4.2.1.2 | g__Enterobacter | 0 | 0 | 0 | 26 | 32 | 178 |
| 4.2.1.2 | g__Phyllobacterium | 14 | 18 | 18 | 28 | 32 | 38 |
| 4.2.1.2 | g__Acinetobacter | 0 | 12 | 0 | 134 | 210 | 124 |
| 4.2.1.2 | others | 1214 | 1406 | 1084 | 1694 | 2072 | 1460 |
| 2.7.9.2 | g__Pseudomonas | 214 | 206 | 44 | 716 | 826 | 626 |
| 2.7.9.2 | g__Lelliottia | 0 | 0 | 0 | 460 | 568 | 508 |
| 2.7.9.2 | g__Nocardioides | 6 | 4 | 8 | 16 | 4 | 10 |
| 2.7.9.2 | g__Pseudoxanthomonas | 142 | 120 | 92 | 498 | 480 | 476 |
| 2.7.9.2 | g__Achromobacter | 68 | 84 | 14 | 400 | 470 | 374 |
| 2.7.9.2 | g__Solirubrobacter | 330 | 438 | 444 | 318 | 320 | 256 |
| 2.7.9.2 | g__Delftia | 0 | 0 | 0 | 244 | 194 | 162 |
| 2.7.9.2 | g__Acidovorax | 48 | 46 | 18 | 160 | 176 | 168 |
| 2.7.9.2 | g__Streptomyces | 82 | 74 | 88 | 66 | 86 | 76 |
| 2.7.9.2 | g__Conexibacter | 0 | 0 | 0 | 0 | 0 | 0 |
| 2.7.9.2 | g__Azospirillum | 0 | 0 | 0 | 0 | 0 | 0 |
| 2.7.9.2 | g__Rubrobacter | 6 | 22 | 16 | 22 | 6 | 16 |
| 2.7.9.2 | g__Lysobacter | 28 | 30 | 46 | 28 | 56 | 40 |
| 2.7.9.2 | g__Flavobacterium | 8 | 2 | 0 | 26 | 34 | 8 |
| 2.7.9.2 | g__Rhizobium | 0 | 0 | 0 | 0 | 0 | 0 |
| 2.7.9.2 | g__Agrobacterium | 0 | 0 | 0 | 0 | 0 | 0 |
| 2.7.9.2 | g__Vogesella | 56 | 10 | 0 | 516 | 520 | 444 |
| 2.7.9.2 | g__Sphingomonas | 0 | 0 | 0 | 0 | 0 | 0 |
| 2.7.9.2 | g__Cellvibrio | 0 | 0 | 0 | 0 | 0 | 0 |
| 2.7.9.2 | g__unclassified_o__Solirubrobacterales | 146 | 118 | 164 | 124 | 130 | 102 |
| 2.7.9.2 | g__Arthrobacter | 406 | 422 | 458 | 348 | 372 | 316 |
| 2.7.9.2 | g__Sphingobium | 0 | 0 | 0 | 0 | 0 | 0 |
| 2.7.9.2 | g__Sphingopyxis | 0 | 0 | 0 | 0 | 0 | 0 |
| 2.7.9.2 | g__Variovorax | 334 | 362 | 418 | 336 | 474 | 368 |
| 2.7.9.2 | g__Nitrospira | 78 | 92 | 110 | 60 | 82 | 64 |
| 2.7.9.2 | g__unclassified_p__Chloroflexi | 12 | 10 | 20 | 10 | 10 | 14 |
| 2.7.9.2 | g__Gaiella | 0 | 0 | 0 | 0 | 0 | 0 |
| 2.7.9.2 | g__Enterobacter | 0 | 0 | 0 | 18 | 18 | 90 |
| 2.7.9.2 | g__Phyllobacterium | 0 | 0 | 0 | 0 | 0 | 0 |
| 2.7.9.2 | g__Acinetobacter | 0 | 0 | 0 | 108 | 178 | 130 |
| 2.7.9.2 | others | 2156 | 2232 | 2214 | 2600 | 2918 | 2412 |
| 4.1.1.31 | g__Pseudomonas | 244 | 204 | 4 | 808 | 848 | 738 |
| 4.1.1.31 | g__Lelliottia | 0 | 0 | 0 | 542 | 560 | 610 |
| 4.1.1.31 | g__Nocardioides | 46 | 48 | 112 | 6 | 4 | 8 |
| 4.1.1.31 | g__Pseudoxanthomonas | 132 | 118 | 60 | 476 | 610 | 540 |
| 4.1.1.31 | g__Achromobacter | 60 | 68 | 18 | 344 | 358 | 276 |
| 4.1.1.31 | g__Solirubrobacter | 102 | 124 | 140 | 106 | 134 | 106 |
| 4.1.1.31 | g__Delftia | 0 | 0 | 0 | 240 | 228 | 262 |
| 4.1.1.31 | g__Acidovorax | 56 | 64 | 20 | 218 | 276 | 242 |
| 4.1.1.31 | g__Streptomyces | 290 | 262 | 334 | 268 | 320 | 260 |
| 4.1.1.31 | g__Conexibacter | 18 | 26 | 22 | 8 | 16 | 22 |
| 4.1.1.31 | g__Azospirillum | 2 | 0 | 2 | 132 | 220 | 154 |
| 4.1.1.31 | g__Rubrobacter | 0 | 0 | 0 | 0 | 0 | 0 |
| 4.1.1.31 | g__Lysobacter | 48 | 36 | 48 | 126 | 144 | 118 |
| 4.1.1.31 | g__Flavobacterium | 18 | 20 | 10 | 122 | 102 | 54 |
| 4.1.1.31 | g__Rhizobium | 0 | 0 | 0 | 0 | 0 | 0 |
| 4.1.1.31 | g__Agrobacterium | 0 | 0 | 0 | 0 | 0 | 0 |
| 4.1.1.31 | g__Vogesella | 26 | 8 | 0 | 362 | 268 | 258 |
| 4.1.1.31 | g__Sphingomonas | 104 | 132 | 142 | 208 | 198 | 166 |
| 4.1.1.31 | g__Cellvibrio | 38 | 56 | 42 | 224 | 200 | 164 |
| 4.1.1.31 | g__unclassified_o__Solirubrobacterales | 6 | 4 | 10 | 14 | 8 | 18 |
| 4.1.1.31 | g__Arthrobacter | 100 | 76 | 70 | 78 | 80 | 96 |
| 4.1.1.31 | g__Sphingobium | 8 | 0 | 20 | 10 | 26 | 10 |
| 4.1.1.31 | g__Sphingopyxis | 14 | 28 | 28 | 70 | 58 | 68 |
| 4.1.1.31 | g__Variovorax | 32 | 16 | 16 | 46 | 50 | 38 |
| 4.1.1.31 | g__Nitrospira | 0 | 0 | 0 | 0 | 0 | 0 |
| 4.1.1.31 | g__unclassified_p__Chloroflexi | 102 | 106 | 118 | 46 | 88 | 68 |
| 4.1.1.31 | g__Gaiella | 0 | 0 | 0 | 0 | 0 | 0 |
| 4.1.1.31 | g__Enterobacter | 0 | 0 | 0 | 8 | 10 | 82 |
| 4.1.1.31 | g__Phyllobacterium | 0 | 0 | 0 | 0 | 0 | 0 |
| 4.1.1.31 | g__Acinetobacter | 0 | 0 | 0 | 104 | 108 | 104 |
| 4.1.1.31 | others | 1326 | 1310 | 1190 | 1804 | 2136 | 1598 |
| 1.2.7.1 | g__Pseudomonas | 0 | 0 | 0 | 0 | 0 | 0 |
| 1.2.7.1 | g__Lelliottia | 0 | 2 | 0 | 710 | 752 | 732 |
| 1.2.7.1 | g__Nocardioides | 20 | 14 | 14 | 74 | 66 | 60 |
| 1.2.7.1 | g__Pseudoxanthomonas | 0 | 0 | 0 | 0 | 0 | 0 |
| 1.2.7.1 | g__Achromobacter | 0 | 0 | 0 | 0 | 0 | 0 |
| 1.2.7.1 | g__Solirubrobacter | 0 | 0 | 0 | 0 | 0 | 0 |
| 1.2.7.1 | g__Delftia | 0 | 0 | 0 | 0 | 0 | 0 |
| 1.2.7.1 | g__Acidovorax | 0 | 0 | 0 | 0 | 0 | 0 |
| 1.2.7.1 | g__Streptomyces | 0 | 0 | 0 | 0 | 0 | 0 |
| 1.2.7.1 | g__Conexibacter | 0 | 0 | 0 | 0 | 0 | 0 |
| 1.2.7.1 | g__Azospirillum | 2 | 4 | 2 | 126 | 152 | 146 |
| 1.2.7.1 | g__Rubrobacter | 264 | 330 | 310 | 164 | 182 | 230 |
| 1.2.7.1 | g__Lysobacter | 0 | 0 | 0 | 0 | 0 | 0 |
| 1.2.7.1 | g__Flavobacterium | 0 | 0 | 0 | 0 | 0 | 0 |
| 1.2.7.1 | g__Rhizobium | 0 | 0 | 0 | 0 | 0 | 0 |
| 1.2.7.1 | g__Agrobacterium | 0 | 0 | 0 | 0 | 0 | 0 |
| 1.2.7.1 | g__Vogesella | 0 | 0 | 0 | 0 | 0 | 0 |
| 1.2.7.1 | g__Sphingomonas | 0 | 0 | 0 | 0 | 0 | 0 |
| 1.2.7.1 | g__Cellvibrio | 0 | 0 | 0 | 0 | 0 | 0 |
| 1.2.7.1 | g__unclassified_o__Solirubrobacterales | 0 | 0 | 0 | 0 | 0 | 0 |
| 1.2.7.1 | g__Arthrobacter | 0 | 0 | 0 | 0 | 0 | 0 |
| 1.2.7.1 | g__Sphingobium | 0 | 0 | 0 | 0 | 0 | 0 |
| 1.2.7.1 | g__Sphingopyxis | 0 | 0 | 0 | 0 | 0 | 0 |
| 1.2.7.1 | g__Variovorax | 0 | 0 | 0 | 0 | 0 | 0 |
| 1.2.7.1 | g__Nitrospira | 342 | 420 | 384 | 270 | 294 | 208 |
| 1.2.7.1 | g__unclassified_p__Chloroflexi | 0 | 0 | 0 | 0 | 0 | 0 |
| 1.2.7.1 | g__Gaiella | 0 | 0 | 0 | 0 | 0 | 0 |
| 1.2.7.1 | g__Enterobacter | 0 | 0 | 0 | 16 | 18 | 82 |
| 1.2.7.1 | g__Phyllobacterium | 0 | 0 | 0 | 0 | 0 | 0 |
| 1.2.7.1 | g__Acinetobacter | 0 | 0 | 0 | 0 | 0 | 0 |
| 1.2.7.1 | others | 60 | 166 | 52 | 404 | 554 | 346 |

| Table S7. Distribution of bacteria communities at phyla level contributed to the significantly increased abundance of key enzymes in carbon degradation pathways. | | | | | | | |
| --- | --- | --- | --- | --- | --- | --- | --- |
| Function | Taxon | T2Ck_a | T2Ck_b | T2Ck_c | T2B300_a | T2B300_b | T2B300_c |
| 3.2.1.4 | p__Proteobacteria | 696 | 716 | 586 | 2940 | 3002 | 2680 |
| 3.2.1.4 | p__Actinobacteria | 676 | 612 | 714 | 632 | 808 | 562 |
| 3.2.1.4 | p__Bacteroidetes | 78 | 96 | 50 | 116 | 118 | 60 |
| 3.2.1.4 | p__Chloroflexi | 36 | 38 | 14 | 2 | 16 | 12 |
| 3.2.1.4 | p__Firmicutes | 4 | 12 | 0 | 98 | 128 | 74 |
| 3.2.1.4 | p__Acidobacteria | 0 | 0 | 0 | 0 | 0 | 0 |
| 3.2.1.4 | p__Gemmatimonadetes | 6 | 4 | 10 | 10 | 16 | 6 |
| 3.2.1.4 | p__Nitrospirae | 0 | 0 | 0 | 0 | 0 | 0 |
| 3.2.1.4 | p__unclassified_d__Bacteria | 10 | 42 | 28 | 64 | 66 | 40 |
| 3.2.1.4 | p__Cyanobacteria | 0 | 0 | 0 | 0 | 0 | 0 |
| 3.2.1.4 | p__Planctomycetes | 0 | 0 | 0 | 0 | 0 | 0 |
| 3.2.1.4 | p__Deinococcus-Thermus | 0 | 0 | 0 | 0 | 0 | 0 |
| 3.2.1.4 | p__Verrucomicrobia | 0 | 0 | 0 | 0 | 2 | 4 |
| 3.2.1.4 | p__Armatimonadetes | 0 | 0 | 0 | 0 | 0 | 0 |
| 3.2.1.4 | p__Candidatus_Tectomicrobia | 0 | 0 | 0 | 0 | 0 | 0 |
| 3.2.1.4 | p__Candidatus_Rokubacteria | 0 | 0 | 0 | 0 | 0 | 0 |
| 3.2.1.4 | p__candidate_division_NC10 | 0 | 0 | 0 | 0 | 0 | 0 |
| 3.2.1.4 | p__candidate_division_Zixibacteria | 0 | 0 | 0 | 0 | 0 | 0 |
| 3.2.1.4 | p__Spirochaetes | 0 | 0 | 0 | 0 | 0 | 0 |
| 3.2.1.4 | p__Candidatus_Acetothermia | 0 | 0 | 0 | 0 | 0 | 0 |
| 3.2.1.4 | p__Candidatus_Dadabacteria | 0 | 0 | 0 | 0 | 0 | 0 |
| 3.2.1.4 | p__Chlorobi | 0 | 0 | 0 | 0 | 0 | 0 |
| 3.2.1.4 | p__Candidatus_Omnitrophica | 0 | 0 | 0 | 0 | 0 | 0 |
| 3.2.1.4 | p__Aquificae | 0 | 0 | 0 | 0 | 0 | 0 |
| 3.2.1.4 | p__Thermodesulfobacteria | 0 | 0 | 0 | 0 | 0 | 0 |
| 3.2.1.4 | p__Latescibacteria | 0 | 0 | 0 | 0 | 0 | 0 |
| 3.2.1.4 | p__Candidatus_Gottesmanbacteria | 0 | 0 | 0 | 0 | 0 | 0 |
| 3.2.1.4 | p__Synergistetes | 0 | 0 | 0 | 0 | 0 | 0 |
| 3.2.1.4 | p__Candidatus_Woesebacteria | 0 | 0 | 0 | 0 | 0 | 0 |
| 3.2.1.4 | p__Thermotogae | 0 | 0 | 0 | 0 | 0 | 0 |
| 3.2.1.4 | others | 4 | 0 | 10 | 0 | 4 | 6 |
| 3.2.1.22 | p__Proteobacteria | 288 | 212 | 190 | 1532 | 1632 | 1604 |
| 3.2.1.22 | p__Actinobacteria | 674 | 684 | 704 | 596 | 700 | 594 |
| 3.2.1.22 | p__Bacteroidetes | 54 | 36 | 24 | 102 | 114 | 52 |
| 3.2.1.22 | p__Chloroflexi | 18 | 46 | 16 | 22 | 20 | 22 |
| 3.2.1.22 | p__Firmicutes | 24 | 24 | 14 | 132 | 170 | 74 |
| 3.2.1.22 | p__Acidobacteria | 2 | 10 | 20 | 0 | 2 | 0 |
| 3.2.1.22 | p__Gemmatimonadetes | 0 | 0 | 0 | 0 | 0 | 0 |
| 3.2.1.22 | p__Nitrospirae | 0 | 0 | 0 | 0 | 0 | 0 |
| 3.2.1.22 | p__unclassified_d__Bacteria | 0 | 8 | 0 | 18 | 24 | 16 |
| 3.2.1.22 | p__Cyanobacteria | 0 | 0 | 0 | 0 | 0 | 0 |
| 3.2.1.22 | p__Planctomycetes | 2 | 6 | 10 | 2 | 8 | 18 |
| 3.2.1.22 | p__Deinococcus-Thermus | 0 | 0 | 0 | 0 | 0 | 0 |
| 3.2.1.22 | p__Verrucomicrobia | 16 | 0 | 6 | 18 | 20 | 6 |
| 3.2.1.22 | p__Armatimonadetes | 0 | 0 | 0 | 0 | 0 | 0 |
| 3.2.1.22 | p__Candidatus_Tectomicrobia | 0 | 0 | 0 | 0 | 0 | 0 |
| 3.2.1.22 | p__Candidatus_Rokubacteria | 0 | 0 | 0 | 0 | 0 | 0 |
| 3.2.1.22 | p__candidate_division_NC10 | 0 | 0 | 0 | 0 | 0 | 0 |
| 3.2.1.22 | p__candidate_division_Zixibacteria | 0 | 0 | 0 | 0 | 0 | 0 |
| 3.2.1.22 | p__Spirochaetes | 0 | 0 | 0 | 0 | 0 | 0 |
| 3.2.1.22 | p__Candidatus_Acetothermia | 0 | 0 | 0 | 0 | 0 | 0 |
| 3.2.1.22 | p__Candidatus_Dadabacteria | 0 | 0 | 0 | 0 | 0 | 0 |
| 3.2.1.22 | p__Chlorobi | 0 | 0 | 0 | 0 | 0 | 0 |
| 3.2.1.22 | p__Candidatus_Omnitrophica | 0 | 0 | 0 | 0 | 0 | 0 |
| 3.2.1.22 | p__Aquificae | 0 | 0 | 0 | 0 | 0 | 0 |
| 3.2.1.22 | p__Thermodesulfobacteria | 0 | 0 | 0 | 0 | 0 | 0 |
| 3.2.1.22 | p__Latescibacteria | 0 | 0 | 0 | 0 | 0 | 0 |
| 3.2.1.22 | p__Candidatus_Gottesmanbacteria | 0 | 0 | 0 | 0 | 0 | 0 |
| 3.2.1.22 | p__Synergistetes | 0 | 0 | 0 | 0 | 0 | 0 |
| 3.2.1.22 | p__Candidatus_Woesebacteria | 0 | 0 | 0 | 0 | 0 | 0 |
| 3.2.1.22 | p__Thermotogae | 0 | 0 | 0 | 0 | 0 | 0 |
| 3.2.1.22 | others | 0 | 0 | 0 | 0 | 0 | 0 |
| 3.2.1.37 | p__Proteobacteria | 420 | 396 | 332 | 1338 | 1628 | 1482 |
| 3.2.1.37 | p__Actinobacteria | 118 | 82 | 120 | 144 | 130 | 110 |
| 3.2.1.37 | p__Bacteroidetes | 54 | 66 | 22 | 172 | 154 | 58 |
| 3.2.1.37 | p__Chloroflexi | 92 | 134 | 126 | 56 | 92 | 88 |
| 3.2.1.37 | p__Firmicutes | 10 | 2 | 0 | 106 | 84 | 58 |
| 3.2.1.37 | p__Acidobacteria | 6 | 18 | 2 | 4 | 8 | 2 |
| 3.2.1.37 | p__Gemmatimonadetes | 0 | 0 | 0 | 0 | 0 | 0 |
| 3.2.1.37 | p__Nitrospirae | 0 | 0 | 0 | 0 | 0 | 0 |
| 3.2.1.37 | p__unclassified_d__Bacteria | 0 | 0 | 0 | 0 | 0 | 0 |
| 3.2.1.37 | p__Cyanobacteria | 0 | 0 | 0 | 0 | 0 | 0 |
| 3.2.1.37 | p__Planctomycetes | 0 | 0 | 0 | 0 | 0 | 0 |
| 3.2.1.37 | p__Deinococcus-Thermus | 0 | 0 | 0 | 0 | 0 | 0 |
| 3.2.1.37 | p__Verrucomicrobia | 2 | 0 | 0 | 14 | 20 | 18 |
| 3.2.1.37 | p__Armatimonadetes | 0 | 0 | 0 | 0 | 0 | 0 |
| 3.2.1.37 | p__Candidatus_Tectomicrobia | 0 | 0 | 0 | 0 | 0 | 0 |
| 3.2.1.37 | p__Candidatus_Rokubacteria | 0 | 0 | 0 | 0 | 0 | 0 |
| 3.2.1.37 | p__candidate_division_NC10 | 0 | 0 | 0 | 0 | 0 | 0 |
| 3.2.1.37 | p__candidate_division_Zixibacteria | 0 | 0 | 0 | 0 | 0 | 0 |
| 3.2.1.37 | p__Spirochaetes | 0 | 0 | 0 | 0 | 0 | 0 |
| 3.2.1.37 | p__Candidatus_Acetothermia | 0 | 0 | 0 | 0 | 0 | 0 |
| 3.2.1.37 | p__Candidatus_Dadabacteria | 0 | 0 | 0 | 0 | 0 | 0 |
| 3.2.1.37 | p__Chlorobi | 0 | 0 | 0 | 0 | 0 | 0 |
| 3.2.1.37 | p__Candidatus_Omnitrophica | 0 | 0 | 0 | 0 | 0 | 0 |
| 3.2.1.37 | p__Aquificae | 0 | 0 | 0 | 0 | 0 | 0 |
| 3.2.1.37 | p__Thermodesulfobacteria | 0 | 0 | 0 | 0 | 0 | 0 |
| 3.2.1.37 | p__Latescibacteria | 0 | 0 | 0 | 0 | 0 | 0 |
| 3.2.1.37 | p__Candidatus_Gottesmanbacteria | 0 | 0 | 0 | 0 | 0 | 0 |
| 3.2.1.37 | p__Synergistetes | 0 | 0 | 0 | 0 | 0 | 0 |
| 3.2.1.37 | p__Candidatus_Woesebacteria | 0 | 0 | 0 | 0 | 0 | 0 |
| 3.2.1.37 | p__Thermotogae | 0 | 0 | 0 | 0 | 0 | 0 |
| 3.2.1.37 | others | 0 | 2 | 0 | 16 | 10 | 10 |
| 4.2.2.2 | p__Proteobacteria | 60 | 64 | 38 | 656 | 598 | 606 |
| 4.2.2.2 | p__Actinobacteria | 68 | 48 | 50 | 52 | 52 | 56 |
| 4.2.2.2 | p__Bacteroidetes | 12 | 30 | 6 | 118 | 48 | 34 |
| 4.2.2.2 | p__Chloroflexi | 0 | 0 | 0 | 0 | 0 | 0 |
| 4.2.2.2 | p__Firmicutes | 0 | 46 | 0 | 0 | 0 | 0 |
| 4.2.2.2 | p__Acidobacteria | 0 | 0 | 0 | 0 | 0 | 0 |
| 4.2.2.2 | p__Gemmatimonadetes | 0 | 0 | 0 | 0 | 0 | 0 |
| 4.2.2.2 | p__Nitrospirae | 0 | 0 | 0 | 0 | 0 | 0 |
| 4.2.2.2 | p__unclassified_d__Bacteria | 2 | 2 | 0 | 0 | 0 | 0 |
| 4.2.2.2 | p__Cyanobacteria | 0 | 0 | 0 | 0 | 0 | 0 |
| 4.2.2.2 | p__Planctomycetes | 0 | 0 | 0 | 0 | 0 | 0 |
| 4.2.2.2 | p__Deinococcus-Thermus | 0 | 0 | 0 | 0 | 0 | 0 |
| 4.2.2.2 | p__Verrucomicrobia | 0 | 0 | 0 | 0 | 0 | 0 |
| 4.2.2.2 | p__Armatimonadetes | 0 | 0 | 0 | 0 | 0 | 0 |
| 4.2.2.2 | p__Candidatus_Tectomicrobia | 0 | 0 | 0 | 0 | 0 | 0 |
| 4.2.2.2 | p__Candidatus_Rokubacteria | 0 | 0 | 0 | 0 | 0 | 0 |
| 4.2.2.2 | p__candidate_division_NC10 | 0 | 0 | 0 | 0 | 0 | 0 |
| 4.2.2.2 | p__candidate_division_Zixibacteria | 0 | 0 | 0 | 0 | 0 | 0 |
| 4.2.2.2 | p__Spirochaetes | 0 | 0 | 0 | 0 | 0 | 0 |
| 4.2.2.2 | p__Candidatus_Acetothermia | 0 | 0 | 0 | 0 | 0 | 0 |
| 4.2.2.2 | p__Candidatus_Dadabacteria | 0 | 0 | 0 | 0 | 0 | 0 |
| 4.2.2.2 | p__Chlorobi | 0 | 0 | 0 | 0 | 0 | 0 |
| 4.2.2.2 | p__Candidatus_Omnitrophica | 0 | 0 | 0 | 0 | 0 | 0 |
| 4.2.2.2 | p__Aquificae | 0 | 0 | 0 | 0 | 0 | 0 |
| 4.2.2.2 | p__Thermodesulfobacteria | 0 | 0 | 0 | 0 | 0 | 0 |
| 4.2.2.2 | p__Latescibacteria | 0 | 0 | 0 | 0 | 0 | 0 |
| 4.2.2.2 | p__Candidatus_Gottesmanbacteria | 0 | 0 | 0 | 0 | 0 | 0 |
| 4.2.2.2 | p__Synergistetes | 0 | 0 | 0 | 0 | 0 | 0 |
| 4.2.2.2 | p__Candidatus_Woesebacteria | 0 | 0 | 0 | 0 | 0 | 0 |
| 4.2.2.2 | p__Thermotogae | 0 | 0 | 0 | 0 | 0 | 0 |
| 4.2.2.2 | others | 0 | 0 | 0 | 0 | 0 | 0 |
| 3.2.1.139 | p__Proteobacteria | 132 | 176 | 102 | 452 | 414 | 426 |
| 3.2.1.139 | p__Actinobacteria | 30 | 22 | 18 | 24 | 22 | 18 |
| 3.2.1.139 | p__Bacteroidetes | 38 | 36 | 22 | 110 | 92 | 78 |
| 3.2.1.139 | p__Chloroflexi | 0 | 0 | 0 | 0 | 0 | 0 |
| 3.2.1.139 | p__Firmicutes | 0 | 0 | 0 | 8 | 30 | 22 |
| 3.2.1.139 | p__Acidobacteria | 8 | 0 | 2 | 2 | 18 | 4 |
| 3.2.1.139 | p__Gemmatimonadetes | 0 | 0 | 0 | 0 | 0 | 0 |
| 3.2.1.139 | p__Nitrospirae | 0 | 0 | 0 | 0 | 0 | 0 |
| 3.2.1.139 | p__unclassified_d__Bacteria | 0 | 0 | 0 | 0 | 0 | 0 |
| 3.2.1.139 | p__Cyanobacteria | 0 | 0 | 0 | 0 | 0 | 0 |
| 3.2.1.139 | p__Planctomycetes | 0 | 0 | 0 | 0 | 0 | 0 |
| 3.2.1.139 | p__Deinococcus-Thermus | 0 | 0 | 0 | 0 | 0 | 0 |
| 3.2.1.139 | p__Verrucomicrobia | 6 | 0 | 8 | 16 | 2 | 6 |
| 3.2.1.139 | p__Armatimonadetes | 0 | 0 | 0 | 0 | 0 | 0 |
| 3.2.1.139 | p__Candidatus_Tectomicrobia | 0 | 0 | 0 | 0 | 0 | 0 |
| 3.2.1.139 | p__Candidatus_Rokubacteria | 0 | 0 | 0 | 0 | 0 | 0 |
| 3.2.1.139 | p__candidate_division_NC10 | 0 | 0 | 0 | 0 | 0 | 0 |
| 3.2.1.139 | p__candidate_division_Zixibacteria | 0 | 0 | 0 | 0 | 0 | 0 |
| 3.2.1.139 | p__Spirochaetes | 0 | 0 | 0 | 0 | 0 | 0 |
| 3.2.1.139 | p__Candidatus_Acetothermia | 0 | 0 | 0 | 0 | 0 | 0 |
| 3.2.1.139 | p__Candidatus_Dadabacteria | 0 | 0 | 0 | 0 | 0 | 0 |
| 3.2.1.139 | p__Chlorobi | 0 | 0 | 0 | 0 | 0 | 0 |
| 3.2.1.139 | p__Candidatus_Omnitrophica | 0 | 0 | 0 | 0 | 0 | 0 |
| 3.2.1.139 | p__Aquificae | 0 | 0 | 0 | 0 | 0 | 0 |
| 3.2.1.139 | p__Thermodesulfobacteria | 0 | 0 | 0 | 0 | 0 | 0 |
| 3.2.1.139 | p__Latescibacteria | 0 | 0 | 0 | 0 | 0 | 0 |
| 3.2.1.139 | p__Candidatus_Gottesmanbacteria | 0 | 0 | 0 | 0 | 0 | 0 |
| 3.2.1.139 | p__Synergistetes | 0 | 0 | 0 | 0 | 0 | 0 |
| 3.2.1.139 | p__Candidatus_Woesebacteria | 0 | 0 | 0 | 0 | 0 | 0 |
| 3.2.1.139 | p__Thermotogae | 0 | 0 | 0 | 0 | 0 | 0 |
| 3.2.1.139 | others | 0 | 0 | 0 | 0 | 0 | 0 |
| 3.1.1.11 | p__Proteobacteria | 22 | 30 | 26 | 360 | 372 | 438 |
| 3.1.1.11 | p__Actinobacteria | 26 | 12 | 22 | 18 | 20 | 8 |
| 3.1.1.11 | p__Bacteroidetes | 58 | 56 | 64 | 198 | 154 | 90 |
| 3.1.1.11 | p__Chloroflexi | 0 | 0 | 0 | 0 | 0 | 0 |
| 3.1.1.11 | p__Firmicutes | 0 | 0 | 0 | 8 | 20 | 46 |
| 3.1.1.11 | p__Acidobacteria | 0 | 0 | 0 | 0 | 0 | 0 |
| 3.1.1.11 | p__Gemmatimonadetes | 0 | 0 | 0 | 0 | 0 | 0 |
| 3.1.1.11 | p__Nitrospirae | 0 | 0 | 0 | 0 | 0 | 0 |
| 3.1.1.11 | p__unclassified_d__Bacteria | 0 | 0 | 0 | 0 | 0 | 0 |
| 3.1.1.11 | p__Cyanobacteria | 0 | 0 | 0 | 0 | 0 | 0 |
| 3.1.1.11 | p__Planctomycetes | 0 | 0 | 0 | 0 | 0 | 0 |
| 3.1.1.11 | p__Deinococcus-Thermus | 0 | 0 | 0 | 0 | 0 | 0 |
| 3.1.1.11 | p__Verrucomicrobia | 0 | 0 | 0 | 0 | 0 | 0 |
| 3.1.1.11 | p__Armatimonadetes | 0 | 0 | 0 | 0 | 0 | 0 |
| 3.1.1.11 | p__Candidatus_Tectomicrobia | 0 | 0 | 0 | 0 | 0 | 0 |
| 3.1.1.11 | p__Candidatus_Rokubacteria | 0 | 0 | 0 | 0 | 0 | 0 |
| 3.1.1.11 | p__candidate_division_NC10 | 0 | 0 | 0 | 0 | 0 | 0 |
| 3.1.1.11 | p__candidate_division_Zixibacteria | 0 | 0 | 0 | 0 | 0 | 0 |
| 3.1.1.11 | p__Spirochaetes | 0 | 0 | 0 | 0 | 0 | 0 |
| 3.1.1.11 | p__Candidatus_Acetothermia | 0 | 0 | 0 | 0 | 0 | 0 |
| 3.1.1.11 | p__Candidatus_Dadabacteria | 0 | 0 | 0 | 0 | 0 | 0 |
| 3.1.1.11 | p__Chlorobi | 0 | 0 | 0 | 0 | 0 | 0 |
| 3.1.1.11 | p__Candidatus_Omnitrophica | 0 | 0 | 0 | 0 | 0 | 0 |
| 3.1.1.11 | p__Aquificae | 0 | 0 | 0 | 0 | 0 | 0 |
| 3.1.1.11 | p__Thermodesulfobacteria | 0 | 0 | 0 | 0 | 0 | 0 |
| 3.1.1.11 | p__Latescibacteria | 0 | 0 | 0 | 0 | 0 | 0 |
| 3.1.1.11 | p__Candidatus_Gottesmanbacteria | 0 | 0 | 0 | 0 | 0 | 0 |
| 3.1.1.11 | p__Synergistetes | 0 | 0 | 0 | 0 | 0 | 0 |
| 3.1.1.11 | p__Candidatus_Woesebacteria | 0 | 0 | 0 | 0 | 0 | 0 |
| 3.1.1.11 | p__Thermotogae | 0 | 0 | 0 | 0 | 0 | 0 |
| 3.1.1.11 | others | 0 | 0 | 0 | 0 | 0 | 0 |

| Table S8. Distribution of bacteria communities at order level contributed to the significantly increased abundance of key enzymes in carbon degradation pathways. | | | | | | | |
| --- | --- | --- | --- | --- | --- | --- | --- |
| Function | Taxon | T2Ck_a | T2Ck_b | T2Ck_c | T2B300_a | T2B300_b | T2B300_c |
| 3.2.1.4 | o__Burkholderiales | 166 | 114 | 54 | 310 | 354 | 352 |
| 3.2.1.4 | o__Pseudomonadales | 2 | 0 | 0 | 0 | 2 | 4 |
| 3.2.1.4 | o__Xanthomonadales | 78 | 72 | 80 | 238 | 300 | 254 |
| 3.2.1.4 | o__Solirubrobacterales | 50 | 32 | 54 | 40 | 26 | 20 |
| 3.2.1.4 | o__Rhizobiales | 96 | 80 | 96 | 176 | 288 | 216 |
| 3.2.1.4 | o__Enterobacteriales | 2 | 2 | 0 | 676 | 710 | 728 |
| 3.2.1.4 | o__Propionibacteriales | 42 | 26 | 28 | 24 | 64 | 30 |
| 3.2.1.4 | o__Sphingomonadales | 56 | 70 | 50 | 60 | 90 | 68 |
| 3.2.1.4 | o__Micrococcales | 32 | 36 | 28 | 58 | 68 | 44 |
| 3.2.1.4 | o__Streptomycetales | 244 | 220 | 280 | 218 | 250 | 192 |
| 3.2.1.4 | o__Rhodospirillales | 18 | 12 | 8 | 308 | 388 | 382 |
| 3.2.1.4 | o__Rubrobacterales | 0 | 0 | 0 | 0 | 0 | 0 |
| 3.2.1.4 | o__Flavobacteriales | 58 | 64 | 34 | 40 | 46 | 26 |
| 3.2.1.4 | o__Neisseriales | 0 | 0 | 0 | 0 | 0 | 0 |
| 3.2.1.4 | o__Cellvibrionales | 190 | 222 | 174 | 1064 | 754 | 568 |
| 3.2.1.4 | o__Pseudonocardiales | 12 | 22 | 2 | 16 | 12 | 24 |
| 3.2.1.4 | o__Corynebacteriales | 240 | 220 | 258 | 196 | 288 | 176 |
| 3.2.1.4 | o__Streptosporangiales | 12 | 12 | 26 | 8 | 4 | 4 |
| 3.2.1.4 | o__Gemmatimonadales | 6 | 4 | 10 | 10 | 16 | 6 |
| 3.2.1.4 | o__unclassified_p__Chloroflexi | 0 | 0 | 0 | 0 | 0 | 0 |
| 3.2.1.4 | o__Nitrospirales | 0 | 0 | 0 | 0 | 0 | 0 |
| 3.2.1.4 | o__Micromonosporales | 16 | 12 | 28 | 44 | 30 | 36 |
| 3.2.1.4 | o__Myxococcales | 54 | 100 | 80 | 38 | 58 | 48 |
| 3.2.1.4 | o__Gaiellales | 0 | 0 | 0 | 0 | 0 | 0 |
| 3.2.1.4 | o__unclassified_d__Bacteria | 10 | 42 | 28 | 64 | 66 | 40 |
| 3.2.1.4 | o__Clostridiales | 4 | 12 | 0 | 98 | 112 | 68 |
| 3.2.1.4 | o__unclassified_c__Actinobacteria | 12 | 18 | 2 | 2 | 8 | 10 |
| 3.2.1.4 | o__Aeromonadales | 0 | 0 | 0 | 0 | 0 | 0 |
| 3.2.1.4 | o__Geodermatophilales | 4 | 6 | 6 | 10 | 24 | 20 |
| 3.2.1.4 | o__unclassified_p__Acidobacteria | 0 | 0 | 0 | 0 | 0 | 0 |
| 3.2.1.4 | others | 106 | 122 | 86 | 164 | 202 | 128 |
| 3.2.1.22 | o__Burkholderiales | 20 | 8 | 22 | 56 | 42 | 32 |
| 3.2.1.22 | o__Pseudomonadales | 0 | 0 | 0 | 0 | 0 | 0 |
| 3.2.1.22 | o__Xanthomonadales | 62 | 38 | 56 | 242 | 250 | 192 |
| 3.2.1.22 | o__Solirubrobacterales | 58 | 54 | 64 | 54 | 76 | 46 |
| 3.2.1.22 | o__Rhizobiales | 158 | 138 | 84 | 464 | 512 | 390 |
| 3.2.1.22 | o__Enterobacteriales | 2 | 0 | 0 | 624 | 602 | 706 |
| 3.2.1.22 | o__Propionibacteriales | 88 | 66 | 84 | 52 | 136 | 92 |
| 3.2.1.22 | o__Sphingomonadales | 14 | 8 | 6 | 44 | 48 | 58 |
| 3.2.1.22 | o__Micrococcales | 82 | 62 | 80 | 96 | 88 | 98 |
| 3.2.1.22 | o__Streptomycetales | 24 | 88 | 42 | 26 | 36 | 44 |
| 3.2.1.22 | o__Rhodospirillales | 6 | 2 | 0 | 40 | 92 | 120 |
| 3.2.1.22 | o__Rubrobacterales | 76 | 110 | 56 | 48 | 26 | 22 |
| 3.2.1.22 | o__Flavobacteriales | 42 | 22 | 4 | 34 | 40 | 8 |
| 3.2.1.22 | o__Neisseriales | 0 | 0 | 0 | 0 | 0 | 0 |
| 3.2.1.22 | o__Cellvibrionales | 16 | 4 | 6 | 16 | 14 | 22 |
| 3.2.1.22 | o__Pseudonocardiales | 8 | 2 | 4 | 2 | 2 | 12 |
| 3.2.1.22 | o__Corynebacteriales | 6 | 10 | 8 | 6 | 6 | 0 |
| 3.2.1.22 | o__Streptosporangiales | 2 | 8 | 0 | 0 | 8 | 0 |
| 3.2.1.22 | o__Gemmatimonadales | 0 | 0 | 0 | 0 | 0 | 0 |
| 3.2.1.22 | o__unclassified_p__Chloroflexi | 4 | 32 | 4 | 12 | 12 | 6 |
| 3.2.1.22 | o__Nitrospirales | 0 | 0 | 0 | 0 | 0 | 0 |
| 3.2.1.22 | o__Micromonosporales | 232 | 206 | 272 | 220 | 226 | 208 |
| 3.2.1.22 | o__Myxococcales | 0 | 0 | 0 | 0 | 0 | 0 |
| 3.2.1.22 | o__Gaiellales | 0 | 0 | 0 | 0 | 0 | 0 |
| 3.2.1.22 | o__unclassified_d__Bacteria | 0 | 8 | 0 | 18 | 24 | 16 |
| 3.2.1.22 | o__Clostridiales | 10 | 8 | 0 | 90 | 130 | 52 |
| 3.2.1.22 | o__unclassified_c__Actinobacteria | 24 | 24 | 36 | 38 | 44 | 32 |
| 3.2.1.22 | o__Aeromonadales | 0 | 0 | 0 | 34 | 52 | 50 |
| 3.2.1.22 | o__Geodermatophilales | 44 | 36 | 38 | 32 | 38 | 22 |
| 3.2.1.22 | o__unclassified_p__Acidobacteria | 0 | 0 | 0 | 0 | 0 | 0 |
| 3.2.1.22 | others | 100 | 92 | 118 | 174 | 186 | 158 |
| 3.2.1.37 | o__Burkholderiales | 26 | 4 | 22 | 72 | 44 | 72 |
| 3.2.1.37 | o__Pseudomonadales | 0 | 0 | 0 | 0 | 0 | 0 |
| 3.2.1.37 | o__Xanthomonadales | 148 | 176 | 132 | 480 | 578 | 546 |
| 3.2.1.37 | o__Solirubrobacterales | 18 | 8 | 18 | 2 | 2 | 8 |
| 3.2.1.37 | o__Rhizobiales | 86 | 70 | 46 | 266 | 314 | 240 |
| 3.2.1.37 | o__Enterobacteriales | 0 | 0 | 0 | 316 | 412 | 374 |
| 3.2.1.37 | o__Propionibacteriales | 12 | 14 | 20 | 32 | 16 | 16 |
| 3.2.1.37 | o__Sphingomonadales | 106 | 108 | 98 | 172 | 238 | 184 |
| 3.2.1.37 | o__Micrococcales | 16 | 0 | 8 | 42 | 20 | 20 |
| 3.2.1.37 | o__Streptomycetales | 8 | 6 | 10 | 14 | 20 | 10 |
| 3.2.1.37 | o__Rhodospirillales | 8 | 6 | 10 | 4 | 8 | 0 |
| 3.2.1.37 | o__Rubrobacterales | 0 | 0 | 0 | 0 | 0 | 0 |
| 3.2.1.37 | o__Flavobacteriales | 34 | 30 | 14 | 110 | 98 | 32 |
| 3.2.1.37 | o__Neisseriales | 0 | 0 | 0 | 0 | 0 | 0 |
| 3.2.1.37 | o__Cellvibrionales | 0 | 0 | 0 | 0 | 0 | 0 |
| 3.2.1.37 | o__Pseudonocardiales | 0 | 0 | 0 | 0 | 0 | 0 |
| 3.2.1.37 | o__Corynebacteriales | 0 | 0 | 0 | 0 | 0 | 2 |
| 3.2.1.37 | o__Streptosporangiales | 0 | 0 | 0 | 0 | 0 | 0 |
| 3.2.1.37 | o__Gemmatimonadales | 0 | 0 | 0 | 0 | 0 | 0 |
| 3.2.1.37 | o__unclassified_p__Chloroflexi | 92 | 134 | 126 | 56 | 92 | 88 |
| 3.2.1.37 | o__Nitrospirales | 0 | 0 | 0 | 0 | 0 | 0 |
| 3.2.1.37 | o__Micromonosporales | 4 | 2 | 10 | 6 | 14 | 22 |
| 3.2.1.37 | o__Myxococcales | 0 | 0 | 0 | 0 | 0 | 0 |
| 3.2.1.37 | o__Gaiellales | 0 | 0 | 0 | 0 | 0 | 0 |
| 3.2.1.37 | o__unclassified_d__Bacteria | 0 | 0 | 0 | 0 | 0 | 0 |
| 3.2.1.37 | o__Clostridiales | 10 | 2 | 0 | 104 | 84 | 58 |
| 3.2.1.37 | o__unclassified_c__Actinobacteria | 0 | 0 | 0 | 0 | 0 | 0 |
| 3.2.1.37 | o__Aeromonadales | 0 | 0 | 0 | 0 | 0 | 0 |
| 3.2.1.37 | o__Geodermatophilales | 0 | 0 | 0 | 0 | 0 | 0 |
| 3.2.1.37 | o__unclassified_p__Acidobacteria | 6 | 18 | 2 | 4 | 8 | 2 |
| 3.2.1.37 | others | 128 | 122 | 86 | 170 | 178 | 152 |
| 4.2.2.2 | o__Burkholderiales | 18 | 8 | 2 | 52 | 44 | 50 |
| 4.2.2.2 | o__Pseudomonadales | 0 | 0 | 0 | 0 | 0 | 0 |
| 4.2.2.2 | o__Xanthomonadales | 2 | 12 | 2 | 8 | 2 | 2 |
| 4.2.2.2 | o__Solirubrobacterales | 0 | 0 | 0 | 0 | 0 | 0 |
| 4.2.2.2 | o__Rhizobiales | 0 | 0 | 0 | 14 | 20 | 12 |
| 4.2.2.2 | o__Enterobacteriales | 2 | 0 | 0 | 412 | 400 | 416 |
| 4.2.2.2 | o__Propionibacteriales | 0 | 0 | 0 | 0 | 0 | 0 |
| 4.2.2.2 | o__Sphingomonadales | 0 | 0 | 0 | 0 | 0 | 0 |
| 4.2.2.2 | o__Micrococcales | 2 | 2 | 2 | 20 | 10 | 20 |
| 4.2.2.2 | o__Streptomycetales | 62 | 44 | 48 | 30 | 36 | 34 |
| 4.2.2.2 | o__Rhodospirillales | 0 | 0 | 0 | 0 | 0 | 0 |
| 4.2.2.2 | o__Rubrobacterales | 0 | 0 | 0 | 0 | 0 | 0 |
| 4.2.2.2 | o__Flavobacteriales | 12 | 30 | 6 | 118 | 48 | 34 |
| 4.2.2.2 | o__Neisseriales | 0 | 0 | 0 | 0 | 0 | 0 |
| 4.2.2.2 | o__Cellvibrionales | 34 | 22 | 26 | 72 | 84 | 58 |
| 4.2.2.2 | o__Pseudonocardiales | 0 | 0 | 0 | 0 | 0 | 0 |
| 4.2.2.2 | o__Corynebacteriales | 0 | 0 | 0 | 0 | 0 | 0 |
| 4.2.2.2 | o__Streptosporangiales | 0 | 0 | 0 | 0 | 0 | 0 |
| 4.2.2.2 | o__Gemmatimonadales | 0 | 0 | 0 | 0 | 0 | 0 |
| 4.2.2.2 | o__unclassified_p__Chloroflexi | 0 | 0 | 0 | 0 | 0 | 0 |
| 4.2.2.2 | o__Nitrospirales | 0 | 0 | 0 | 0 | 0 | 0 |
| 4.2.2.2 | o__Micromonosporales | 4 | 2 | 0 | 2 | 6 | 2 |
| 4.2.2.2 | o__Myxococcales | 0 | 0 | 0 | 0 | 0 | 0 |
| 4.2.2.2 | o__Gaiellales | 0 | 0 | 0 | 0 | 0 | 0 |
| 4.2.2.2 | o__unclassified_d__Bacteria | 2 | 2 | 0 | 0 | 0 | 0 |
| 4.2.2.2 | o__Clostridiales | 0 | 0 | 0 | 0 | 0 | 0 |
| 4.2.2.2 | o__unclassified_c__Actinobacteria | 0 | 0 | 0 | 0 | 0 | 0 |
| 4.2.2.2 | o__Aeromonadales | 0 | 0 | 0 | 0 | 0 | 0 |
| 4.2.2.2 | o__Geodermatophilales | 0 | 0 | 0 | 0 | 0 | 0 |
| 4.2.2.2 | o__unclassified_p__Acidobacteria | 0 | 0 | 0 | 0 | 0 | 0 |
| 4.2.2.2 | others | 4 | 68 | 8 | 98 | 48 | 68 |
| 3.2.1.139 | o__Burkholderiales | 0 | 0 | 0 | 0 | 0 | 0 |
| 3.2.1.139 | o__Pseudomonadales | 0 | 0 | 0 | 0 | 0 | 0 |
| 3.2.1.139 | o__Xanthomonadales | 44 | 82 | 48 | 222 | 202 | 244 |
| 3.2.1.139 | o__Solirubrobacterales | 0 | 0 | 0 | 0 | 0 | 0 |
| 3.2.1.139 | o__Rhizobiales | 0 | 0 | 0 | 0 | 0 | 0 |
| 3.2.1.139 | o__Enterobacteriales | 0 | 0 | 0 | 0 | 0 | 0 |
| 3.2.1.139 | o__Propionibacteriales | 0 | 0 | 0 | 0 | 0 | 0 |
| 3.2.1.139 | o__Sphingomonadales | 54 | 58 | 42 | 84 | 106 | 76 |
| 3.2.1.139 | o__Micrococcales | 22 | 14 | 16 | 16 | 18 | 12 |
| 3.2.1.139 | o__Streptomycetales | 6 | 8 | 2 | 8 | 4 | 4 |
| 3.2.1.139 | o__Rhodospirillales | 0 | 0 | 0 | 0 | 0 | 0 |
| 3.2.1.139 | o__Rubrobacterales | 0 | 0 | 0 | 0 | 0 | 0 |
| 3.2.1.139 | o__Flavobacteriales | 32 | 22 | 2 | 74 | 26 | 50 |
| 3.2.1.139 | o__Neisseriales | 0 | 0 | 0 | 0 | 0 | 0 |
| 3.2.1.139 | o__Cellvibrionales | 24 | 28 | 12 | 130 | 98 | 86 |
| 3.2.1.139 | o__Pseudonocardiales | 0 | 0 | 0 | 0 | 0 | 0 |
| 3.2.1.139 | o__Corynebacteriales | 0 | 0 | 0 | 0 | 0 | 0 |
| 3.2.1.139 | o__Streptosporangiales | 0 | 0 | 0 | 0 | 0 | 0 |
| 3.2.1.139 | o__Gemmatimonadales | 0 | 0 | 0 | 0 | 0 | 0 |
| 3.2.1.139 | o__unclassified_p__Chloroflexi | 0 | 0 | 0 | 0 | 0 | 0 |
| 3.2.1.139 | o__Nitrospirales | 0 | 0 | 0 | 0 | 0 | 0 |
| 3.2.1.139 | o__Micromonosporales | 2 | 0 | 0 | 0 | 0 | 2 |
| 3.2.1.139 | o__Myxococcales | 0 | 0 | 0 | 0 | 0 | 0 |
| 3.2.1.139 | o__Gaiellales | 0 | 0 | 0 | 0 | 0 | 0 |
| 3.2.1.139 | o__unclassified_d__Bacteria | 0 | 0 | 0 | 0 | 0 | 0 |
| 3.2.1.139 | o__Clostridiales | 0 | 0 | 0 | 6 | 14 | 8 |
| 3.2.1.139 | o__unclassified_c__Actinobacteria | 0 | 0 | 0 | 0 | 0 | 0 |
| 3.2.1.139 | o__Aeromonadales | 0 | 0 | 0 | 0 | 0 | 0 |
| 3.2.1.139 | o__Geodermatophilales | 0 | 0 | 0 | 0 | 0 | 0 |
| 3.2.1.139 | o__unclassified_p__Acidobacteria | 4 | 0 | 2 | 0 | 10 | 0 |
| 3.2.1.139 | others | 26 | 22 | 28 | 72 | 100 | 72 |
| 3.1.1.11 | o__Burkholderiales | 0 | 0 | 0 | 0 | 0 | 0 |
| 3.1.1.11 | o__Pseudomonadales | 0 | 0 | 0 | 0 | 0 | 0 |
| 3.1.1.11 | o__Xanthomonadales | 4 | 2 | 0 | 2 | 6 | 6 |
| 3.1.1.11 | o__Solirubrobacterales | 0 | 0 | 0 | 0 | 0 | 0 |
| 3.1.1.11 | o__Rhizobiales | 0 | 0 | 0 | 0 | 0 | 0 |
| 3.1.1.11 | o__Enterobacteriales | 0 | 2 | 0 | 300 | 318 | 386 |
| 3.1.1.11 | o__Propionibacteriales | 0 | 0 | 0 | 0 | 0 | 0 |
| 3.1.1.11 | o__Sphingomonadales | 2 | 0 | 0 | 2 | 2 | 0 |
| 3.1.1.11 | o__Micrococcales | 2 | 0 | 6 | 16 | 8 | 2 |
| 3.1.1.11 | o__Streptomycetales | 24 | 12 | 16 | 2 | 12 | 6 |
| 3.1.1.11 | o__Rhodospirillales | 0 | 0 | 0 | 0 | 0 | 0 |
| 3.1.1.11 | o__Rubrobacterales | 0 | 0 | 0 | 0 | 0 | 0 |
| 3.1.1.11 | o__Flavobacteriales | 56 | 56 | 60 | 156 | 108 | 72 |
| 3.1.1.11 | o__Neisseriales | 0 | 0 | 0 | 0 | 0 | 0 |
| 3.1.1.11 | o__Cellvibrionales | 16 | 14 | 4 | 44 | 38 | 26 |
| 3.1.1.11 | o__Pseudonocardiales | 0 | 0 | 0 | 0 | 0 | 0 |
| 3.1.1.11 | o__Corynebacteriales | 0 | 0 | 0 | 0 | 0 | 0 |
| 3.1.1.11 | o__Streptosporangiales | 0 | 0 | 0 | 0 | 0 | 0 |
| 3.1.1.11 | o__Gemmatimonadales | 0 | 0 | 0 | 0 | 0 | 0 |
| 3.1.1.11 | o__unclassified_p__Chloroflexi | 0 | 0 | 0 | 0 | 0 | 0 |
| 3.1.1.11 | o__Nitrospirales | 0 | 0 | 0 | 0 | 0 | 0 |
| 3.1.1.11 | o__Micromonosporales | 0 | 0 | 0 | 0 | 0 | 0 |
| 3.1.1.11 | o__Myxococcales | 0 | 0 | 0 | 0 | 0 | 0 |
| 3.1.1.11 | o__Gaiellales | 0 | 0 | 0 | 0 | 0 | 0 |
| 3.1.1.11 | o__unclassified_d__Bacteria | 0 | 0 | 0 | 0 | 0 | 0 |
| 3.1.1.11 | o__Clostridiales | 0 | 0 | 0 | 8 | 20 | 46 |
| 3.1.1.11 | o__unclassified_c__Actinobacteria | 0 | 0 | 0 | 0 | 0 | 0 |
| 3.1.1.11 | o__Aeromonadales | 0 | 0 | 0 | 0 | 0 | 0 |
| 3.1.1.11 | o__Geodermatophilales | 0 | 0 | 0 | 0 | 0 | 0 |
| 3.1.1.11 | o__unclassified_p__Acidobacteria | 0 | 0 | 0 | 0 | 0 | 0 |
| 3.1.1.11 | others | 2 | 12 | 26 | 54 | 54 | 38 |

| Table S9. Distribution of bacteria communities at genera level contributed to the significantly increased abundance of key enzymes in carbon degradation pathways. | | | | | | | |
| --- | --- | --- | --- | --- | --- | --- | --- |
| Function | Taxon | T2Ck_a | T2Ck_b | T2Ck_c | T2B300_a | T2B300_b | T2B300_c |
| 3.2.1.4 | g__Pseudomonas | 0 | 0 | 0 | 0 | 0 | 0 |
| 3.2.1.4 | g__Lelliottia | 2 | 2 | 0 | 610 | 682 | 658 |
| 3.2.1.4 | g__Nocardioides | 42 | 26 | 28 | 24 | 64 | 30 |
| 3.2.1.4 | g__Pseudoxanthomonas | 52 | 52 | 42 | 190 | 216 | 206 |
| 3.2.1.4 | g__Achromobacter | 0 | 0 | 0 | 0 | 0 | 0 |
| 3.2.1.4 | g__Solirubrobacter | 0 | 0 | 0 | 0 | 0 | 0 |
| 3.2.1.4 | g__Delftia | 0 | 0 | 0 | 0 | 0 | 0 |
| 3.2.1.4 | g__Acidovorax | 138 | 90 | 24 | 272 | 266 | 288 |
| 3.2.1.4 | g__Streptomyces | 244 | 220 | 280 | 218 | 250 | 192 |
| 3.2.1.4 | g__Conexibacter | 50 | 32 | 54 | 40 | 26 | 20 |
| 3.2.1.4 | g__Azospirillum | 8 | 0 | 6 | 298 | 386 | 370 |
| 3.2.1.4 | g__Rubrobacter | 0 | 0 | 0 | 0 | 0 | 0 |
| 3.2.1.4 | g__Lysobacter | 14 | 18 | 22 | 36 | 56 | 34 |
| 3.2.1.4 | g__Flavobacterium | 58 | 64 | 32 | 40 | 46 | 26 |
| 3.2.1.4 | g__Rhizobium | 48 | 46 | 64 | 66 | 70 | 54 |
| 3.2.1.4 | g__Agrobacterium | 8 | 2 | 0 | 48 | 98 | 70 |
| 3.2.1.4 | g__Vogesella | 0 | 0 | 0 | 0 | 0 | 0 |
| 3.2.1.4 | g__Sphingomonas | 26 | 22 | 32 | 22 | 38 | 18 |
| 3.2.1.4 | g__Cellvibrio | 188 | 200 | 164 | 966 | 704 | 524 |
| 3.2.1.4 | g__unclassified_o__Solirubrobacterales | 0 | 0 | 0 | 0 | 0 | 0 |
| 3.2.1.4 | g__Arthrobacter | 10 | 0 | 4 | 6 | 2 | 0 |
| 3.2.1.4 | g__Sphingobium | 0 | 0 | 0 | 0 | 0 | 0 |
| 3.2.1.4 | g__Sphingopyxis | 28 | 48 | 16 | 38 | 52 | 50 |
| 3.2.1.4 | g__Variovorax | 0 | 0 | 0 | 0 | 0 | 0 |
| 3.2.1.4 | g__Nitrospira | 0 | 0 | 0 | 0 | 0 | 0 |
| 3.2.1.4 | g__unclassified_p__Chloroflexi | 0 | 0 | 0 | 0 | 0 | 0 |
| 3.2.1.4 | g__Gaiella | 0 | 0 | 0 | 0 | 0 | 0 |
| 3.2.1.4 | g__Enterobacter | 0 | 0 | 0 | 2 | 10 | 52 |
| 3.2.1.4 | g__Phyllobacterium | 6 | 0 | 2 | 12 | 16 | 18 |
| 3.2.1.4 | g__Acinetobacter | 0 | 0 | 0 | 0 | 0 | 0 |
| 3.2.1.4 | others | 588 | 698 | 642 | 974 | 1178 | 834 |
| 3.2.1.22 | g__Pseudomonas | 0 | 0 | 0 | 0 | 0 | 0 |
| 3.2.1.22 | g__Lelliottia | 0 | 0 | 0 | 490 | 522 | 560 |
| 3.2.1.22 | g__Nocardioides | 68 | 40 | 62 | 36 | 112 | 60 |
| 3.2.1.22 | g__Pseudoxanthomonas | 2 | 0 | 0 | 6 | 0 | 0 |
| 3.2.1.22 | g__Achromobacter | 0 | 0 | 0 | 0 | 0 | 0 |
| 3.2.1.22 | g__Solirubrobacter | 46 | 34 | 56 | 46 | 68 | 34 |
| 3.2.1.22 | g__Delftia | 0 | 0 | 0 | 0 | 0 | 0 |
| 3.2.1.22 | g__Acidovorax | 0 | 0 | 0 | 0 | 0 | 0 |
| 3.2.1.22 | g__Streptomyces | 24 | 88 | 42 | 26 | 36 | 44 |
| 3.2.1.22 | g__Conexibacter | 12 | 20 | 8 | 8 | 8 | 12 |
| 3.2.1.22 | g__Azospirillum | 6 | 2 | 0 | 40 | 92 | 120 |
| 3.2.1.22 | g__Rubrobacter | 76 | 110 | 56 | 48 | 26 | 22 |
| 3.2.1.22 | g__Lysobacter | 4 | 4 | 18 | 12 | 10 | 4 |
| 3.2.1.22 | g__Flavobacterium | 42 | 22 | 4 | 30 | 24 | 6 |
| 3.2.1.22 | g__Rhizobium | 38 | 26 | 38 | 114 | 126 | 74 |
| 3.2.1.22 | g__Agrobacterium | 40 | 20 | 4 | 168 | 212 | 158 |
| 3.2.1.22 | g__Vogesella | 0 | 0 | 0 | 0 | 0 | 0 |
| 3.2.1.22 | g__Sphingomonas | 14 | 6 | 6 | 10 | 20 | 30 |
| 3.2.1.22 | g__Cellvibrio | 16 | 4 | 6 | 16 | 14 | 22 |
| 3.2.1.22 | g__unclassified_o__Solirubrobacterales | 0 | 0 | 0 | 0 | 0 | 0 |
| 3.2.1.22 | g__Arthrobacter | 24 | 16 | 8 | 6 | 34 | 20 |
| 3.2.1.22 | g__Sphingobium | 0 | 0 | 0 | 0 | 0 | 0 |
| 3.2.1.22 | g__Sphingopyxis | 0 | 2 | 0 | 6 | 2 | 4 |
| 3.2.1.22 | g__Variovorax | 0 | 0 | 0 | 0 | 0 | 0 |
| 3.2.1.22 | g__Nitrospira | 0 | 0 | 0 | 0 | 0 | 0 |
| 3.2.1.22 | g__unclassified_p__Chloroflexi | 0 | 0 | 0 | 0 | 0 | 0 |
| 3.2.1.22 | g__Gaiella | 0 | 0 | 0 | 0 | 0 | 0 |
| 3.2.1.22 | g__Enterobacter | 0 | 0 | 0 | 16 | 22 | 112 |
| 3.2.1.22 | g__Phyllobacterium | 0 | 0 | 0 | 0 | 0 | 0 |
| 3.2.1.22 | g__Acinetobacter | 0 | 0 | 0 | 0 | 0 | 0 |
| 3.2.1.22 | others | 666 | 632 | 676 | 1344 | 1362 | 1104 |
| 3.2.1.37 | g__Pseudomonas | 0 | 0 | 0 | 0 | 0 | 0 |
| 3.2.1.37 | g__Lelliottia | 0 | 0 | 0 | 286 | 378 | 320 |
| 3.2.1.37 | g__Nocardioides | 12 | 10 | 10 | 26 | 8 | 16 |
| 3.2.1.37 | g__Pseudoxanthomonas | 140 | 170 | 122 | 456 | 552 | 528 |
| 3.2.1.37 | g__Achromobacter | 0 | 0 | 0 | 0 | 0 | 0 |
| 3.2.1.37 | g__Solirubrobacter | 18 | 8 | 18 | 2 | 2 | 8 |
| 3.2.1.37 | g__Delftia | 0 | 0 | 0 | 0 | 0 | 0 |
| 3.2.1.37 | g__Acidovorax | 0 | 0 | 0 | 0 | 0 | 0 |
| 3.2.1.37 | g__Streptomyces | 8 | 6 | 10 | 14 | 20 | 10 |
| 3.2.1.37 | g__Conexibacter | 0 | 0 | 0 | 0 | 0 | 0 |
| 3.2.1.37 | g__Azospirillum | 0 | 0 | 0 | 0 | 0 | 0 |
| 3.2.1.37 | g__Rubrobacter | 0 | 0 | 0 | 0 | 0 | 0 |
| 3.2.1.37 | g__Lysobacter | 4 | 2 | 2 | 2 | 8 | 2 |
| 3.2.1.37 | g__Flavobacterium | 34 | 26 | 14 | 110 | 92 | 32 |
| 3.2.1.37 | g__Rhizobium | 2 | 10 | 6 | 86 | 82 | 82 |
| 3.2.1.37 | g__Agrobacterium | 0 | 0 | 0 | 0 | 0 | 0 |
| 3.2.1.37 | g__Vogesella | 0 | 0 | 0 | 0 | 0 | 0 |
| 3.2.1.37 | g__Sphingomonas | 36 | 20 | 22 | 26 | 32 | 24 |
| 3.2.1.37 | g__Cellvibrio | 0 | 0 | 0 | 0 | 0 | 0 |
| 3.2.1.37 | g__unclassified_o__Solirubrobacterales | 0 | 0 | 0 | 0 | 0 | 0 |
| 3.2.1.37 | g__Arthrobacter | 0 | 0 | 0 | 0 | 0 | 0 |
| 3.2.1.37 | g__Sphingobium | 70 | 88 | 76 | 138 | 182 | 138 |
| 3.2.1.37 | g__Sphingopyxis | 0 | 0 | 0 | 2 | 2 | 0 |
| 3.2.1.37 | g__Variovorax | 0 | 0 | 0 | 0 | 0 | 0 |
| 3.2.1.37 | g__Nitrospira | 0 | 0 | 0 | 0 | 0 | 0 |
| 3.2.1.37 | g__unclassified_p__Chloroflexi | 0 | 0 | 0 | 0 | 0 | 0 |
| 3.2.1.37 | g__Gaiella | 0 | 0 | 0 | 0 | 0 | 0 |
| 3.2.1.37 | g__Enterobacter | 0 | 0 | 0 | 6 | 20 | 36 |
| 3.2.1.37 | g__Phyllobacterium | 0 | 0 | 0 | 0 | 0 | 0 |
| 3.2.1.37 | g__Acinetobacter | 0 | 0 | 0 | 0 | 0 | 0 |
| 3.2.1.37 | others | 378 | 360 | 322 | 696 | 748 | 630 |
| 4.2.2.2 | g__Pseudomonas | 0 | 0 | 0 | 0 | 0 | 0 |
| 4.2.2.2 | g__Lelliottia | 2 | 0 | 0 | 412 | 400 | 416 |
| 4.2.2.2 | g__Nocardioides | 0 | 0 | 0 | 0 | 0 | 0 |
| 4.2.2.2 | g__Pseudoxanthomonas | 0 | 0 | 0 | 0 | 0 | 0 |
| 4.2.2.2 | g__Achromobacter | 4 | 6 | 0 | 10 | 26 | 14 |
| 4.2.2.2 | g__Solirubrobacter | 0 | 0 | 0 | 0 | 0 | 0 |
| 4.2.2.2 | g__Delftia | 0 | 0 | 0 | 0 | 0 | 0 |
| 4.2.2.2 | g__Acidovorax | 0 | 0 | 0 | 0 | 0 | 0 |
| 4.2.2.2 | g__Streptomyces | 62 | 44 | 48 | 30 | 36 | 34 |
| 4.2.2.2 | g__Conexibacter | 0 | 0 | 0 | 0 | 0 | 0 |
| 4.2.2.2 | g__Azospirillum | 0 | 0 | 0 | 0 | 0 | 0 |
| 4.2.2.2 | g__Rubrobacter | 0 | 0 | 0 | 0 | 0 | 0 |
| 4.2.2.2 | g__Lysobacter | 2 | 12 | 2 | 8 | 2 | 2 |
| 4.2.2.2 | g__Flavobacterium | 12 | 30 | 6 | 118 | 48 | 34 |
| 4.2.2.2 | g__Rhizobium | 0 | 0 | 0 | 14 | 20 | 12 |
| 4.2.2.2 | g__Agrobacterium | 0 | 0 | 0 | 0 | 0 | 0 |
| 4.2.2.2 | g__Vogesella | 0 | 0 | 0 | 0 | 0 | 0 |
| 4.2.2.2 | g__Sphingomonas | 0 | 0 | 0 | 0 | 0 | 0 |
| 4.2.2.2 | g__Cellvibrio | 34 | 22 | 26 | 72 | 84 | 58 |
| 4.2.2.2 | g__unclassified_o__Solirubrobacterales | 0 | 0 | 0 | 0 | 0 | 0 |
| 4.2.2.2 | g__Arthrobacter | 2 | 2 | 0 | 10 | 2 | 8 |
| 4.2.2.2 | g__Sphingobium | 0 | 0 | 0 | 0 | 0 | 0 |
| 4.2.2.2 | g__Sphingopyxis | 0 | 0 | 0 | 0 | 0 | 0 |
| 4.2.2.2 | g__Variovorax | 0 | 0 | 0 | 0 | 0 | 0 |
| 4.2.2.2 | g__Nitrospira | 0 | 0 | 0 | 0 | 0 | 0 |
| 4.2.2.2 | g__unclassified_p__Chloroflexi | 0 | 0 | 0 | 0 | 0 | 0 |
| 4.2.2.2 | g__Gaiella | 0 | 0 | 0 | 0 | 0 | 0 |
| 4.2.2.2 | g__Enterobacter | 0 | 0 | 0 | 0 | 0 | 0 |
| 4.2.2.2 | g__Phyllobacterium | 0 | 0 | 0 | 0 | 0 | 0 |
| 4.2.2.2 | g__Acinetobacter | 0 | 0 | 0 | 0 | 0 | 0 |
| 4.2.2.2 | others | 24 | 74 | 12 | 152 | 80 | 118 |
| 3.2.1.139 | g__Pseudomonas | 0 | 0 | 0 | 0 | 0 | 0 |
| 3.2.1.139 | g__Lelliottia | 0 | 0 | 0 | 0 | 0 | 0 |
| 3.2.1.139 | g__Nocardioides | 0 | 0 | 0 | 0 | 0 | 0 |
| 3.2.1.139 | g__Pseudoxanthomonas | 26 | 36 | 32 | 176 | 160 | 202 |
| 3.2.1.139 | g__Achromobacter | 0 | 0 | 0 | 0 | 0 | 0 |
| 3.2.1.139 | g__Solirubrobacter | 0 | 0 | 0 | 0 | 0 | 0 |
| 3.2.1.139 | g__Delftia | 0 | 0 | 0 | 0 | 0 | 0 |
| 3.2.1.139 | g__Acidovorax | 0 | 0 | 0 | 0 | 0 | 0 |
| 3.2.1.139 | g__Streptomyces | 6 | 8 | 2 | 8 | 4 | 4 |
| 3.2.1.139 | g__Conexibacter | 0 | 0 | 0 | 0 | 0 | 0 |
| 3.2.1.139 | g__Azospirillum | 0 | 0 | 0 | 0 | 0 | 0 |
| 3.2.1.139 | g__Rubrobacter | 0 | 0 | 0 | 0 | 0 | 0 |
| 3.2.1.139 | g__Lysobacter | 12 | 12 | 12 | 22 | 24 | 14 |
| 3.2.1.139 | g__Flavobacterium | 32 | 22 | 2 | 72 | 26 | 50 |
| 3.2.1.139 | g__Rhizobium | 0 | 0 | 0 | 0 | 0 | 0 |
| 3.2.1.139 | g__Agrobacterium | 0 | 0 | 0 | 0 | 0 | 0 |
| 3.2.1.139 | g__Vogesella | 0 | 0 | 0 | 0 | 0 | 0 |
| 3.2.1.139 | g__Sphingomonas | 54 | 52 | 34 | 62 | 88 | 66 |
| 3.2.1.139 | g__Cellvibrio | 24 | 28 | 12 | 130 | 98 | 86 |
| 3.2.1.139 | g__unclassified_o__Solirubrobacterales | 0 | 0 | 0 | 0 | 0 | 0 |
| 3.2.1.139 | g__Arthrobacter | 0 | 0 | 0 | 0 | 0 | 0 |
| 3.2.1.139 | g__Sphingobium | 0 | 0 | 0 | 0 | 0 | 0 |
| 3.2.1.139 | g__Sphingopyxis | 0 | 6 | 2 | 6 | 8 | 2 |
| 3.2.1.139 | g__Variovorax | 0 | 0 | 0 | 0 | 0 | 0 |
| 3.2.1.139 | g__Nitrospira | 0 | 0 | 0 | 0 | 0 | 0 |
| 3.2.1.139 | g__unclassified_p__Chloroflexi | 0 | 0 | 0 | 0 | 0 | 0 |
| 3.2.1.139 | g__Gaiella | 0 | 0 | 0 | 0 | 0 | 0 |
| 3.2.1.139 | g__Enterobacter | 0 | 0 | 0 | 0 | 0 | 0 |
| 3.2.1.139 | g__Phyllobacterium | 0 | 0 | 0 | 0 | 0 | 0 |
| 3.2.1.139 | g__Acinetobacter | 0 | 0 | 0 | 0 | 0 | 0 |
| 3.2.1.139 | others | 60 | 70 | 56 | 136 | 170 | 130 |
| 3.1.1.11 | g__Pseudomonas | 0 | 0 | 0 | 0 | 0 | 0 |
| 3.1.1.11 | g__Lelliottia | 0 | 2 | 0 | 296 | 312 | 338 |
| 3.1.1.11 | g__Nocardioides | 0 | 0 | 0 | 0 | 0 | 0 |
| 3.1.1.11 | g__Pseudoxanthomonas | 0 | 0 | 0 | 0 | 0 | 0 |
| 3.1.1.11 | g__Achromobacter | 0 | 0 | 0 | 0 | 0 | 0 |
| 3.1.1.11 | g__Solirubrobacter | 0 | 0 | 0 | 0 | 0 | 0 |
| 3.1.1.11 | g__Delftia | 0 | 0 | 0 | 0 | 0 | 0 |
| 3.1.1.11 | g__Acidovorax | 0 | 0 | 0 | 0 | 0 | 0 |
| 3.1.1.11 | g__Streptomyces | 24 | 12 | 16 | 2 | 12 | 6 |
| 3.1.1.11 | g__Conexibacter | 0 | 0 | 0 | 0 | 0 | 0 |
| 3.1.1.11 | g__Azospirillum | 0 | 0 | 0 | 0 | 0 | 0 |
| 3.1.1.11 | g__Rubrobacter | 0 | 0 | 0 | 0 | 0 | 0 |
| 3.1.1.11 | g__Lysobacter | 4 | 2 | 0 | 2 | 6 | 6 |
| 3.1.1.11 | g__Flavobacterium | 56 | 56 | 60 | 156 | 108 | 72 |
| 3.1.1.11 | g__Rhizobium | 0 | 0 | 0 | 0 | 0 | 0 |
| 3.1.1.11 | g__Agrobacterium | 0 | 0 | 0 | 0 | 0 | 0 |
| 3.1.1.11 | g__Vogesella | 0 | 0 | 0 | 0 | 0 | 0 |
| 3.1.1.11 | g__Sphingomonas | 2 | 0 | 0 | 2 | 2 | 0 |
| 3.1.1.11 | g__Cellvibrio | 16 | 14 | 4 | 44 | 38 | 26 |
| 3.1.1.11 | g__unclassified_o__Solirubrobacterales | 0 | 0 | 0 | 0 | 0 | 0 |
| 3.1.1.11 | g__Arthrobacter | 2 | 0 | 6 | 16 | 8 | 2 |
| 3.1.1.11 | g__Sphingobium | 0 | 0 | 0 | 0 | 0 | 0 |
| 3.1.1.11 | g__Sphingopyxis | 0 | 0 | 0 | 0 | 0 | 0 |
| 3.1.1.11 | g__Variovorax | 0 | 0 | 0 | 0 | 0 | 0 |
| 3.1.1.11 | g__Nitrospira | 0 | 0 | 0 | 0 | 0 | 0 |
| 3.1.1.11 | g__unclassified_p__Chloroflexi | 0 | 0 | 0 | 0 | 0 | 0 |
| 3.1.1.11 | g__Gaiella | 0 | 0 | 0 | 0 | 0 | 0 |
| 3.1.1.11 | g__Enterobacter | 0 | 0 | 0 | 4 | 6 | 48 |
| 3.1.1.11 | g__Phyllobacterium | 0 | 0 | 0 | 0 | 0 | 0 |
| 3.1.1.11 | g__Acinetobacter | 0 | 0 | 0 | 0 | 0 | 0 |
| 3.1.1.11 | others | 2 | 12 | 26 | 62 | 74 | 84 |
