# Supplementary material for: A microbial consortium‐based product promotes potato yield by recruiting rhizosphere bacteria involved in nitrogen and carbon metabolisms
Source: Microb Biotechnol. 2021 Jul 7;14(5):1961–75. doi: 10.1111/1751-7915.13876 (PMC8449676; doi:10.1111/1751-7915.13876)
Supplement: Supplementary file 1 — Fig.␣S1. Tuber yield (A), soil organic carbon (B) and total nitrogen (C) in untreated (CK) plots and plots treated with 300 (B300) kg ha−1 of the MCB product containing a consortium of Bacillus subtilis and Trichoderma harzianum. T1CK and T1B300 indicate bulk soil samples collected from CK and B300 blocks, respectively, 3 days prior to planting. T2CK and T2B300 indicate rhizosphere soil samples collected from CK and B300 blocks, respectively, at the time of early tuber formation. Data represent the mean ± standard deviation (n = 3). Significant differences between treatments and the control were determined by ANOVA. Significantly different means (P < 0.05) are indicated by different letters above each bar. Fig.␣S2. Comparative analysis of dominant bacterial taxa in bulk and potato rhizosphere soil samples collected from untreated (CK) and MCB product (300 kg ha−1) treatment plots. (A) Relative abundance of the most abundant bacterial orders. (B) Bacterial orders with different relative abundance. Taxonomic profile of bacterial orders whose abundance was significantly different between CK plots and plots treated with MCB product (300 kg ha−1) 3 days prior to planting (T1, upper panel) and early tuber formation (T2, lower panel). (C) Relative abundance of the most abundant bacterial genera. (D) Bacterial genera with different relative abundance. Taxonomic profile of bacterial genera whose abundance was significantly different between CK plots and plots treated with MCB product (300 kg ha−1) 3 days prior to planting (T1, upper panel) and early tuber formation (T2, lower panel). *, ** and *** indicate a significant correlation at P < 0.05, P < 0.01 and P < 0.001 respectively. T1CK and T1B300 indicate bulk soil samples collected from CK and B300 blocks, respectively, 3 days prior to planting. T2CK and T2B300 indicate rhizosphere soil samples collected from untreated and MCB product (300 kg ha−1) treatment plots, respectively, at the time of early tuber formation. Fig.␣S3 [file MBT2-14-1961-s001.zip › mbt213876-sup-0001-supinfo/mbt213876-sup-0010-Caption.docx]

Fig. S1. Tuber yield (A), soil organic carbon (B) and total nitrogen (C) in untreated (CK) plots and plots treated with 300 (B300) kg·ha^−1^ of the MCB product containing a consortium of *Bacillus subtilis* and *Trichoderma harzianum*. T1CK and T1B300 indicate bulk soil samples collected from CK and B300 blocks, respectively, three days prior to planting. T2CK and T2B300 indicate rhizosphere soil samples collected from CK and B300 blocks, respectively, at the time of early tuber formation. Data represent the mean ± standard deviation (n = 3). Significant differences between treatments and the control were determined by ANOVA. Significantly different means (p < 0.05) are indicated by different letters above each bar.

Fig. S2. Comparative analysis of dominant bacterial taxa in bulk and potato rhizosphere soil samples collected from untreated (CK) and MCB product (300 kg·ha^−1^) treatment plots. (A) Relative abundance of the most abundant bacterial orders. (B) Bacterial orders with different relative abundance. Taxonomic profile of bacterial orders whose abundance was significantly different between CK plots and plots treated with MCB product (300 kg·ha^−1^) three days prior to planting (T1, upper panel) and early tuber formation (T2, lower panel). (C) Relative abundance of the most abundant bacterial genera. (D) Bacterial genera with different relative abundance. Taxonomic profile of bacterial genera whose abundance was significantly different between CK plots and plots treated with MCB product (300 kg·ha^−1^) three days prior to planting (T1, upper panel) and early tuber formation (T2, lower panel). *, ** and *** indicate a significant correlation at *p* < 0.05, *p* < 0.01 and *p* < 0.001 respectively. T1CK and T1B300 indicate bulk soil samples collected from CK and B300 blocks, respectively, three days prior to planting. T2CK and T2B300 indicate rhizosphere soil samples collected from untreated and MCB product (300 kg·ha^−1^) treatment plots, respectively, at the time of early tuber formation.

Fig. S3. An unweighted paired group method with arithmetic mean (UPGMA) tree analysis of bacterial community composition. The UPGMA tree was constructed based on “Bray–Curtis” distances between bacterial communities in each treatment. T1CK and T1B300 indicate bulk soil samples collected from untreated (CK) and MCB product (300 kg·ha^−1^) treatment plots, respectively, three days prior to planting. T2CK and T2B300 indicate rhizosphere soil samples collected from CK and B300 (300 kg·ha^−1^ MCB) plots, respectively, at the time of early tuber formation.

Fig. S4. Venn diagrams indicating the number of bacterial genera (A), fungal genera (B) and archaeal genera (C) in bulk and potato rhizosphere soil samples collected from untreated plots and plots treated with the MCB product. T1CK and T1B300 indicate bulk soil samples collected from CK and B300 plots, respectively, three days prior to planting. T2CK and T2B300 indicate rhizosphere soil samples collected from plots treated with 0 and 300 kg·ha^−1^ MCB product, respectively, at the time of early tuber formation.

Fig. S5. Comparative analysis of the dominant fungal communities in bulk and potato rhizosphere soil samples collected from untreated (CK) and MCB product (300 kg·ha^−1^) treatment plots respectively. (A) Relative abundance of the most abundant fungal orders. (B) Fungal orders with different relative abundance. Taxonomic profile of fungal orders with a significantly different relative abundance between 300 kg·ha^−1^ MCB product treatment and CK plots three days prior to planting (T1, upper panel) and early tuber formation (T2, lower panel). (C) Relative abundance of the most abundant fungal genera. (D) Fungal genera with different relative abundance. Taxonomic profile of fungal genera with a significant difference between 300 kg·ha^−1^ MCB product treatment and CK plots three days prior to planting (T1, upper panel) and early tuber formation (T2, lower panel). *, ** and *** indicate a significant correlation at *p* < 0.05, *p* < 0.01 and *p* < 0.001 respectively. T1CK and T1B300 indicate bulk soil samples collected from CK and B300 plots, respectively, three days prior to planting. T2CK and T2B300 indicate rhizosphere soil samples collected from untreated (CK) and MCB product (300 kg·ha^−1^) treatment plots, respectively, at the time of early tuber formation.

Fig. S6. Comparative analysis of dominant archaeal communities in bulk and potato rhizosphere soil samples collected from untreated (CK) and MCB product (300 kg·ha^−1^) treatment plots. (A) Relative abundance of the most abundant archaeal orders. (B) Archaeal orders with different relative abundance. Taxonomic profile of archaeal orders with significantly different relative abundance between the 300 kg·ha^−1^ MCB product treatment and CK plots three days prior to planting (T1, upper panel) and early tuber formation (T2, lower panel). (C) Relative abundance of the most abundant archaeal genera. (D) Archaeal genera with different relative abundance. Taxonomic profile of archaeal genera with a significant difference between 300 kg·ha^−1^ MCB product treatment and CK plots three days prior to planting (T1, upper panel) and early tuber formation (T2, lower panel). *, ** and *** indicate a significant correlation at *p* < 0.05, *p* < 0.01 and *p* < 0.001 respectively. T1CK and T1B300 indicate bulk soil samples collected from CK and B300 plots, respectively, three days prior to planting. T2CK and T2B300 indicate rhizosphere soil samples collected from untreated (CK) and MCB product (300 kg·ha^−1^) treatment plots, respectively, at the time of early tuber formation.

Fig. S7. Relationships among reads in the taxa of the bacterial community in potato bulk soil samples. (A) Co-occurrence networks in datasets obtained from CK plots. (B) Co-occurrence networks in datasets obtained from MCB product (300 kg·ha^−1^) treatment plots. Each node represents a bacterial phylotype, whereas the edges represent significant correlations, with a magnitude >+ 0.85 (positive correlation-red edges) or <– 0.85 (negative correlation-blue edges) between the nodes. Each node is labelled at the phylum level. The size of each node is proportional to the number of connections.

Fig. S8. Distribution of bacterial taxa contributing to the significantly increased abundance of genes encoding key enzymes in nitrogen metabolism pathways at the order level (A) and genera level (B); in carbon fixation pathways at the order level (C) and genera level (D); in carbon degradation pathways at order level (E) and genera level (F). T2CK and T2B300 indicate rhizosphere soil samples collected from untreated (CK) plots and MCB product (300 kg·ha^−1^) treatment plots, respectively, at the time of early tuber formation.
