# Supplementary figures and images for: A microbial consortium‐based product promotes potato yield by recruiting rhizosphere bacteria involved in nitrogen and carbon metabolisms
Source: Microb Biotechnol. 2021 Jul 7;14(5):1961–75. doi: 10.1111/1751-7915.13876 (PMC8449676; doi:10.1111/1751-7915.13876)

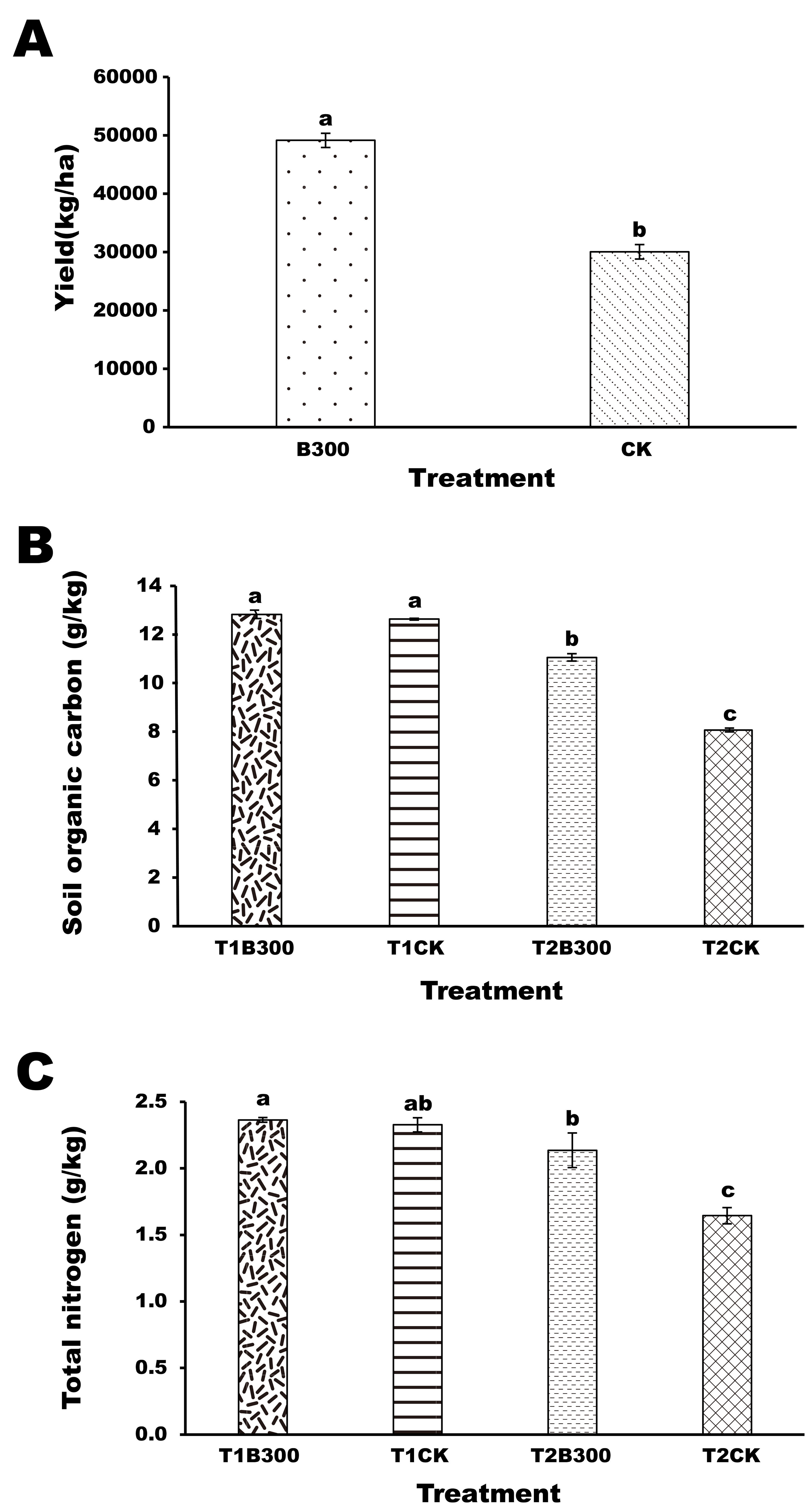

Supplement: Supplementary file 1 — Fig.␣S1. Tuber yield (A), soil organic carbon (B) and total nitrogen (C) in untreated (CK) plots and plots treated with 300 (B300) kg ha−1 of the MCB product containing a consortium of Bacillus subtilis and Trichoderma harzianum. T1CK and T1B300 indicate bulk soil samples collected from CK and B300 blocks, respectively, 3 days prior to planting. T2CK and T2B300 indicate rhizosphere soil samples collected from CK and B300 blocks, respectively, at the time of early tuber formation. Data represent the mean ± standard deviation (n = 3). Significant differences between treatments and the control were determined by ANOVA. Significantly different means (P < 0.05) are indicated by different letters above each bar. Fig.␣S2. Comparative analysis of dominant bacterial taxa in bulk and potato rhizosphere soil samples collected from untreated (CK) and MCB product (300 kg ha−1) treatment plots. (A) Relative abundance of the most abundant bacterial orders. (B) Bacterial orders with different relative abundance. Taxonomic profile of bacterial orders whose abundance was significantly different between CK plots and plots treated with MCB product (300 kg ha−1) 3 days prior to planting (T1, upper panel) and early tuber formation (T2, lower panel). (C) Relative abundance of the most abundant bacterial genera. (D) Bacterial genera with different relative abundance. Taxonomic profile of bacterial genera whose abundance was significantly different between CK plots and plots treated with MCB product (300 kg ha−1) 3 days prior to planting (T1, upper panel) and early tuber formation (T2, lower panel). *, ** and *** indicate a significant correlation at P < 0.05, P < 0.01 and P < 0.001 respectively. T1CK and T1B300 indicate bulk soil samples collected from CK and B300 blocks, respectively, 3 days prior to planting. T2CK and T2B300 indicate rhizosphere soil samples collected from untreated and MCB product (300 kg ha−1) treatment plots, respectively, at the time of early tuber formation. Fig.␣S3 [file MBT2-14-1961-s001.zip › mbt213876-sup-0001-supinfo/mbt213876-sup-0001-FigS1.tif]

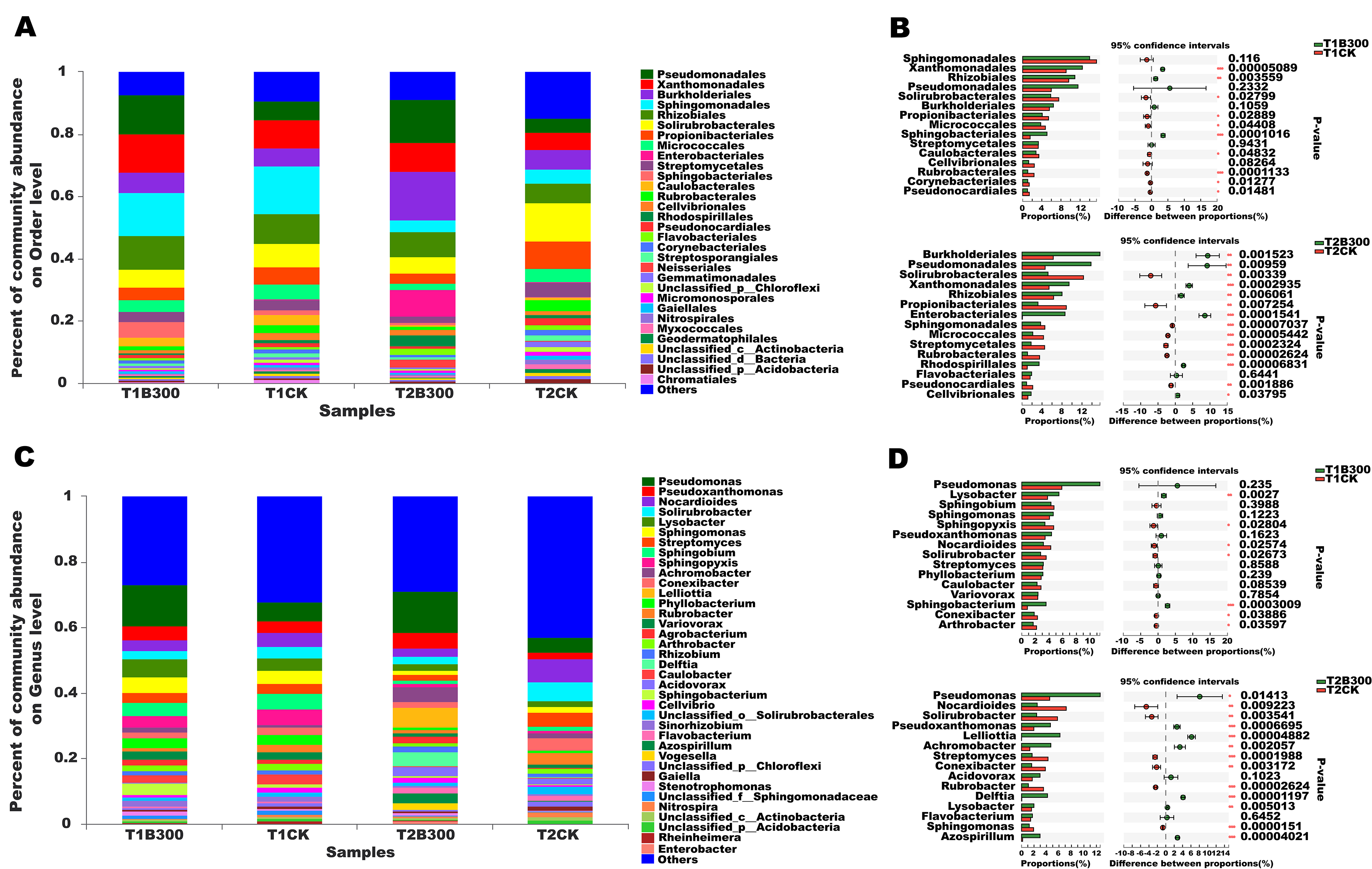

Supplement: Supplementary file 1 — Fig.␣S1. Tuber yield (A), soil organic carbon (B) and total nitrogen (C) in untreated (CK) plots and plots treated with 300 (B300) kg ha−1 of the MCB product containing a consortium of Bacillus subtilis and Trichoderma harzianum. T1CK and T1B300 indicate bulk soil samples collected from CK and B300 blocks, respectively, 3 days prior to planting. T2CK and T2B300 indicate rhizosphere soil samples collected from CK and B300 blocks, respectively, at the time of early tuber formation. Data represent the mean ± standard deviation (n = 3). Significant differences between treatments and the control were determined by ANOVA. Significantly different means (P < 0.05) are indicated by different letters above each bar. Fig.␣S2. Comparative analysis of dominant bacterial taxa in bulk and potato rhizosphere soil samples collected from untreated (CK) and MCB product (300 kg ha−1) treatment plots. (A) Relative abundance of the most abundant bacterial orders. (B) Bacterial orders with different relative abundance. Taxonomic profile of bacterial orders whose abundance was significantly different between CK plots and plots treated with MCB product (300 kg ha−1) 3 days prior to planting (T1, upper panel) and early tuber formation (T2, lower panel). (C) Relative abundance of the most abundant bacterial genera. (D) Bacterial genera with different relative abundance. Taxonomic profile of bacterial genera whose abundance was significantly different between CK plots and plots treated with MCB product (300 kg ha−1) 3 days prior to planting (T1, upper panel) and early tuber formation (T2, lower panel). *, ** and *** indicate a significant correlation at P < 0.05, P < 0.01 and P < 0.001 respectively. T1CK and T1B300 indicate bulk soil samples collected from CK and B300 blocks, respectively, 3 days prior to planting. T2CK and T2B300 indicate rhizosphere soil samples collected from untreated and MCB product (300 kg ha−1) treatment plots, respectively, at the time of early tuber formation. Fig.␣S3 [file MBT2-14-1961-s001.zip › mbt213876-sup-0001-supinfo/mbt213876-sup-0002-FigS2.tif]

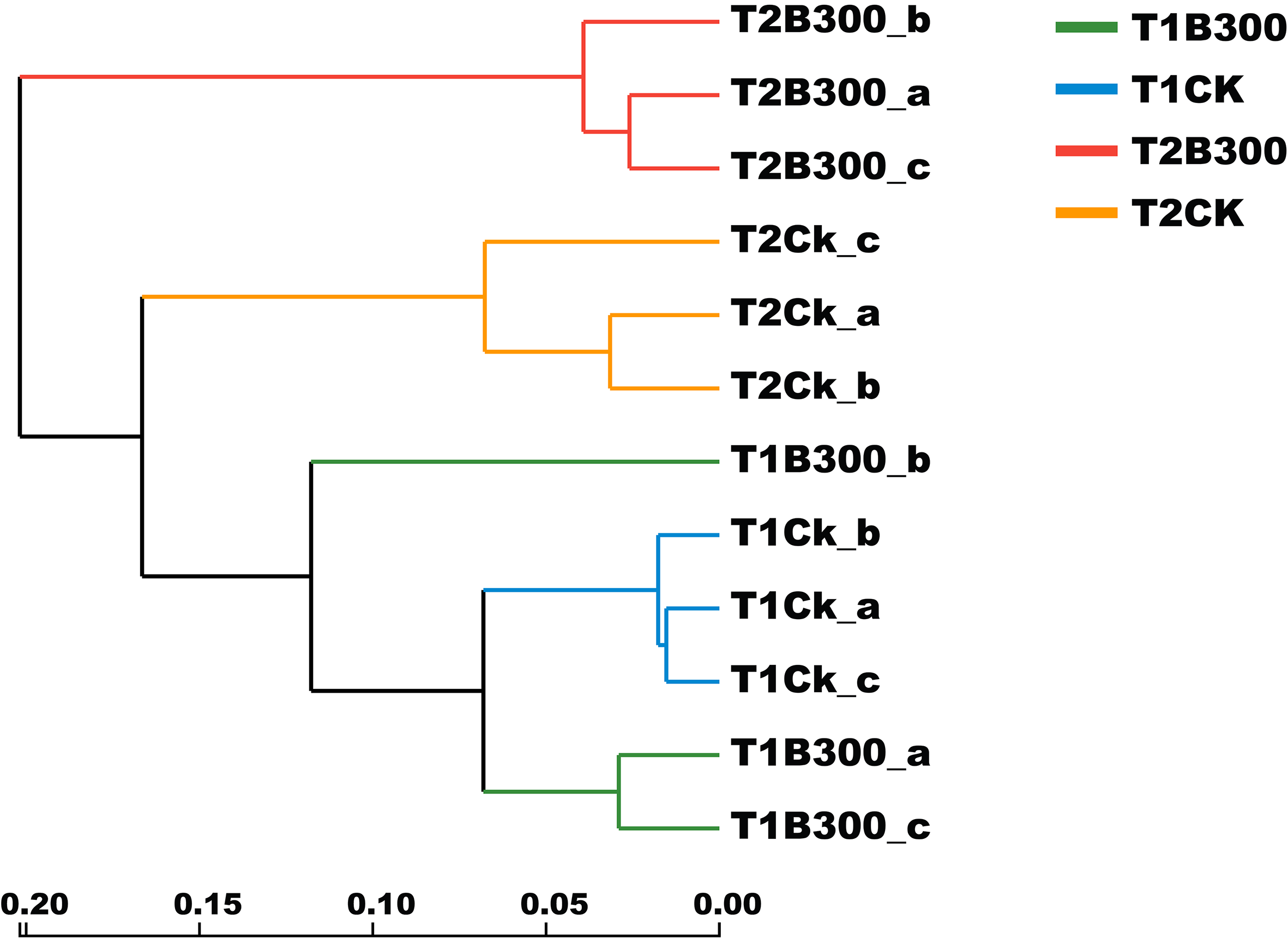

Supplement: Supplementary file 1 — Fig.␣S1. Tuber yield (A), soil organic carbon (B) and total nitrogen (C) in untreated (CK) plots and plots treated with 300 (B300) kg ha−1 of the MCB product containing a consortium of Bacillus subtilis and Trichoderma harzianum. T1CK and T1B300 indicate bulk soil samples collected from CK and B300 blocks, respectively, 3 days prior to planting. T2CK and T2B300 indicate rhizosphere soil samples collected from CK and B300 blocks, respectively, at the time of early tuber formation. Data represent the mean ± standard deviation (n = 3). Significant differences between treatments and the control were determined by ANOVA. Significantly different means (P < 0.05) are indicated by different letters above each bar. Fig.␣S2. Comparative analysis of dominant bacterial taxa in bulk and potato rhizosphere soil samples collected from untreated (CK) and MCB product (300 kg ha−1) treatment plots. (A) Relative abundance of the most abundant bacterial orders. (B) Bacterial orders with different relative abundance. Taxonomic profile of bacterial orders whose abundance was significantly different between CK plots and plots treated with MCB product (300 kg ha−1) 3 days prior to planting (T1, upper panel) and early tuber formation (T2, lower panel). (C) Relative abundance of the most abundant bacterial genera. (D) Bacterial genera with different relative abundance. Taxonomic profile of bacterial genera whose abundance was significantly different between CK plots and plots treated with MCB product (300 kg ha−1) 3 days prior to planting (T1, upper panel) and early tuber formation (T2, lower panel). *, ** and *** indicate a significant correlation at P < 0.05, P < 0.01 and P < 0.001 respectively. T1CK and T1B300 indicate bulk soil samples collected from CK and B300 blocks, respectively, 3 days prior to planting. T2CK and T2B300 indicate rhizosphere soil samples collected from untreated and MCB product (300 kg ha−1) treatment plots, respectively, at the time of early tuber formation. Fig.␣S3 [file MBT2-14-1961-s001.zip › mbt213876-sup-0001-supinfo/mbt213876-sup-0003-FigS3.tif]

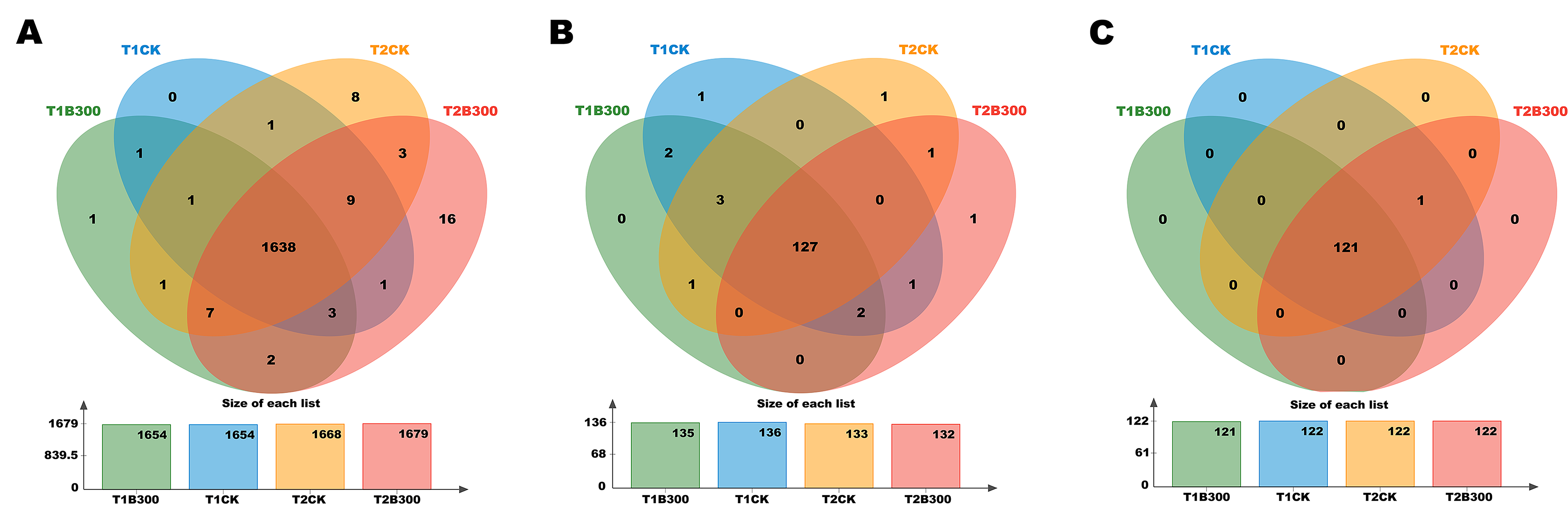

Supplement: Supplementary file 1 — Fig.␣S1. Tuber yield (A), soil organic carbon (B) and total nitrogen (C) in untreated (CK) plots and plots treated with 300 (B300) kg ha−1 of the MCB product containing a consortium of Bacillus subtilis and Trichoderma harzianum. T1CK and T1B300 indicate bulk soil samples collected from CK and B300 blocks, respectively, 3 days prior to planting. T2CK and T2B300 indicate rhizosphere soil samples collected from CK and B300 blocks, respectively, at the time of early tuber formation. Data represent the mean ± standard deviation (n = 3). Significant differences between treatments and the control were determined by ANOVA. Significantly different means (P < 0.05) are indicated by different letters above each bar. Fig.␣S2. Comparative analysis of dominant bacterial taxa in bulk and potato rhizosphere soil samples collected from untreated (CK) and MCB product (300 kg ha−1) treatment plots. (A) Relative abundance of the most abundant bacterial orders. (B) Bacterial orders with different relative abundance. Taxonomic profile of bacterial orders whose abundance was significantly different between CK plots and plots treated with MCB product (300 kg ha−1) 3 days prior to planting (T1, upper panel) and early tuber formation (T2, lower panel). (C) Relative abundance of the most abundant bacterial genera. (D) Bacterial genera with different relative abundance. Taxonomic profile of bacterial genera whose abundance was significantly different between CK plots and plots treated with MCB product (300 kg ha−1) 3 days prior to planting (T1, upper panel) and early tuber formation (T2, lower panel). *, ** and *** indicate a significant correlation at P < 0.05, P < 0.01 and P < 0.001 respectively. T1CK and T1B300 indicate bulk soil samples collected from CK and B300 blocks, respectively, 3 days prior to planting. T2CK and T2B300 indicate rhizosphere soil samples collected from untreated and MCB product (300 kg ha−1) treatment plots, respectively, at the time of early tuber formation. Fig.␣S3 [file MBT2-14-1961-s001.zip › mbt213876-sup-0001-supinfo/mbt213876-sup-0004-FigS4.tif]

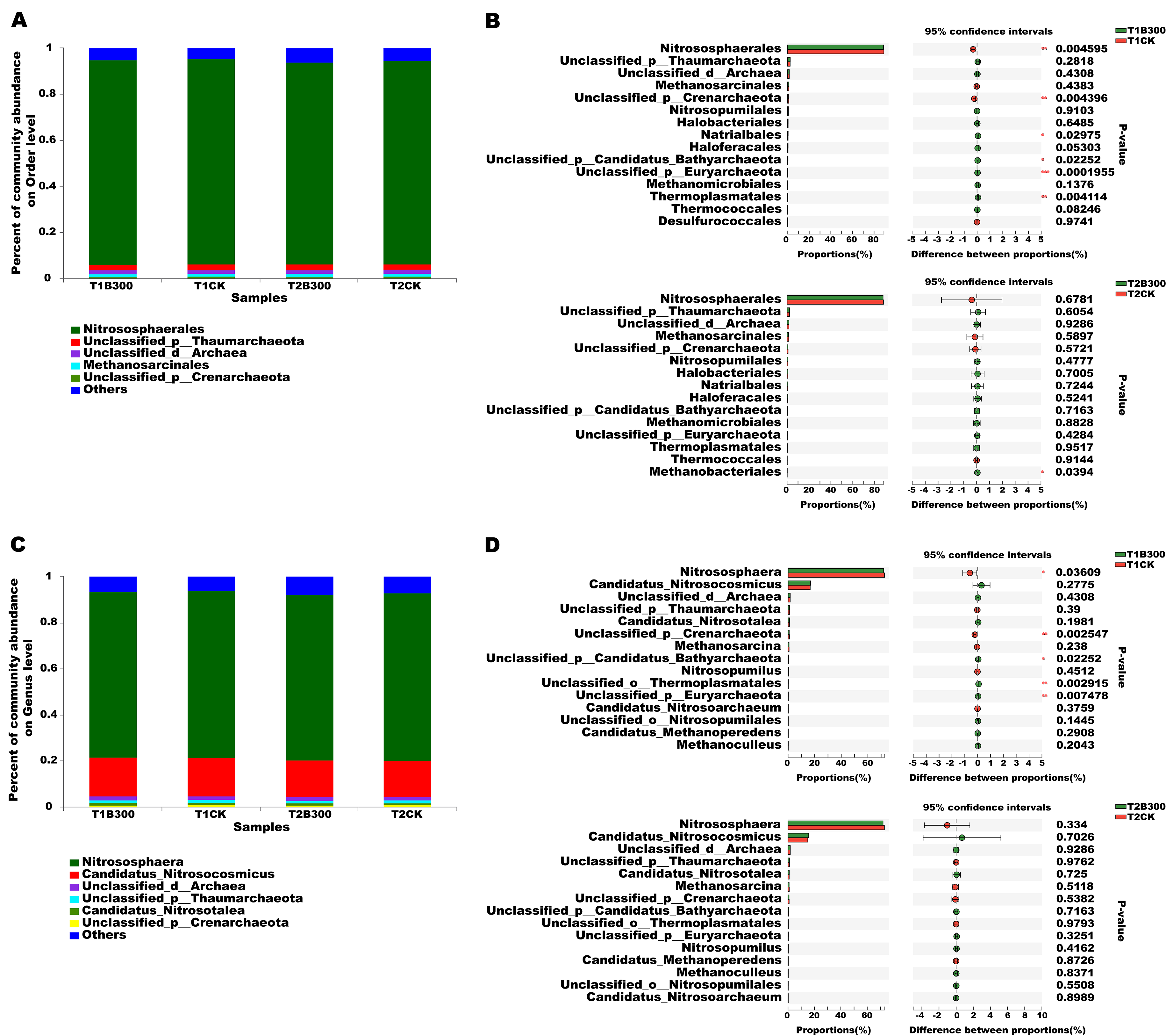

Supplement: Supplementary file 1 — Fig.␣S1. Tuber yield (A), soil organic carbon (B) and total nitrogen (C) in untreated (CK) plots and plots treated with 300 (B300) kg ha−1 of the MCB product containing a consortium of Bacillus subtilis and Trichoderma harzianum. T1CK and T1B300 indicate bulk soil samples collected from CK and B300 blocks, respectively, 3 days prior to planting. T2CK and T2B300 indicate rhizosphere soil samples collected from CK and B300 blocks, respectively, at the time of early tuber formation. Data represent the mean ± standard deviation (n = 3). Significant differences between treatments and the control were determined by ANOVA. Significantly different means (P < 0.05) are indicated by different letters above each bar. Fig.␣S2. Comparative analysis of dominant bacterial taxa in bulk and potato rhizosphere soil samples collected from untreated (CK) and MCB product (300 kg ha−1) treatment plots. (A) Relative abundance of the most abundant bacterial orders. (B) Bacterial orders with different relative abundance. Taxonomic profile of bacterial orders whose abundance was significantly different between CK plots and plots treated with MCB product (300 kg ha−1) 3 days prior to planting (T1, upper panel) and early tuber formation (T2, lower panel). (C) Relative abundance of the most abundant bacterial genera. (D) Bacterial genera with different relative abundance. Taxonomic profile of bacterial genera whose abundance was significantly different between CK plots and plots treated with MCB product (300 kg ha−1) 3 days prior to planting (T1, upper panel) and early tuber formation (T2, lower panel). *, ** and *** indicate a significant correlation at P < 0.05, P < 0.01 and P < 0.001 respectively. T1CK and T1B300 indicate bulk soil samples collected from CK and B300 blocks, respectively, 3 days prior to planting. T2CK and T2B300 indicate rhizosphere soil samples collected from untreated and MCB product (300 kg ha−1) treatment plots, respectively, at the time of early tuber formation. Fig.␣S3 [file MBT2-14-1961-s001.zip › mbt213876-sup-0001-supinfo/mbt213876-sup-0006-FigS6.tif]

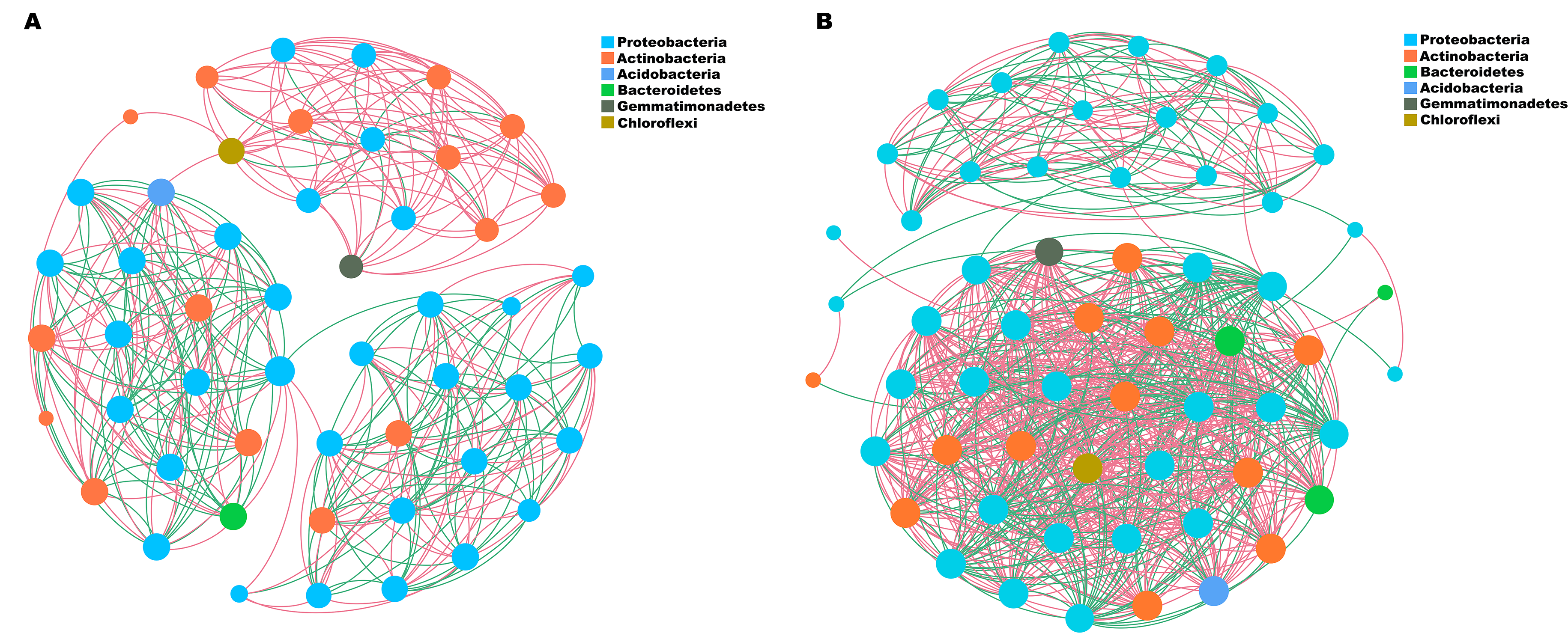

Supplement: Supplementary file 1 — Fig.␣S1. Tuber yield (A), soil organic carbon (B) and total nitrogen (C) in untreated (CK) plots and plots treated with 300 (B300) kg ha−1 of the MCB product containing a consortium of Bacillus subtilis and Trichoderma harzianum. T1CK and T1B300 indicate bulk soil samples collected from CK and B300 blocks, respectively, 3 days prior to planting. T2CK and T2B300 indicate rhizosphere soil samples collected from CK and B300 blocks, respectively, at the time of early tuber formation. Data represent the mean ± standard deviation (n = 3). Significant differences between treatments and the control were determined by ANOVA. Significantly different means (P < 0.05) are indicated by different letters above each bar. Fig.␣S2. Comparative analysis of dominant bacterial taxa in bulk and potato rhizosphere soil samples collected from untreated (CK) and MCB product (300 kg ha−1) treatment plots. (A) Relative abundance of the most abundant bacterial orders. (B) Bacterial orders with different relative abundance. Taxonomic profile of bacterial orders whose abundance was significantly different between CK plots and plots treated with MCB product (300 kg ha−1) 3 days prior to planting (T1, upper panel) and early tuber formation (T2, lower panel). (C) Relative abundance of the most abundant bacterial genera. (D) Bacterial genera with different relative abundance. Taxonomic profile of bacterial genera whose abundance was significantly different between CK plots and plots treated with MCB product (300 kg ha−1) 3 days prior to planting (T1, upper panel) and early tuber formation (T2, lower panel). *, ** and *** indicate a significant correlation at P < 0.05, P < 0.01 and P < 0.001 respectively. T1CK and T1B300 indicate bulk soil samples collected from CK and B300 blocks, respectively, 3 days prior to planting. T2CK and T2B300 indicate rhizosphere soil samples collected from untreated and MCB product (300 kg ha−1) treatment plots, respectively, at the time of early tuber formation. Fig.␣S3 [file MBT2-14-1961-s001.zip › mbt213876-sup-0001-supinfo/mbt213876-sup-0007-FigS7.tif]

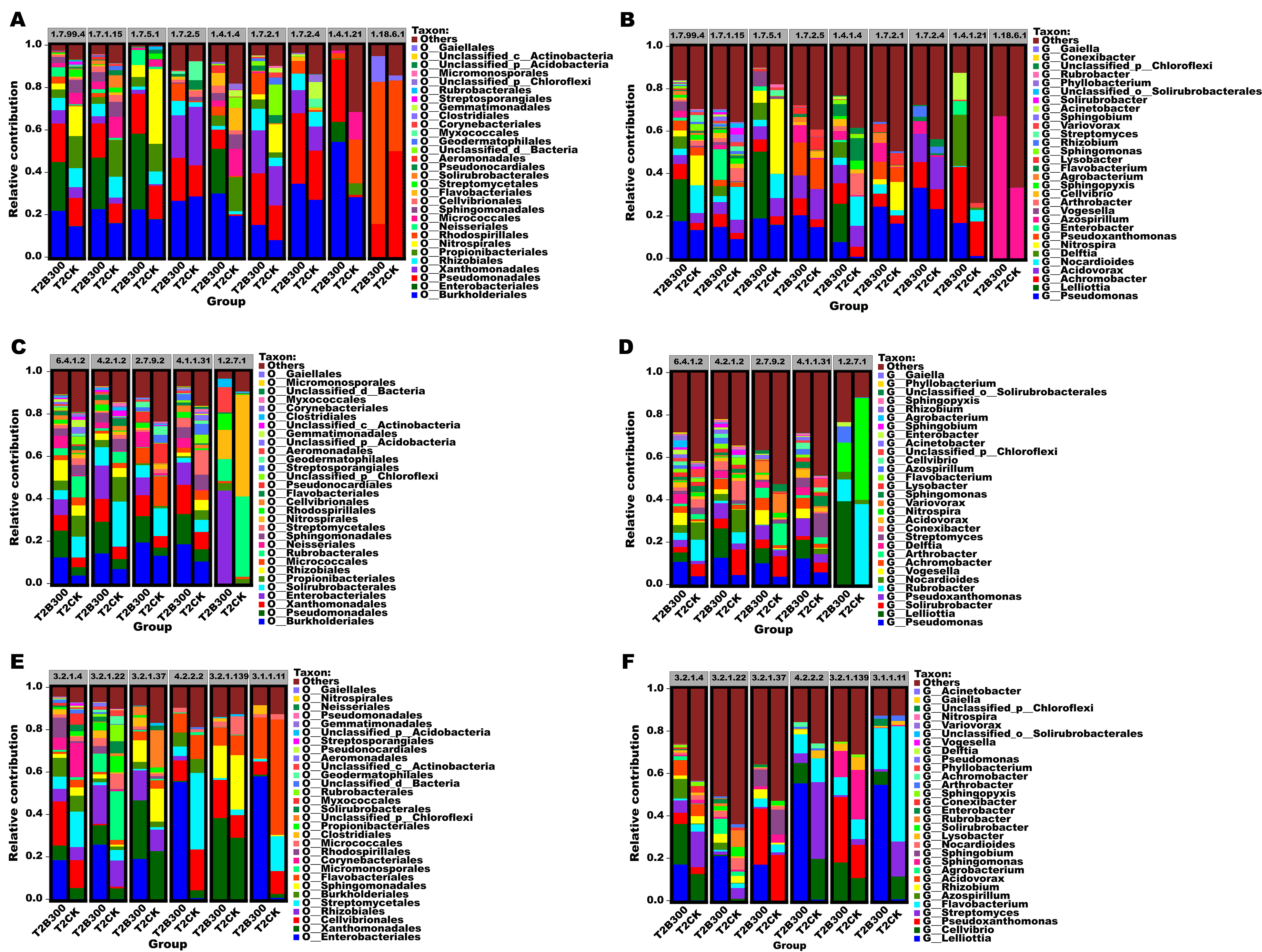

Supplement: Supplementary file 1 — Fig.␣S1. Tuber yield (A), soil organic carbon (B) and total nitrogen (C) in untreated (CK) plots and plots treated with 300 (B300) kg ha−1 of the MCB product containing a consortium of Bacillus subtilis and Trichoderma harzianum. T1CK and T1B300 indicate bulk soil samples collected from CK and B300 blocks, respectively, 3 days prior to planting. T2CK and T2B300 indicate rhizosphere soil samples collected from CK and B300 blocks, respectively, at the time of early tuber formation. Data represent the mean ± standard deviation (n = 3). Significant differences between treatments and the control were determined by ANOVA. Significantly different means (P < 0.05) are indicated by different letters above each bar. Fig.␣S2. Comparative analysis of dominant bacterial taxa in bulk and potato rhizosphere soil samples collected from untreated (CK) and MCB product (300 kg ha−1) treatment plots. (A) Relative abundance of the most abundant bacterial orders. (B) Bacterial orders with different relative abundance. Taxonomic profile of bacterial orders whose abundance was significantly different between CK plots and plots treated with MCB product (300 kg ha−1) 3 days prior to planting (T1, upper panel) and early tuber formation (T2, lower panel). (C) Relative abundance of the most abundant bacterial genera. (D) Bacterial genera with different relative abundance. Taxonomic profile of bacterial genera whose abundance was significantly different between CK plots and plots treated with MCB product (300 kg ha−1) 3 days prior to planting (T1, upper panel) and early tuber formation (T2, lower panel). *, ** and *** indicate a significant correlation at P < 0.05, P < 0.01 and P < 0.001 respectively. T1CK and T1B300 indicate bulk soil samples collected from CK and B300 blocks, respectively, 3 days prior to planting. T2CK and T2B300 indicate rhizosphere soil samples collected from untreated and MCB product (300 kg ha−1) treatment plots, respectively, at the time of early tuber formation. Fig.␣S3 [file MBT2-14-1961-s001.zip › mbt213876-sup-0001-supinfo/mbt213876-sup-0008-FigS8.tif]
